# Temporal patterns of suicidal ideation prevalence during the COVID-19 pandemic: a systematic review and meta-analysis of cross-sectional and longitudinal studies

**DOI:** 10.1017/S2045796025100358

**Published:** 2025-12-19

**Authors:** Xuefei Tao, Zhihui Zhang, Li Liang, Shen Xu, Xiayu Du, Zhihong Ren, Xianglian Yu

**Affiliations:** 1 Key Laboratory of Adolescent CyberPsychology and Behavior, CCNU, Ministry of Education, Wuhan, China; 2 Key Laboratory of Human Development and Mental Health of Hubei Province, School of Psychology, Central China Normal University, Wuhan, China; 3 School of Psychology, Liaoning Normal University, Dalian, China; 4 Department of Education, Jianghan University, Wuhan, China

**Keywords:** COVID-19, epidemiology, social factors, suicide, systematic reviews

## Abstract

**Aims:**

Although extensive research has been conducted on the impact of the COVID-19 pandemic on global mental health, a systematic synthesis of the cross-time dynamics of suicidal ideation (SI) remains lacking. This study aims to systematically synthesise the global aggregated prevalence of SI before and after the pandemic, investigate the potential association between pandemic exposure and the SI risk through meta-regression analysis of longitudinal studies, and explore key moderating factors.

**Methods:**

A systematic search was conducted in Web of Science, PubMed, PsycINFO and ProQuest databases up to August 2025. Observational studies were included if they employed cross-sectional or longitudinal designs and reported the prevalence of SI before and after the pandemic across global regions.

**Results:**

The analysis included 354 cross-sectional studies (*N* = 8,247,875) and 27 longitudinal studies. In cross-sectional studies, the pooled prevalence of SI was 13.20% [95% CI 12.06%–14.42%]. Pre-pandemic prevalence was 12.52% [95% CI 8.46%–18.14%], and post-pandemic prevalence was 13.24% [95% CI 12.07%–14.50%], with no significant difference. Meta-regression analysis identified three moderators. Specifically, larger sample sizes (*n*) were associated with lower prevalence (*β* = −0.232, *P* < 0.0001); higher study quality predicted lower prevalence (*β* = −0.278, *P* < 0.001); and studies on adults reported significantly lower prevalence than adolescents (*β* = −0.366, *P* < 0.05). Conversely, time progression during the pandemic, development level, geographical area, gender and measurement method did not show significant independent effects. Interaction analyses also found no significant moderating effect of economic development level or geographical area on the temporal trend of SI prevalence. Longitudinal analysis found no significant increase in prevalence from the pre-pandemic to the post-pandemic period (*P* = 0.101). However, a small but significant increase occurred between early and late stages within the pandemic (*β* = 0.265, *P* = 0.021). Subgroup analyses showed no significant moderation of these temporal changes.

**Conclusions:**

The COVID-19 pandemic’s impact on SI was dynamic. While no significant prevalence change was found between pre- and post-pandemic periods, a significant increase occurred as the crisis progressed. This deteriorating trend was more pronounced in adolescents, identifying them as a key vulnerable group. Methodologically, findings were moderated by the measurement instrument, study quality and sample size, with evidence suggesting potential small-study effects. These findings underscore the need for robust mental health surveillance and targeted interventions for at-risk populations during prolonged public health crises.

The protocol was registered on PROSPERO (CRD42024603151).

## Introduction

The COVID-19 pandemic, as a major public health event of the 21st century, has resulted in over 7 million cumulative reported deaths globally (World Health Organization, 2025). In the field of psychiatric epidemiology, its impact extends far beyond physical health, with growing evidence indicating that the secondary mental health consequences may exert more persistent harm. Systematic research estimates that in the first year of the pandemic, the global prevalence of anxiety disorders increased by 25.60% and depression by 27.60% (Santomauro *et al.*, 2021). Suicidal ideation (SI), as a more extreme indicator of psychological crisis, may exhibit higher prevalence than reported in pre-pandemic general population studies (Farooq *et al.*, 2021). A recent large-scale meta-analysis encompassing 202 observational studies across 41 countries further quantified this, reporting a pooled global prevalence of SI at 13.5% during the pandemic, with significant heterogeneity across studies (*I*^2^ = 99.83%; Mudiyanselage *et al.*, 2025). Yet, its epidemiological characteristics still require urgent systematic evaluation.

Although the COVID-19 pandemic has been linked to risk factors for suicidal behaviour (e.g., death anxiety, trauma exposure), there remains a significant inconsistency in evidence and methodological debate regarding its true impact on global SI prevalence. The pandemic activated individuals’ proximal defence mechanisms via pathways, including perceived threat of death, social isolation and economic shocks (Pyszczynski *et al.*, 2021). Additionally, pandemic-related uncertainty and exposure to traumatic events (e.g., loss of loved ones, economic crises) further contributed to potential risk factors for suicide. Meta-analyses have shown an average prevalence of SI of approximately 14.70% across over 30 countries during the pandemic (Du *et al.*, 2023), with higher prevalence in the general public (11%) than in healthcare workers (5.8%) (Phiri *et al.*, 2021), and even higher rates among vulnerable subgroups like young adults and transgender individuals (Mudiyanselage *et al.*, 2025). While the annual incidence rate of suicide non-significantly increased by 10% during the COVID-19 pandemic compared with the pre-pandemic period (Bersia *et al.*, 2022), the pooled prevalence of self-harm reached 15.8% during the pandemic (Cheng *et al.*, 2023). However, substantial inter-study heterogeneity exists; a systematic review reported that among 18 suicide-related studies, four documented increased suicide attempts and two reported decreases (Pathirathna *et al.*, 2022). Limitations such as small samples, regional bias and lack of longitudinal designs hinder the causal inference and precise impact quantification.

A large-scale meta-analysis can synthesise existing evidence to quantify changes in SI prevalence before and after the pandemic, as well as identify influencing factors. Therefore, this study conducted a meta-analysis of published literature and explored potential sources of between-study variance.

## Methods

### Protocol and registration

This meta-analysis adheres to the Preferred Reporting Items for Systematic Reviews and Meta-Analyses (PRISMA) guidelines (Page *et al.*, 2021); Meta-Analysis Reporting Standards (MARS) (Appelbaum *et al.*, 2018). The review protocol was registered with the International Prospective Register of Systematic Reviews (PROSPERO: CRD42024603151).

### Information sources and search strategy

Literature searches were conducted in the Web of Science, PubMed, ProQuest and PsycINFO, covering a date range from 2019 to 30 August 2025. Building on the search strategies of prior meta-analyses (Bersia *et al.*, 2022; Du *et al.*, 2023), the search strategy was designed to combine keywords related to SI with terms for the COVID-19 pandemic. The full search strategies for each database are provided in Supplementary Materials 1. Duplicates were removed using NoteExpress 4.1.0.

### Eligibility criteria

Studies were included if they were English-language empirical reports (cross-sectional or longitudinal) that provided the prevalence of SI in the context of the COVID-19 pandemic. Eligible SI outcomes were those assessed via validated psychometric scales or direct, unambiguous questioning. We excluded reviews, meta-analyses, qualitative studies, intervention trials and studies not reporting SI prevalence. A detailed breakdown of the eligibility criteria is available in Supplementary Materials 2.

### Selection process

Two authors independently screened titles/abstracts and full texts, extracted data and assessed risk of bias according to a pre-specified protocol. Discrepancies were resolved through discussion with a third author.

### Data extraction and coding procedures

Following a pre-specified protocol, two researchers independently extracted data from all included studies, with a third and fourth researcher cross-verifying the entries. Discrepancies were resolved through team discussion. Key extracted variables included study characteristics, sample demographics and outcome measures (see Supplementary Materials 3 for the full coding protocol).

To ensure data harmonisation, we established several coding rules. First, all measures of SI, regardless of reported severity, were recoded into a dichotomous variable (present/absent). Second, when multiple timeframes were reported, data from the most recent time point were prioritised to maintain consistency. Finally, key demographic moderators like age and gender were dummy-coded (Kristensen *et al.*, 2024), with separate categories for missing data. Raw data were used to calculate prevalence rates when not directly reported (see Supplementary Materials 3 for details on variable operationalisation).

### Risk of bias assessment

Two authors independently assessed the risk of bias for all included studies. We used an adapted version of the Joanna Briggs Institute Critical Appraisal Checklist (JBICAC) for cross-sectional studies and the Newcastle-Ottawa Quality Assessment Scale (NOQAS) for longitudinal studies. Higher scores indicated a lower risk of bias. All discrepancies in scoring were resolved through discussion. The adapted checklists and detailed scoring criteria are provided in Supplementary Materials 4.

### Meta-analysis strategy

This meta-analysis addresses two primary objectives: (1) estimating the overall prevalence of SI in the pre-pandemic period and during the COVID-19 pandemic; (2) comparing trends and differences in SI prevalence across pre-/post-pandemic periods and exploring potential influencing factors. We employed a two-pronged meta-analytic approach: (1) a random-effects model to pool prevalence from cross-sectional studies and (2) a meta-regression of log odds ratios (LORs) from longitudinal studies to assess temporal changes in SI.

All analyses were conducted in R Version 4.4.3 (R Core Team, 2024). We used the metafor package Version 4.8–0 (Viechtbauer, 2010) for all meta-analytic models and the ggplot2 package Version 3.5.2 (Wickham *et al.*, 2023) for data visualisation.

### Cross-sectional study

Random effects models (REML) were used to pool SI prevalence rates for pre- and post-pandemic periods, with a logit transformation applied to proportion data. Heterogeneity was assessed using the *I^2^* statistic (Higgins and Thompson, 2002), and baseline heterogeneity was estimated with a random effects model without covariates (Borenstein *et al.*, 2021). Sensitivity analyses were conducted by excluding studies with extreme sample sizes (*n* > 10,000).

Random effects meta-regression was conducted to investigate SI prevalence modifiers, with each covariate, including at least approximately 10 studies as recommended (Borenstein *et al.*, 2021). Specific covariates comprised: (1) pandemic time stage; (2) different levels of economic development; (3) geographic region; (4) age group; (5) gender; (6) study quality score; (7) sample size; (8) measurements. Furthermore, a separate sensitivity analysis on a subset of 127 studies was conducted to assess the impact of specific measurement tools, namely the Patient Health Questionnaire-9 (PHQ-9) vs. the Columbia-Suicide Severity Rating Scale (C-SSRS). Interaction terms were included to assess effect modification, specifically to examine whether the effect of one moderator on SI prevalence varied across different levels of another moderator. For instance, the time × economic development level interaction term investigated whether the temporal trend in SI prevalence differed significantly between developed and developing countries.

To illustrate the global prevalence of SI, a world map was created to depict the post-pandemic, country-level prevalence distribution.

### Longitudinal studies

To assess temporal changes in SI prevalence, longitudinal studies with repeated measurements from the same cohort were analysed. For longitudinal studies, we calculated the log odds ratio (LOR) for each study based on the change in logit-transformed prevalence between paired time points to account for the within-subject design. The variance of the LOR accounted for the intra-study correlation, which was assumed to be *r* = 0.5 in the primary analysis, with sensitivity analyses conducted at *r* = 0.3 and *r* = 0.7 (Borenstein *et al.*, 2021). LORs were pooled using a random-effects model. Pooled analyses were conducted using the rma function in the metafor package, with forest plots visualising LORs and 95% confidence intervals (CIs).

The first category of analysis involved studies that compared pre-pandemic and within-pandemic prevalence. These studies provided a baseline measurement of SI before the pandemic’s onset and a follow-up measurement during the pandemic. The resulting pooled effect size for this group quantifies the net change from the pre-pandemic period to the pandemic period. The second category of analysis focused on trends occurring entirely within the pandemic itself. This group included studies where both the initial and follow-up measurements were conducted at different stages during the pandemic. The pooled effect size for this group, therefore, reflects the evolution of SI prevalence as the public health crisis unfolded.

### Publication bias

Potential publication bias for each meta-analysis was assessed by visually inspecting funnel plot asymmetry and conducting Egger’s regression test (Egger *et al.*, 1997). Where asymmetry was present, the Duval and Tweedie trim-and-fill procedure (Duval and Tweedie, 2000a, 2000b) was used to estimate an adjusted effect size.

## Result

**Figure 1.**
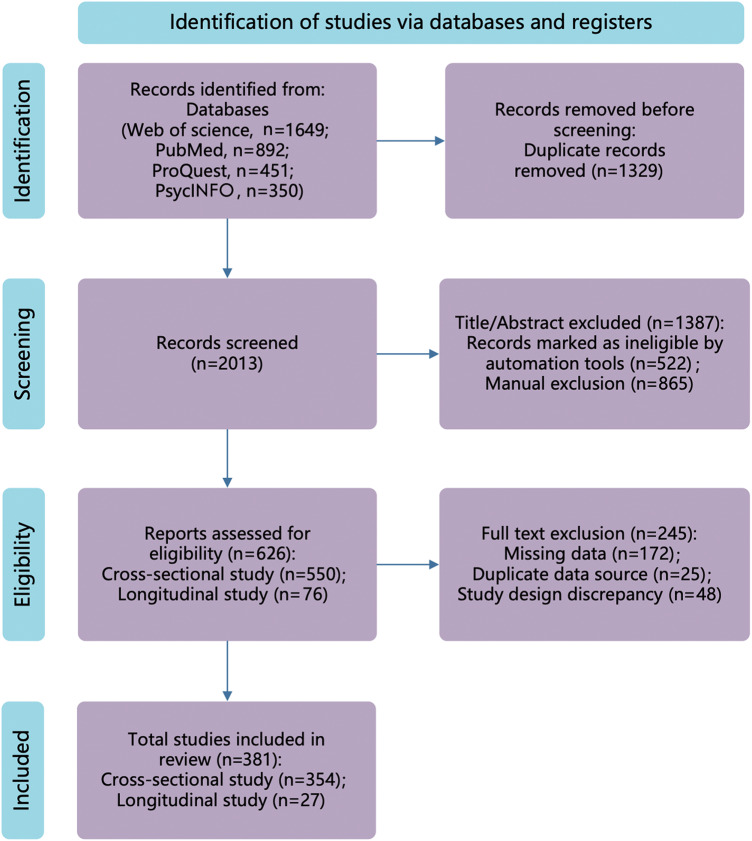
Study selection process.

The flowchart of literature search results and study screening process is presented in Fig. 1. After removing 1329 duplicates, we screened the titles and abstracts of the remaining 2013 publications, yielding 550 cross-sectional and 76 longitudinal studies for full-text review. Ultimately, 354 cross-sectional and 27 longitudinal studies met the inclusion criteria. Characteristics of included studies are presented in Table 1, while all their characteristics, along with details of excluded studies, are provided in Supplementary Materials 5.

**Table 1.** Details of included studies

Example study (citation)	Country	Studies from country (*n*)	Study design
Czeisler et al., 2021	Australia	5	Cross-sectional
Niederkrotenthaler et al., 2022	Austria	1	Cross-sectional
Kabir et al., 2023	Bangladesh	8	Cross-sectional
Reyniers et al., 2022	Belgium	2	Cross-sectional
Sljivo et al., 2022	Bosnia and Herzegovina	1	Cross-sectional
Trettel et al., 2022	Brazil	7	Cross-sectional
Daly et al., 2021	Canada	23	Cross-sectional
Ramirez et al., 2022	Chile	2	Cross-sectional
Zhang et al., 2024	China	61	Cross-sectional
Benavides Morales and López Peláez, 2022	Colombia	1	Cross-sectional
Sørensen et al., 2025	Denmark	1	Cross-sectional
Shongwe and Huang, 2021	Eswatini	1	Cross-sectional
Workneh et al., 2023	Ethiopia	2	Cross-sectional
Essadek et al., 2022	France	18	Cross-sectional
Höller and Forkmann, 2022	Germany	6	Cross-sectional
Fountoulakis et al., 2022	Greece	6	Cross-sectional
Chakrabarti, 2021	India	5	Cross-sectional
Prawira et al., 2023	Indonesia	1	Cross-sectional
Pouradeli et al., 2024	Iran	7	Cross-sectional
Brady, Fenton, et al., 2023	Ireland	5	Cross-sectional
Kleinhendler-Lustig et al., 2023	Israel	5	Cross-sectional
Rus Prelog et al., 2023	Italy	10	Cross-sectional
Yamashita et al., 2024	Japan	11	Cross-sectional
El Frenn et al., 2023	Lebanon	1	Cross-sectional
Elhadi et al., 2022	Libya	2	Cross-sectional
Žilinskas et al., 2021	Lithuania	2	Cross-sectional
Mzumara, 2024	Malawi	1	Cross-sectional
Sahimi et al., 2021	Malaysia	2	Cross-sectional
Bonello et al., 2021	Malta	1	Cross-sectional
Genis-Mendoza et al., 2021	Mexico	6	Cross-sectional
Delgadillo et al., 2023	Mixed	6	Cross-sectional
Every-Palmer et al., 2020	New Zealand	2	Cross-sectional
Fadipe et al., 2021	Nigeria	1	Cross-sectional
Sivertsen et al., 2022	Norway	2	Cross-sectional
Mushtaque et al., 2024	Pakistan	2	Cross-sectional
Valladares-Garrido et al., 2024	Peru	3	Cross-sectional
Rutkowska et al., 2022	Poland	3	Cross-sectional
AlAbdulla et al., 2022	Qatar	2	Cross-sectional
Kholmogorova et al., 2021	Russia	3	Cross-sectional
Mallhi et al., 2022	Saudi Arabia	3	Cross-sectional
Matić et al., 2022	Slovenia	3	Cross-sectional
Bantjes et al., 2023	South Africa	4	Cross-sectional
Kim et al., 2022	South Korea	15	Cross-sectional
Sanchez Merino et al., 2024	Spain	14	Cross-sectional
Wijesinghe et al., 2023	Sri Lanka	1	Cross-sectional
Dumont et al., 2024	Switzerland	1	Cross-sectional
Supasitthumrong et al., 2024	Thailand	2	Cross-sectional
Regaieg et al., 2022	Tunisia	1	Cross-sectional
Korkmaz et al., 2022	Turkey	5	Cross-sectional
Kaggwa et al., 2022	Uganda	3	Cross-sectional
John et al., 2021	United Kingdom	5	Cross-sectional
Asieieva et al., 2022	Ukraine	2	Cross-sectional
Kim et al., 2025	United States of America	61	Cross-sectional
Rogers et al., 2023	Unknown	6	Cross-sectional
López Steinmetz et al., 2021	Argentina	1	Longitudinal
Batterham et al., 2022	Australia	1	Longitudinal
Antonelli-Salgado et al., 2021	Brazil	2	Longitudinal
Gouin et al., 2023	Canada	1	Longitudinal
Huang et al., 2022	China	6	Longitudinal
Husky et al., 2024	France	1	Longitudinal
Efstathiou et al., 2022	Greece	2	Longitudinal
Ori et al., 2023	Holland	1	Longitudinal
Palagini et al., 2023	Italy	1	Longitudinal
Sasaki et al., 2021	Japan	2	Longitudinal
Domínguez-González et al., 2022	Mexico	1	Longitudinal
Ayuso-Mateos et al., 2023	Spanish	1	Longitudinal
Padmanathan et al., 2023	United Kingdom	1	Longitudinal
Danzo et al., 2024	United States of America	6	Longitudinal

Note. The reference list of the included studies is provided in Supplementary Material 9.

### Descriptive characteristics of the included studies

The 354 cross-sectional studies included 8,247,875 participants via self-report measurements or clinical assessments. These studies, published between 2019 and 2025, drew samples from 53 countries, covering diverse ages, sample sizes (33 to 2,186,037) and female proportions (0%–100%). Data sources were primarily surveys, but also included database records (*n* = 5), hospital admissions (*n* = 16) and psychological hotline data (*n* = 2).

Of the 27 longitudinal studies, 9 spanned the pre- to post-pandemic period, while 18 were conducted entirely post-pandemic. The total pre-test sample was 384,818, with 236,166 participants remaining in post-test assessments. These studies represented 14 countries across Europe, the Americas and Asia and included diverse age groups with female proportions from 38% to 85.61%.

### Assessment of the prevalence of SI

Prevalence of SI was primarily assessed via two methods: self-report questionnaires and clinical/lay interviews. Self-report scales typically utilised instruments such as the PHQ-9 and C-SSRS, while interviews often involved one or two binary questions about suicidal thoughts (e.g. ‘Have you had thoughts of suicide?’ with yes/no responses).

### Risk of bias assessment

Detailed results of risk of bias assessments are presented in Supplementary Materials 6. Cross-sectional studies were evaluated using the JBICAC, which assigns scores on a 0–7 scale (*M* = 5.376, *SD* = 0.671). Inter-rater reliability showed a high Cohen’s kappa statistic (Cohen’s *k* = 0.828), with primary bias risks associated with participant sampling methods and sample size calculation. Longitudinal studies were assessed via the NOQAS (0–9 scale; *M* = 7.074, *SD* = 0.474), demonstrating high inter-rater agreement (Cohen’s *k* = 0.794). Major bias risks were linked to the objectivity of outcome assessment and follow-up completeness.

### Cross-sectional analysis

#### Effect size

The random effects model yielded an overall pooled prevalence of 13.20% (95% CI 12.06%–14.42%). The 95% prediction interval was wide (1.63%–58.29%), reflecting the extremely high between-study heterogeneity observed (*Q* (473) = 664,832, *P* < 0.001; *I*^2^ = 99.94%; *τ*^2^ = 1.279). This heterogeneity remained high (*I*^2^ = 99.56%) in a sensitivity analysis excluding studies with *n* > 10,000. Funnel plot asymmetry (Supplementary Materials 7) suggested potential publication bias, a finding statistically confirmed by both Egger’s test (*z* = −4.539, *P* < 0.0001) and the more appropriate Peters’ test for proportions (*z* = −4.720, *P* < 0.0001). However, a subsequent trim-and-fill analysis imputed zero missing studies (*k*₀ = 0), and the adjusted pooled prevalence was identical to the original estimate, suggesting the impact of this bias was negligible. Subgroup analysis showed no significant difference in prevalence between the pre-pandemic (12.52% [95% CI 8.46%–18.14%]) and post-pandemic periods (13.24% [95% CI 12.07%–14.50%]; *β* = 0.064, [95% CI −0.386–0.514]). Forest plots are available in Supplementary Materials 8.

#### Meta-regression analysis

The overall meta-regression model was significant (*QM* = 70.539, *P* < 0.0001) and explained 10.05% of the between-study variance. The analysis identified three significant moderators (Table 2). First, larger sample sizes were associated with lower prevalence rates (*β* = −0.232, *P* < 0.0001). Second, higher-quality studies reported lower prevalence (*β* = −0.278, *P* < 0.001). Third, studies on adults reported significantly lower prevalence compared with adolescents (*β* = −0.366, *P* < 0.05). Other potential moderators, such as gender and geographical area, were not significant (Table 2).

**Table 2.** Meta-regression results of the random effects model for the prevalence of SI

Predictor	Coefficient	*SE*	*z*	*P*	[95% CI]
Intercept	−0.131	0.599	−0.220	0.826	[−1.306, 1.042]
Time	0.072	0.046	1.569	0.116	[−0.018, 0.162]
Economic (developing country)	0.025	0.151	0.164	0.870	[−0.270, 0.320]
Economic (Unknown)	−1.749	1.193	−1.466	0.143	[−4.088, 0.590]
Area (Asia)	−0.157	0.277	−0.569	0.569	[−0.699, 0.385]
Area (Europe)	0.161	0.302	0.533	0.594	[−0.431, 0.753]
Area (Unknown)	1.932	1.111	1.739	0.082	[−0.245, 4.110]
Area (North America)	−0.263	0.315	−0.834	0.404	[−0.880, 0.354]
Area (Oceania)	0.054	0.572	0.095	0.924	[−1.067, 1.175]
Area (South America)	0.288	0.358	0.805	0.421	[−0.413, 0.989]
**Age (adult)**	**−0.366**	**0.148**	**−2.472**	**0.013**	**[−0.656, −0.076]**
Age (Mixed)	−0.465	0.250	−1.861	0.063	[−0.955, 0.025]
Age (Unknown)	−0.166	0.366	−0.455	0.649	[−0.884, 0.551]
Age (Old)	−0.734	0.415	−1.770	0.077	[−1.547, 0.079]
Gender (25%−50%)	−0.065	0.280	−0.230	0.818	[−0.614, 0.485]
Gender (50%−75%)	−0.005	0.258	−0.020	0.984	[−0.511, 0.500]
Gender (75%−100%)	−0.040	0.276	−0.144	0.885	[−0.580, 0.500]
Gender (Not reported)	−0.309	0.276	−1.119	0.263	[−0.851, 0.233]
**JBICAC**	**−0.278**	**0.078**	**−3.556**	**0.000**	**[−0.431, −0.125]**
Measurement (question)	−0.124	0.106	−1.167	0.243	[−0.331, 0.084]
***n***	**−0.233**	**0.050**	**−4.628**	**<.0001**	**[−0.331, −0.134]**

Note. The intercept represents the predicted effect size when all moderators in the model are equal to zero (or the reference category for categorical variables); JBICAC = Joanna Briggs Institute Critical Appraisal Checklist score; CI = confidence interval.

#### Sensitivity analysis: impact of measurement instrument

A sensitivity analysis on 127 studies found that the choice of measurement instrument was a significant source of heterogeneity (*QM* = 15.803, *P* < 0.0001). Specifically, a stratified meta-analysis showed that the pooled prevalence from studies using the C-SSRS was 28.11% (95% CI 21.08%–36.40%), which was significantly higher than the 13.90% (95% CI 11.58%–16.61%) from studies using the PHQ-9. The meta-regression confirmed this difference was statistically significant (*β* = 0.885, *P* < 0.0001).

Crucially, a subsequent analysis found no significant interaction between instrument type and the study period (pre- vs. post-pandemic, *P* = 0.447). This indicates that while baseline prevalence depends on the instrument, the temporal trend across the pandemic was consistent regardless of the tool used. This confirms that the primary conclusion regarding the change in prevalence over time is robust and not a methodological artefact.

#### Moderator interaction effects

Interaction analyses were conducted to explore if the temporal trend of SI differed across key subgroups (Table 3). The analysis revealed no significant interaction between time and economic development level (*P* = 0.241). Similarly, for the time × geographical area interaction, while the overall model was significant (*QM* = 31.125, *P* = 0.003), none of the specific interaction terms reached statistical significance. In summary, the exploratory analyses did not identify significant moderating effects of economic development level or geographical area on the temporal trend of SI prevalence.

**Table 3.** Results of the interaction analysis

Predictor	Coefficient	*SE*	*z*	*P*	[95% CI]
Time: Economic					
**Intercept**	**−2.197**	**0.205**	**−10.743**	**<.0001**	**[−2.598, − 1.796]**
Time	0.076	0.059	1.305	0.192	[−0.038, 0.191]
Economic (developing country)	0.546	0.360	1.516	0.129	[−0.160, 1.251]
Economic (unknown)	−2.024	4.104	−0.493	0.622	[−10.067, 6.020]
Time: Economic (developing country)	−0.116	0.099	−1.172	0.241	[−0.311, 0.078]
Time: Economic (unknown)	0.750	1.272	0.590	0.555	[−1.743, 3.244]
Time: Area					
Intercept	−1.894	0.987	−1.920	0.055	[−3.828, 0.039]
Time	0.025	0.276	0.089	0.929	[−0.516, 0.565]
Area (Asia)	0.157	1.024	0.153	0.878	[−1.850, 2.164]
Area (Europe)	−0.899	1.041	−0.864	0.388	[−2.939, 1.140]
Area (unknown)	−0.697	4.018	−0.173	0.862	[−8.571, 7.178]
Area (North America)	−0.043	1.026	−0.042	0.967	[−2.053, 1.968]
Area (Oceania)	0.935	4.135	0.226	0.821	[−7.170, 9.040]
Area (South America)	−0.856	1.794	−0.477	0.633	[−4.371, 2.659]
Time: Area (Asia)	−0.089	0.286	−0.311	0.756	[−0.649, 0.471]
Time: Area (Europe)	0.298	0.291	1.025	0.305	[−0.272, 0.868]
Time: Area (unknown)	0.395	1.252	0.315	0.753	[−2.060, 2.849]
Time: Area (North America)	−0.088	0.287	−0.305	0.760	[−0.650, 0.475]
Time: Area (Oceania)	−0.263	1.276	−0.206	0.837	[−2.764, 2.238]
Time: Area (South America)	0.372	0.525	0.709	0.478	[−0.657, 1.402]

#### National distribution of the prevalence of SI

**Figure 2.**
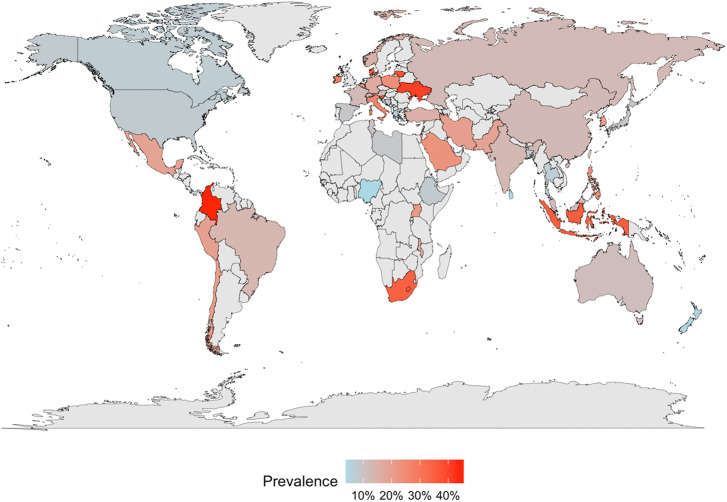
Post-pandemic suicidal ideation prevalence. Source: Grey represents countries with missing data.

Sample-weighted prevalence means were calculated and grouped by country to generate a global prevalence distribution map (Fig. 2). The map correlates sample-weighted mean SI prevalence with countries, using a colour gradient from light blue to red representing values from 0 to 1, with missing data indicated by light grey.

### Longitudinal analysis

Data analysis for longitudinal studies was divided into two parts: (1) pre-/post-pandemic (T1 vs. T2) changes in SI prevalence; (2) within-post-pandemic changes between early and late periods (T3 vs. T4).

#### Pre- versus post-pandemic comparison

**Figure 3.**
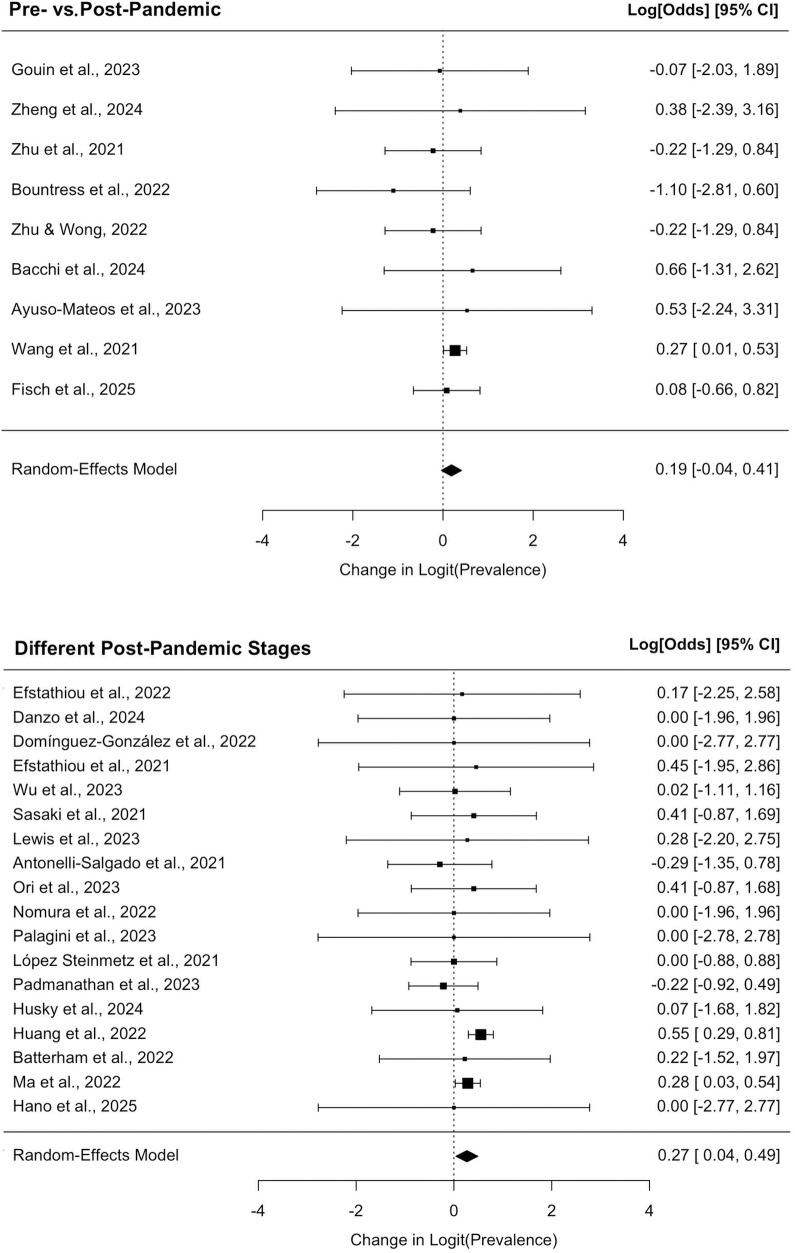
Forest plot. source: The effect size for each study is the log odds ratio (LOR), representing the change in prevalence between two time points. The top panel displays the comparison between the pre-pandemic and post-pandemic periods. The bottom panel displays the comparison between early and late post-pandemic periods. Diamonds represent the pooled LOR from random-effects models.

Based on 9 longitudinal studies, the pooled analysis using a random-effects model indicated no statistically significant change in SI prevalence from the pre-pandemic to the post-pandemic period (*β* = 0.188, [95% CI −0.037–0.412], *P* = 0.101). To illustrate the practical magnitude of this effect, it corresponds to an estimated increase in prevalence from a baseline of 10% to approximately 11.70%. There was negligible evidence of heterogeneity across studies (*I^2^* = 0.00%, *Q* (8) = 4.143, *P* = 0.844), as visually represented in the top panel of the forest plot (Fig. 3). Subgroup analyses stratified by geographic region, economic status, age and gender revealed no significant moderators of this effect.

#### Within-pandemic comparison

For the comparison between two distinct within-pandemic stages, the analysis of 18 studies revealed a statistically significant, albeit small, increase in prevalence over time (*β* = 0.265, [95% CI 0.040–0.490], *P* = 0.021). In terms of absolute change, this effect size represents an increase in prevalence from a hypothetical baseline of 10% to approximately 12.70%. Heterogeneity across studies was low and not statistically significant (*I^2^* = 13.24%, *Q* (17) = 7.922, *P* = 0.968). The corresponding forest plot is presented in the bottom panel of Fig. 3. Similar to the pre-post comparison, none of the examined moderators were found to significantly influence this change.

## Discussion

### Trends of SI prevalence rate

Cross-sectional analyses revealed pooled SI prevalences of 12.52% pre-pandemic and 13.24% post-pandemic, with no statistically significant difference observed. Longitudinal meta-analyses showed no significant changes between T1 (pre-pandemic) and T2 (post-pandemic), but a significant upward trend during T3–T4 (early to mid and late pandemic period). These findings are generally consistent with Tardeh *et al.* (2023) report of a 13% cross-sectional prevalence during the pandemic. However, they differ from Robinson *et al.* (2022) longitudinal meta-analysis, which observed an initial increase in mental health symptoms during the early pandemic (March–April), followed by a decline in the late phase (May–July). This discrepancy likely stems from the specific focus on SI and a longer observation period. Collectively, these results suggest that while global SI prevalence was stable initially, it increased significantly as the crisis progressed. This underscores the need for long-term mental health support systems that address chronic stressors like economic pressure and social isolation, beyond the initial crisis.

### Moderating variables influencing the SI prevalence rate

First, cross-sectional meta-regression showed significantly higher SI prevalence in adolescent groups compared to adults, which may be linked to adolescents’ neurodevelopmental characteristics that can weaken crisis response capabilities (Casey *et al.*, 2008). This aligns with findings from other meta-analyses, such as Bersia *et al.* (2022) report of a 17% SI prevalence among young people during COVID-19. This pronounced increase in SI among adolescents pinpoints them as a key vulnerable group. The evidence strongly supports a shift towards targeted, age-specific mental health interventions, such as integrating routine SI screening into future crisis preparedness plans and developing accessible, youth-focused support, including school-based programs and digital mental health platforms. Notably, the longitudinal meta-regression did not detect significant subgroup differences, possibly due to the smaller number of longitudinal studies available.

Second, study quality exerted a significant moderating effect, as high-quality studies reported significantly lower pooled prevalence. This highlights the critical role of methodological rigour in epidemiological estimates. While significant funnel plot asymmetry was detected (Egger’s test, *P* < 0.0001), a subsequent trim-and-fill analysis indicated that this asymmetry was not due to missing studies (*k*₀ = 0). This pattern strongly suggests that the observed asymmetry reflects true heterogeneity rather than classic publication bias; specifically, as smaller studies, which were often of lower methodological quality, tended to report systematically higher prevalence rates. Therefore, it is imperative for policymakers and researchers to prioritise data from large-scale, methodologically rigorous longitudinal studies, as high-bias studies may systematically overestimate SI prevalence. This need for rigour extends beyond study design to assessment frequency, as the low-frequency measurements common in the included studies inevitably miss short-term fluctuations. Future research should incorporate high-frequency methods, such as Ecological Momentary Assessment, to better understand the real-time dynamics of suicidal thoughts.

Furthermore, the choice of measurement instruments considerably influenced prevalence estimates. Studies using specialised tools like the C-SSRS (Posner *et al.*, 2011) identified significantly more cases than broader screening questions contained within instruments like the PHQ-9. This methodological divergence challenges the comparability of evidence and shows how the instrument itself can influence the scale of the perceived problem. While absolute prevalence was instrument-dependent, the interaction analysis showed that the change in prevalence over time was consistent across instrument types, supporting the robustness of the primary conclusion. This finding reveals a critical need for greater standardisation and transparency in measurement. For future research, this underscores two imperatives: researchers must clearly report the tools used, and the field of suicidality research should aim to build a consensus on best practices for the epidemiological surveillance of suicidality to enhance data harmonisation.

### Limitations and directions for future research

First, longitudinal data covered only 14 countries (predominantly Asia/Europe/Americas), limiting geographic diversity and potentially obscuring risks in low- and middle-income regions with fragile social safety nets. Future research should prioritise multi-time point longitudinal surveys in Africa/Southeast Asia to investigate vaccine accessibility, economic policies and SI dynamics. Second, excluding exposed-cohort studies sacrificed sample diversity for homogeneity, risking omission of key heterogeneity drivers (e.g., infection impacts). Cohort studies with structural equation modelling are needed to disentangle direct (infection) versus indirect (lockdowns) pandemic effects on SI trajectories. Third, despite the meta-regression identifying key moderators such as age and measurement tools, substantial residual heterogeneity (*I^2^* > 95%) remained in the cross-sectional analyses. This suggests that the pooled prevalence estimates should be interpreted as an average effect across a wide variety of contexts, rather than a single, universal figure. Unmeasured factors, such as specific public health policies, cultural norms and the timing of data collection relative to local pandemic waves, likely contributed to this variability. Future research should aim to collect and analyse these granular, context-specific variables to better explain the diverse impacts of the pandemic. Fourth, the finding that lower-quality studies reported higher SI prevalence underscores the potential for methodological biases, such as convenience sampling and the lack of control for confounders, to inflate estimates. This was particularly pertinent to the cross-sectional data. While the longitudinal analysis provided more robust insights into change over time, it was based on a smaller set of studies, which may still be susceptible to attrition bias. The pandemic-era constraints on research highlight a critical need for future crisis preparedness to include protocols for rapid, methodologically sound and standardised longitudinal data collection to minimise bias and provide more reliable evidence.

## Conclusions

This meta-analysis reveals a significant increase in the global prevalence of SI as the COVID-19 pandemic progressed, a trend most pronounced among adolescents. This finding identifies youth as a key vulnerable population requiring prioritised mental health support in the wake of global crises such as the COVID-19 pandemic. Equally important, the analysis demonstrates that reported prevalence is heavily moderated by methodological factors, including study quality, sample size and choice of measurement instrument. This highlights the risk of overestimation from smaller, lower-quality studies and underscores the necessity of methodological rigour in psychiatric epidemiology. Ultimately, this study not only confirms the indispensable role of longitudinal research in capturing the true dynamics of mental health during crises but also provides critical evidence for building more resilient and responsive public health systems. The findings strongly advocate for the establishment of robust mental health surveillance and rapid-response intervention networks to enhance societal psychological resilience in the face of future challenges.

## Supporting information

10.1017/S2045796025100358.sm001 Tao et al. supplementary material 1 Tao et al. supplementary material

10.1017/S2045796025100358.sm002 Tao et al. supplementary material 2 Tao et al. supplementary material

## Data Availability

The datasets generated and/or analysed during the current study are available from the corresponding author on reasonable request.

## Supplementary material

The supplementary material for this article can be found at https://doi.org/10.1017/S2045796025100358.
