# Supplementary material for: Temporal patterns of suicidal ideation prevalence during the COVID-19 pandemic: a systematic review and meta-analysis of cross-sectional and longitudinal studies
Source: Epidemiol Psychiatr Sci. 2025 Dec 19;34:e61. doi: 10.1017/S2045796025100358 (PMC12722189; doi:10.1017/S2045796025100358)
Supplement: Tao et al. supplementary material 1 — Tao et al. supplementary material [file S2045796025100358sup001.pdf]

Contents

Supplementary Materials 1 2

Supplementary Materials 2 3

Supplementary Materials 3 4

Supplementary Materials 4 6

Supplementary Materials 5 9

Supplementary Materials 6 17

Supplementary Materials 7 27

Supplementary Materials 8 28

Supplementary Materials 9 29

Supplementary Materials 1. Search strategies

	Search Strategy	Quantity	Supplementary	Search deadline
**Web of Science**	#1 TS=("suicidal ideation" OR "suicidal thoughts" OR "suicidal intent")<br>#2 TS=("COVID-19" OR covid19 OR "SARS-CoV-2" OR coronavirus* OR "corona virus*" OR "2019-nCoV" OR betacoronavirus* OR "beta coronavirus*" OR "novel coronavirus*")<br>#3 #1 AND #2	1498	151	2025.8.30
**PubMed**	#1 "suicidal ideation"[MeSH Terms]<br>#2 "suicidal ideation"[Title/Abstract] OR "suicidal thoughts"[Title/Abstract] OR "suicidal intent"[Title/Abstract]<br>#3 #1 OR #2<br>#4 "COVID-19"[MeSH Terms] OR "SARS-CoV-2"[MeSH Terms] OR "Coronaviridae Infections"[MeSH Terms]<br>#5 "COVID-19"[Title/Abstract] OR "covid19"[Title/Abstract] OR "SARS-CoV-2"[Title/Abstract] OR "coronavirus*"[Title/Abstract] OR "corona virus*"[Title/Abstract] OR "2019-nCoV"[Title/Abstract] OR "betacoronavirus*"[Title/Abstract]<br>#6 #4 OR #5<br>#7 #3 AND #6	835	57	2025.8.30
**ProQuest**	s1. su("suicidal ideation")<br>s2. ti,ab("suicidal ideation" OR "suicidal thoughts" OR "suicidal intent")<br>s3. s1 OR s2<br>s4. su("COVID-19" OR "Coronaviruses")<br>s5. ti,ab("COVID-19" OR covid19 OR "SARS-CoV-2" OR coronavirus* OR "corona virus*" OR "2019-nCoV" OR betacoronavirus*)<br>s6. s4 OR s5<br>s7. s3 AND s6	384	67	2025.8.30
**PsyINFO**	S1. DE "Suicidal Ideation" (Explode)<br>S2. TI ("suicidal ideation" OR "suicidal thought*" OR "suicidal intent") OR AB ("suicidal ideation" OR "suicidal thought*" OR "suicidal intent")<br>S3. S1 OR S2<br>S4. DE "COVID-19" (Explode)<br>S5. TI ("COVID-19" OR covid* OR "SARS-CoV-2" OR coronavirus* OR "corona virus*" OR "2019-nCoV" OR betacoronavirus*) OR AB ("COVID-19" OR covid* OR "SARS-CoV-2" OR coronavirus* OR "corona virus*" OR "2019-nCoV" OR betacoronavirus*)<br>S6. S4 OR S5<br>S7. S3 AND S6	350		2025.8.30

Supplementary Materials 2. Eligibility criteria

Studies were included if they met the following criteria:

(a) The research theme was related to the COVID-19 pandemic.

(b) SI was assessed as an outcome, with its prevalence explicitly reported or calculable from the data provided. The operational definition of an SI measurement included:

(i) Validated and Standardized Assessment Tools: Studies using established, psychometrically validated scales or specific items from such instruments. Examples include Item 9 of the Patient Health Questionnaire-9 (PHQ-9), relevant items from the Columbia-Suicide Severity Rating Scale (C-SSRS), and the suicide-related item from the Beck Depression Inventory-II (BDI-II).

(ii) Direct Unambiguous Questions: Studies employing one or more clear, unambiguous questions to ascertain the presence of SI (e.g., “Have you had thoughts of suicide in the past year?”). This approach was included to capture a wider range of epidemiological data collected during the pandemic.

(c) Both cross-sectional and longitudinal study designs were eligible. (d) The study was reported in English.

Studies were excluded if they:

(a) Were unrelated to the COVID-19 pandemic.

(b) Did not report SI prevalence, but only reported related constructs such as suicide risk, suicide attempts, or suicidal behavior.

(c) Were qualitative studies, intervention studies, literature reviews, meta-analyses, or animal studies.

Supplementary Materials 3. Coding standards

1. **Sample Size (N)**
   • N denotes the sample size used to calculate the prevalence of suicidal ideation (i.e., the denominator).
2. **Suicidal Ideation (SI)**
   • SI refers to the prevalence of suicidal ideation, typically classified as "Yes" or "No."
   • In studies reporting multiple prevalence, use the overall prevalence or the most recent time-point prevalence.
   • If different levels (mild, moderate, severe) of suicidal ideation are reported, all are considered as "Yes."
3. **Time of Measurement**
   • Time 1: the time point when the measurement was taken is broadly categorized as "pre-epidemic vs. post-epidemic," where pre-epidemic is coded as 1 and post-epidemic as 2, using March 2020 as the dividing point.
   • Time 2: further sub-categories are defined as follows:
      a. Pre – Pre-epidemic (up to December 2019); coded as 1
      b. Early – Early stage of the epidemic (December 2019 to March 2020); coded as 2
      c. Outbreak1 – Peak period 1 (March 2020 to December 2020); coded as 3
      d. Outbreak2 – Peak period 2 (January 2021 to December 2021); coded as 4
      e. Outbreak3 – Peak period 3 (January 2022 to December 2022); coded as 5
      f. Mitigation – Post-epidemic period (from January 2023 onwards); coded as 6
      g. Unidentifiable – coded as 7
4. **Geographic Area (Area)**
   • The region where the study was conducted, categorized by continent.
   • Major continents included: Asia, Europe, North America, South America, Africa, and Oceania.
5. **Economic Classification (Economic)**
   • Economic status is divided into:
      – Developed country
      – Developing country
6. **Age Groups (Age)**
   • Participants' ages are divided into the following categories:
      – Adolescents; coded as adolescent
      – Adults; coded as adult
      – Older adults; coded as old
      – All age groups; coded as mixed
      – Not reported; coded as unknown
   • The corresponding codes are: adolescent, adult, old, full, and none, respectively.
7. **Gender**
   • Gender is represented as the percentage of female participants.
   • The final coding based on percentage intervals is as follows:
      – 0–25%: coded as 1

– 25–50%: coded as 2
– 50–75%: coded as 3
– 75–100%: coded as 4
– Not reported: coded as 5

8. **Measurement method**
   – Validated scale: coded as 1
   – Clear and accurate questions: coded as 2

9. **Measurement Tools**
   – PHQ-9 coded as 0
   – C-SSRS coded as 1
   – others codes as 2

Supplementary Materials 4. QualityAssessment Tools

A. JBI Cross-Sectional Study Quality Assessment Tool (Total Score: 7 points)

Each of the following 7 items is scored 1 if the criteria are met, otherwise 0. The detailed guidelines are as follows:

1. Sampling Frame Suitability for the Target Population

   • Criterion: The study must clearly indicate its “geographic scope,” “time period,” and “population characteristics (e.g., age/gender/occupation, etc.).”

   • Scoring: Score 1 if all are clearly stated; otherwise, score 0.

2. Appropriateness of Participant Recruitment Methods

   • Criterion: Recruitment by stratified sampling, random sampling, or consecutive sampling is acceptable and yields 1 point.

   • Note: Studies using cluster sampling or convenience sampling that acknowledge their limitations and implement adjustment methods may receive 1 point.

   • Scoring: Studies that use cluster sampling or convenience sampling without proposed adjustments, or those recruiting via self-enrollment, paid recruitment, or solely through social media should receive 0.

3. Scientific Validity of Sample Size Calculation

   • Criterion: The study must specify how the sample size was calculated, or provide rationale for parameter selection (e.g., an expected prevalence supported by literature), or indicate that a power test (typically ≥80%) was conducted.

   • Scoring: Score 1 if any of these conditions are met; otherwise, score 0.

4. Adequacy in Describing Study Subjects and Settings

   • Criterion: The study should report demographic characteristics (e.g., age [presented as mean ± SD or median + IQR], gender distribution, key exposure factors) and environmental characteristics (e.g., specifying outpatient/inpatient divisions for tertiary hospitals, or urban/rural types for community studies).

   • Additionally, the reporting of the execution of exclusion criteria is required.

   • Scoring: Score 1 if these details are provided; otherwise, score 0.

5. Validity of Measurement for Study Variables

   • Criterion: Measurement instruments must be either a gold standard, a validated alternative, or a newly developed tool with some validation.

   • Scoring: Score 1 if one of these conditions is met; otherwise, score 0.

6. Standardization of Measurement Procedures

   • Criterion: Data collection methods should be consistent across all participants to avoid measurement bias, with detailed descriptions of the steps and tools used.

   • Scoring: Score 1 if the procedure is standardized as required; otherwise, score 0.

7. Appropriateness of Statistical Analysis Methods

   • Criterion: The statistical methods used must align with the study's objectives, and the results should be interpreted reasonably based on the data. Details of the statistical analysis (including the software and specific methods used) must be provided.

   • Note: Drawing causal inferences from cross-sectional data will result in a score of 0.

• Scoring: Score 1 if these conditions are satisfied; otherwise, score 0.

B. NOQAS Longitudinal Study Quality Assessment Tool (Total Score: 9 points)

Each item is scored as described below:

1. Study Population Definition
   • Questions:
   a. Does the study clearly describe the inclusion/exclusion criteria (e.g., age, disease status, baseline characteristics)?
   b. Are the inclusion criteria operationalized (e.g., with reference to diagnostic tools or scales)?
   • Scoring: If both questions are answered "Yes," score 1; if either is "No," score 0.
2. Representativeness of the Sample
   • Questions:
   a. Is the source of the study sample clearly described (e.g., population-based random sampling, consecutive case enrollment)?
   b. Does the study explain the relationship between the sample and the target population (e.g., a specific group from a certain region or institution)?
   • Scoring: If at least one question is "Yes," score 1; if both are "No," score 0.
3. Completeness of Baseline Data
   • Questions:
   a. Does the study report baseline characteristics (e.g., demographics, disease status, main measurement indicators)?
   b. Does the baseline data include key variables (e.g., potential confounding factors affecting the outcomes)?
   • Scoring: If both questions are answered "Yes," score 1; if either is "No," score 0.
4. Clarity of the Time Frame
   • Questions:
   a. Does the study clearly report the follow-up time points (e.g., baseline, 6 months, 1 year, etc.) along with the total follow-up duration?
   b. Is the timing of the measurements appropriately set to cover the critical window for outcome occurrence?
   • Scoring: If both questions are "Yes," score 1; if either is "No," score 0.
5. Consistency in Measurement
   • Questions:
   a. Are the repeated measurement tools validated or standardized (e.g., citation of standardized scales, instrument calibration records)?
   b. Are the measurement methods consistent across different time points?
   • Scoring: If both questions are "Yes," score 1; if either is "No," score 0.
6. Objectiveness of Outcome Assessment
   • Questions:
   a. Are outcomes measured using objective indicators (e.g., laboratory tests, imaging, hard endpoint events)?
   b. Is blinded assessment employed (e.g., outcome assessors are unaware of the

measurement time points)?

• Scoring: If at least one question is “Yes,” score 1; if both are “No,” score 0.

7. Appropriateness of Statistical Analysis Methods

• Questions:

a. Does the study use analysis methods suitable for longitudinal data (e.g., mixed-effects models, repeated measures ANOVA)?

b. Does the study report the significance of time trends (e.g., slope estimates, P-values)?

• Scoring: If both questions are “Yes,” score 1; if either is “No,” score 0.

8. Control of Confounding Factors

• Criterion: Does the study control for at least two time-fixed confounding factors (e.g., baseline age, gender, GDP changes)?

• Scoring: Score 1 if “Yes”; otherwise, score 0.

9. Completeness of Follow-up

• Questions:

a. Does the study report the number of participants lost to follow-up?

b. Is the loss-to-follow-up rate below 20%?

c. Does the study analyze the reasons for loss to follow-up or assess potential bias through sensitivity analyses (e.g., comparing baseline characteristics between those lost and those completing follow-up)?

• Scoring: If all three questions are answered “Yes,” score 1; if anyone is “No,” score 0.

Supplementary Materials 5

1. Supplementary Materials for Screen Process

Screening	Total	Exclude	Reason
Title and abstract	2013	1387	The main content does not conform to the theme of this study.
Full text	626	245	126 articles were excluded due to missing data;<br>25 articles were excluded due to database overlap;<br>48 articles were excluded because their study design did not meet the inclusion criteria.
Quality assessment	381		

2. Excluded Studies Due to Database Duplicates

Title	Database
A Comparison of Changes in Health Behavior, Obesity, and Mental Health of Korean Adolescents Before and During the COVID-19 Pandemic: Online Cross-Sectional Study	2019 and 2020 Korea Youth Risk Behavior Web-based Survey
Association of the COVID-19 lockdown with health risk behaviors in South Korean adolescents	2019 and 2020 Korea Youth Risk Behavior Web-based Survey
Changes in Daily life due to the COVID-19 Pandemic and Mental Health Status in Korean Adolescents	2021 Korea Youth Risk Behavior Web-based Survey
Changes in Daily Life, Physical Activity, GAD, Depression, and Personal Hygiene of Adolescents in South Korea Due to the COVID-19	2020 and 2021 Youth Health Behavior Survey
**Changes in mental health of Korean adolescents before and during the COVID-19 pandemic: a special report using the Korea Youth Risk Behavior Survey**	**2018–2021 Korea Youth Risk Behavior Survey**
Changes in Physical Activity and Depression among Korean Adolescents Due to COVID-19: Using Data from the 17th (2021) Korea Youth Risk Behavior Survey	2021 Korea Youth Risk Behavior Survey
Comparisons of Characteristics Between Psychological Support Hotline Callers With and Without COVID-19 Related Psychological Problems in China	2019 and 2020 Korea Youth Risk Behavior Web-based Survey
Effect of Subjective Economic Status During the COVID-19 Pandemic on Depressive Symptoms and Suicidal Ideation Among South Korean Adolescents	2020 Korea Youth Risk Behavior Web-based Survey
Family economic hardship and adolescent mental health during the COVID-19 pandemic	2020 Korea Youth Risk Behavior Web-based Survey
Impact of emotional state and suicidal intentions on suicide attempts among Korean adolescents with household financial difficulties following the outbreak of COVID-19: A cross-sectional study	2020 Korea Youth Risk Behavior Web-based Survey
Mental Health of Adolescents and Subjective Economic Deterioration Caused by COVID-19 in Korea	2020 Korea Youth Risk Behavior Web-based Survey
Mental health of South Korean adolescents in the COVID-19 Era: Web-based survey focused on suicide-related factors	2020 Korea Youth Risk Behavior Web-based Survey
National trends in suicide-related behaviors among youths between 2005-2020, including COVID-19: a Korean representative survey of one million adolescents	2005–2020 Korea Youth Risk Behavior Web-based Survey
The Impact of Household Economic Deterioration Caused by the COVID-19 Pandemic and Socioeconomic Status on Suicidal Behaviors in Adolescents: A Cross-sectional Study Using 2020 Korea Youth Risk Behavior Web-based Survey Data	2020 Korea Youth Risk Behavior Web-based Survey

Since multiple studies have collectively utilized the "Korea Youth Risk Behavior Web-based Survey" database, only the study **presented in bold font** within the table was retained. This decision was made because the time scope of this study is broader, covering the time points of the other studies included.

3. Table of Detailed Information for All Included Studies

Cross-sectional studies

Citation	n	SI	Time1	Time2	Area	Country	Economic	Age	Gender	JBI	Measurement	Tools
Delgadillo et al., 2023a	1005	0.1730	2	3	Europe	Austria	developed country	mixed	3	6	1	0
Delgadillo et al., 2023b	1006	0.3170	2	3	Europe	United Kingdom	developed country	mixed	3	6	1	0
Zhang et al., 2024	208	0.0673	2	6	Asia	China	developing country	adult	5	7	2	2
Sanchez Merino et al., 2024	450	0.3910	2	5	Europe	Spain	developed country	adolescent	4	4	1	2
Yamashita et al., 2024	200	0.2300	2	4	Asia	Japan	developed country	adult	2	7	1	0
Goodwill and Ajibewa, 2024	487	0.0966	2	5	North America	United States	developed country	adult	3	6	2	2
Monteith et al., 2023	567	0.0505	2	3	North America	United States	developed country	old	1	6	1	2
Gonzalez Mendez et al., 2022	1263	0.0830	2	3	Asia	China	developing country	adult	4	5	1	0
Peng et al., 2022	6183	0.2200	2	4	Asia	China	developing country	unknown	5	5	2	2
C Park et al., 2023a	394	0.1594	2	2	North America	United States	developed country	adult	3	5	1	0
C Park et al., 2023b	222	0.1604	2	4	North America	United States	developed country	adult	4	5	1	0
Rus Prelog et al., 2023	1790	0.0970	2	4	Europe	Italy	developed country	adult	5	5	1	2
M Wang et al., 2021	460	0.2326	2	2	Asia	China	developing country	mixed	3	6	1	0
Moderato et al., 2021	858	0.0300	2	3	Europe	Italy	developed country	adult	4	5	2	2
Yau and Nager, 2021	1261	0.0100	2	3	North America	United States	developed country	adolescent	5	5	2	2
Rabasco et al., 2021	216	0.4300	2	3	North America	United States	developed country	adult	4	5	1	2
Höller and Forkmann, 2022	1311	0.2170	2	4	Europe	Germany	developed country	adult	4	5	1	2
Gao et al., 2023	949	0.1610	2	5	Asia	China	developing country	adult	3	5	1	0
Wu et al., 2022	686	0.0520	2	3	Asia	China	developing country	adolescent	2	5	1	2
Essadek et al., 2022a	8004	0.1420	2	3	Europe	France	developed country	adult	3	7	1	0
Essadek et al., 2022b	3611	0.1490	2	3	Europe	France	developed country	adult	3	7	1	0
Essadek et al., 2022c	7535	0.2090	2	3	Europe	France	developed country	adult	3	7	1	0
Bressington et al., 2020	11072	0.2250	2	3	Asia	China	developing country	adult	4	5	2	2
Yamazaki et al., 2022	98	0.2040	2	4	Asia	Japan	developed country	adult	2	6	2	2
Valladares-Garrido et al., 2024	370	0.3430	2	3	South America	Peru	developing country	adult	3	5	1	0
Wei et al., 2023	1338	0.0972	2	5	Asia	China	developing country	adult	3	5	1	0
El Frenn et al., 2023	402	0.1820	2	4	Asia	Lebanon	developing country	adult	3	5	1	1
Kabir et al., 2023	1264	0.1326	2	4	Asia	Bangladesh	developing country	adult	3	5	1	2
Liu et al., 2021	5175	0.0300	2	3	Asia	China	developing country	unknown	5	5	2	2
Rogers et al., 2023	5528	0.4680	2	3	Europe	none	developing country	adult	4	5	1	1
Daly et al., 2021	3000	0.0590	2	3	North America	Canada	developed country	adult	3	6	2	2
Narita et al., 2023	1077	0.2900	2	4	North America	United States	developed country	adult	3	5	1	1
Stickley et al., 2022	1452	0.1170	2	4	Asia	Japan	developed country	adult	3	6	2	2
Woolverton et al., 2024	1956	0.2860	2	5	North America	United States	developed country	adult	3	6	2	2
Carlos et al., 2023	134	0.1760	1	1	North America	United States	developed country	adult	3	5	1	2
Kleinhendler-Lustig et al., 2023a	160	0.1500	1	1	Asia	Israel	developed country	adult	3	5	2	2
Kleinhendler-Lustig et al., 2023b	86	0.1050	2	2	Asia	Israel	developed country	adult	3	5	2	2
Ernst et al., 2022a	144	0.1530	2	3	Europe	Germany	developed country	adult	3	6	1	0
Ernst et al., 2022b	2359	0.0900	2	3	Europe	Germany	developed country	adult	3	6	2	2
Raviv et al., 2021a	32217	0.0050	2	3	North America	United States	developed country	adolescent	5	5	2	2
Raviv et al., 2021b	32217	0.0060	2	2	North America	United States	developed country	adolescent	5	5	2	2
Workneh et al., 2023a	300	0.0300	2	3	Africa	Ethiopia	developing country	adult	3	6	1	0
Workneh et al., 2023b	277	0.0550	2	4	Africa	Ethiopia	developing country	adult	3	6	1	0
Rogers et al., 2024a	127	0.4020	2	3	South America	Brazil	developing country	adult	4	5	1	1
Rogers et al., 2024b	65	0.6000	2	3	North America	Canada	developed country	adult	3	5	1	1
Rogers et al., 2024c	532	0.4360	2	3	Europe	Germany	developed country	adult	2	5	1	1
Rogers et al., 2024d	302	0.2750	2	3	Asia	India	developing country	adult	2	5	1	1
Rogers et al., 2024e	195	0.3590	2	3	Europe	Israel	developed country	adult	2	5	1	1
Rogers et al., 2024f	309	0.6930	2	3	Europe	Poland	developing country	adult	3	5	1	1
Rogers et al., 2024g	561	0.5190	2	3	Europe	Russia	developing country	adult	3	5	1	1
Rogers et al., 2024h	1043	0.1800	2	3	Asia	South Korea	developed country	adult	1	5	1	1
Rogers et al., 2024i	434	0.3540	2	3	Europe	Turkey	developing country	adult	2	5	1	1
Rogers et al., 2024j	1970	0.6450	2	3	North America	United States	developed country	adult	3	5	1	1
Kim et al., 2022a	54948	0.1090	2	3	Asia	South Korea	developed country	adolescent	2	6	2	2
Kim et al., 2022b	54848	0.1270	2	3	Asia	South Korea	developed country	adolescent	2	6	2	2
Nigatu et al., 2023	3003	0.0720	2	3	North America	Canada	developed country	adult	3	6	2	2
Spiller et al., 2023	1232	0.1280	2	2	Europe	United States	developed country	old	5	6	2	2
B Park et al., 2023a	61018	0.0910	1	1	Asia	South Korea	developed country	adolescent	1	6	2	2
B Park et al., 2023b	56325	0.0880	2	2	Asia	South Korea	developed country	adolescent	1	6	2	2
B Park et al., 2023c	56874	0.1730	1	1	Asia	South Korea	developed country	adolescent	4	6	2	2
B Park et al., 2023d	52922	0.1500	2	2	Asia	South Korea	developed country	adolescent	4	6	2	2
Sivertsen et al., 2022a	21405	0.1570	2	4	Europe	Norway	developed country	adult	1	6	1	2
Sivertsen et al., 2022b	40807	0.1470	2	4	Europe	Norway	developed country	adult	4	6	1	2
Vidal et al., 2024	270153	0.7000	2	3	North America	United States	developed country	adolescent	3	5	1	0
Rappaport et al., 2024	8652	0.2190	2	3	North America	United States	developed country	adolescent	5	6	2	2
Lee et al., 2024	99055	0.0916	2	3	Asia	South Korea	developed country	adult	3	6	1	2
Kim et al., 2021a	48443	0.1260	1	1	Asia	South Korea	developed country	adolescent	2	5	2	2
Kim et al., 2021b	44216	0.1010	2	3	Asia	South Korea	developed country	adolescent	2	5	2	2
Escobar-Agreda et al., 2023	249	0.0680	2	3	South America	Peru	developing country	old	3	6	2	2
Mailloux et al., 2024	2398	0.2220	2	4	North America	Canada	developed country	adult	1	5	2	2
Molina et al., 2023	297	0.1684	2	4	Europe	Spain	developed country	adult	3	6	1	2
Macalli et al., 2022	1913	0.1270	2	3	Europe	France	developed country	adult	4	5	2	2
Kundu et al., 2022	1414	0.6100	2	7	North America	Canada	developed country	adult	5	5	2	2
Sharif et al., 2020a	375	0.0500	2	3	unknown	unknown	unknown	adult	5	5	1	2
Sharif et al., 2020b	47684	0.0220	2	3	North America	United States	developed country	adolescent	2	5	1	0
Bantjes et al., 2023a	504	0.6000	2	3	Africa	South Africa	developing country	adult	5	5	1	1
Bantjes et al., 2023b	448	0.6200	2	3	Africa	South Africa	developing country	adult	5	5	1	1
Blay Benzaken et al., 2023a	158	0.3090	1	1	Asia	Israel	developed country	adult	2	5	1	0
Blay Benzaken et al., 2023b	66	0.4090	2	3	Asia	Israel	developed country	adult	3	5	1	0
J Liu et al., 2023	4692	0.2100	2	4	North America	United States	developed country	adolescent	2	6	1	2
Osiogo et al., 2021	6041	0.1440	2	3	North America	Canada	developed country	adult	4	6	2	2
Iftene et al., 2022	508	0.1950	2	3	North America	Canada	developed country	adult	3	4	1	2
Han et al., 2023a	309	0.4010	2	4	Asia	South Korea	developed country	adult	3	6	1	0
Han et al., 2023b	1055	0.1910	2	4	Asia	South Korea	developed country	adult	2	6	2	2
Li et al., 2020	1970	0.1080	2	3	Asia	China	developing country	adult	3	5	2	2
Burke et al., 2022	121	0.2310	2	3	North America	United States	developed country	adolescent	3	6	1	2
Luethy et al., 2023	112	0.1400	2	5	North America	United States	developed country	adult	4	5	1	2
Choi et al., 2021	31720	0.0833	1	1	Asia	South Korea	developed country	adult	2	5	2	2
Wen et al., 2024	4768	0.0540	2	3	Asia	China	developing country	adult	3	6	2	2
Sahimi et al., 2021	171	0.1110	2	3	Asia	Malaysia	developing country	adult	3	5	1	0
MacDonald et al., 2022a	3113	0.0160	2	7	North America	United States	developed country	adolescent	2	6	1	0
MacDonald et al., 2022b	496	0.0140	2	3	North America	United States	developed country	adolescent	2	6	1	0
M-Y Chen et al., 2024	10647	0.0617	2	6	Asia	China	developing country	adult	5	5	2	2
Zhou et al., 2021	11133	0.0730	2	3	Asia	China	developing country	adult	3	5	1	0
Ali et al., 2022a	731	0.1630	2	4	Asia	Bangladesh	developing country	adult	2	6	2	2
Ali et al., 2022b	587	0.1400	2	3	Europe	Slovenia	developed country	adult	4	6	1	2

Ali et al., 2022c	511	0.2840	2	5	Europe	Slovenia	developed country	adult	4	6	1	2
Drucker et al., 2023	104	0.2800	2	3	Asia	Israel	developed country	adult	4	4	1	2
Villanueva-Blasco et al., 2024	3780	0.0510	2	3	Europe	Spain	developed country	adult	3	5	1	0
S Al-Humadi et al., 2021	225	0.0660	2	3	North America	United States	developed country	adult	3	5	1	0
Macalli et al., 2025a	4463	0.2110	2	7	Europe	France	developed country	adult	4	5	2	2
Macalli et al., 2025b	1768	0.2930	2	5	Europe	France	developed country	adult	4	5	2	2
Brady, Fenton, et al., 2023	377	0.1300	2	4	Europe	Ireland	developed country	adult	4	5	2	2
Joshi et al., 2022a	125	0.0600	2	4	Europe	United Kingdom	developed country	adult	3	4	2	2
Joshi et al., 2022b	1415	0.1630	2	3	North America	United States	developed country	adult	2	4	1	0
Bonello et al., 2021a	401	0.1180	1	1	Europe	Malta	developed country	adult	5	5	2	2
Bonello et al., 2021b	350	0.2320	2	3	Europe	Malta	developed country	adult	5	5	2	2
Deng et al., 2023a	7684	0.1630	2	5	Asia	China	developing country	adult	3	5	2	2
Deng et al., 2023b	14921	0.3010	2	5	Asia	China	developing country	adult	3	5	2	2
Richardson et al., 2022a	469	0.2150	2	2	North America	Canada	developed country	adult	5	5	2	2
Richardson et al., 2022b	214	0.0610	2	2	North America	Canada	developed country	adult	5	5	2	2
Richardson et al., 2022c	1519	0.0430	2	2	North America	Canada	developed country	adult	5	5	2	2
Richardson et al., 2022d	807	0.0200	2	2	North America	Canada	developed country	adult	5	5	2	2
Mallhi et al., 2022	1074	0.3200	2	3	Asia	Saudi Arabia	developing country	adult	3	5	1	0
Wathelet, Vincent, et al., 2022a	68891	0.1140	2	3	Europe	France	developed country	adult	3	5	2	2
Wathelet, Vincent, et al., 2022b	22540	0.1320	2	3	Europe	France	developed country	adult	3	5	2	2
Gadermann et al., 2021a	618	0.0830	2	3	North America	Canada	developed country	adult	3	6	2	2
Gadermann et al., 2021b	2382	0.0520	2	7	North America	Canada	developed country	adult	5	6	2	2
Mzumara, 2024	620	0.1630	2	7	Africa	Malawi	developing country	adult	5	5	2	2
Hamm et al., 2020	73	0.0700	2	3	North America	United States	developed country	old	3	6	1	0
Pouradeli et al., 2024	202	0.4650	2	7	Europe	Iran	developing country	adult	5	6	2	2
Benavides Morales and López Peláez, 2022	33	0.4540	2	3	South America	Colombia	developing country	adult	3	4	2	2
Wathelet et al., 2020	69054	0.1140	2	3	Europe	France	developed country	adult	3	5	2	2
Lizhi et al., 2021	18521	0.0280	2	3	Asia	China	developing country	adult	4	5	1	0
Pelissier et al., 2021	740	0.1470	2	4	Europe	France	developed country	adult	3	5	2	2
Saeed et al., 2024	353	0.3430	2	5	Asia	Iran	developing country	adult	4	6	1	2
Crisol-Deza et al., 2023	1238	0.1790	2	3	Africa	Peru	developing country	adult	3	5	1	0
Trettel et al., 2022	4203	0.1920	2	3	South America	Brazil	developing country	adult	3	7	1	2
Park and Lee, 2022	784	0.0920	2	3	Asia	South Korea	developed country	adolescent	3	7	2	2
Taniguchi et al., 2022a	990	0.1500	2	3	Asia	Japan	developed country	adult	5	7	2	2
Taniguchi et al., 2022b	1043	0.0520	2	3	Asia	Japan	developed country	adult	5	7	2	2
Taniguchi et al., 2022c	21389	0.0360	2	3	Asia	Japan	developed country	adult	5	7	2	2
Meller et al., 2022	2152	0.0380	2	3	South America	Brazil	developing country	adult	3	7	1	0
Mohd Fadhli et al., 2022	1290	0.1190	2	4	Asia	Malaysia	developing country	adolescent	3	7	2	2
Shaygan et al., 2024	703	0.2800	2	3	Asia	Iran	developing country	adolescent	3	7	1	2
Zhu et al., 2022	5175	0.0300	2	3	Asia	China	developing country	adolescent	2	6	2	2
Cheung et al., 2021a	717	0.2150	2	3	North America	United States	developed country	adult	5	5	1	0
Cheung et al., 2021b	508	0.1420	2	3	North America	Canada	developed country	adult	5	5	2	2
Cheung et al., 2021c	845	0.0760	2	3	Europe	United Kingdom	developed country	adult	5	5	2	2
Cheung et al., 2021d	8376	0.0760	2	3	Africa	Brazil	developing country	adult	5	5	2	2
Cheung et al., 2021e	454	0.2490	2	3	Asia	Philippines	developing country	adult	5	5	2	2
Cheung et al., 2021f	658	0.1030	2	3	Asia	South Korea	developed country	adult	5	5	2	2
Cheung et al., 2021g	12509	0.2134	2	3	Asia	China	developing country	adult	5	5	2	2
Cheung et al., 2021h	782	0.2550	2	3	Asia	Turkey	developed country	adult	5	5	2	2
Ali et al., 2023	214	0.1670	2	5	Asia	Saudi Arabia	developing country	adult	5	5	1	2
Young et al., 2021	1685	0.0500	2	3	North America	United States	developed country	adult	4	5	1	0
Prado et al., 2023	2437	0.3320	2	5	South America	Brazil	developing country	adult	3	5	1	0
Mucci et al., 2022a	115	0.2070	1	1	Europe	Italy	developed country	adolescent	2	6	1	1
Mucci et al., 2022b	65	0.1540	2	3	Europe	Italy	developed country	adolescent	2	6	1	1
Mucci et al., 2022c	241	0.4100	2	3	Europe	Italy	developed country	adolescent	3	6	1	1
Hong et al., 2021	4692	0.0650	2	3	Asia	China	developing country	adult	4	5	1	0
Molina et al., 2024	870	0.0790	2	3	Europe	Spain	developed country	adult	4	5	1	1
Brasso et al., 2023a	154	0.2730	2	7	Europe	Italy	developing country	adult	2	6	2	2
Brasso et al., 2023b	160	0.4190	2	4	Europe	Italy	developing country	adult	2	6	2	2
Elhadi et al., 2022	31557	0.0760	2	3	South America	Libya	developing country	adult	3	4	2	2
Erjavac et al., 2023	573	0.4500	2	3	North America	United States	developed country	adolescent	3	4	2	2
Jeong et al., 2023a	2417	0.0120	2	3	Asia	South Korea	developed country	adult	1	6	1	0
Jeong et al., 2023b	2932	0.0127	2	3	Asia	South Korea	developed country	adult	4	6	1	0
McLoughlin et al., 2022a	36	0.4380	2	3	Europe	Ireland	developed country	adult	3	6	1	2
McLoughlin et al., 2022b	6592	0.0420	2	4	North America	Canada	developed country	adult	3	6	2	2
Sato et al., 2004	558	0.1020	2	5	Asia	Japan	developed country	adult	3	5	1	0
Thomson et al., 2021a	618	0.0840	2	3	North America	Canada	developed country	adult	3	6	2	2
Thomson et al., 2021b	804	0.0830	2	3	North America	Canada	developed country	adult	3	6	2	2
Thomson et al., 2021c	602	0.0790	2	4	North America	Canada	developed country	adult	3	6	2	2
Reyniers et al., 2022a	965	0.2030	2	3	Europe	Belgium	developed country	unknown	1	6	1	0
Reyniers et al., 2022b	965	0.2100	2	3	Europe	Belgium	developed country	unknown	1	6	2	2
Reinke et al., 2023a	330080	0.0009	1	1	North America	United States	developed country	adult	5	6	2	2
Reinke et al., 2023b	160320	0.0009	2	3	North America	United States	developed country	adult	5	6	2	2
Goodyear et al., 2021	502	0.1690	2	7	North America	Canada	developed country	adult	5	6	2	2
Peper-Nascimento et al., 2024	2259	0.1093	2	4	South America	Brazil	developing country	adult	3	5	1	1
Gainza Perez et al., 2022	159	0.2770	2	3	North America	United States	developed country	adult	2	5	1	2
Mushtaque et al., 2024	241	0.2240	2	3	Asia	Pakistan	developing country	adolescent	3	5	1	2
Li et al., 2024	5380	0.1280	2	3	Asia	China	developing country	adolescent	2	6	2	2
Shewangzaw Engda et al., 2022	700	0.1100	2	3	Africa	Ethiopia	developing country	adult	4	7	2	2
Wang et al., 2020	2031	0.1804	2	3	North America	United States	developed country	adult	3	5	1	0
Lee and Nam, 2023	2000	0.2030	2	4	Asia	South Korea	developed country	adult	2	6	2	2
John et al., 2021a	2221	0.0770	2	3	Europe	United Kingdom	developed country	adult	5	6	2	2
John et al., 2021b	4246	0.0940	2	3	Europe	United Kingdom	developed country	adult	5	6	2	2
John et al., 2021c	4382	0.1000	2	3	Europe	United Kingdom	developed country	adult	5	6	2	2
Ahuvia et al., n.d.	415	0.2700	2	3	North America	United States	developed country	adult	4	5	1	0
Howard et al., 2022a	323	0.1920	2	5	North America	United States	developed country	adult	4	5	1	0
Howard et al., 2022b	270	0.0070	2	5	North America	United States	developed country	adult	4	5	2	2
Matsushima et al., 2023a	153	0.2876	2	3	Asia	Japan	developed country	adult	4	6	2	2
Matsushima et al., 2023b	615	0.1675	2	3	Asia	Japan	developed country	adult	4	6	2	2
Jupina et al., 2022	960	0.0720	2	3	North America	United States	developed country	adult	3	5	2	2
Žilinskas et al., 2021	1001	0.3750	2	4	Europe	Lithuania	developing country	adult	4	6	2	2
Leroy et al., 2021a	4193	0.0910	2	3	Europe	France	developed country	adult	5	5	2	2
Leroy et al., 2021b	5431	0.1150	2	3	Europe	France	developed country	adult	5	6	2	2
Leroy et al., 2021c	59404	0.1160	2	3	Europe	France	developed country	adult	5	6	2	2
Ramirez et al., 2022	5037	0.2050	2	3	South America	Chile	developing country	adult	3	7	2	2
Fountoulakis et al., 2022	507	0.0726	2	3	Europe	Greece	developed country	adult	4	5	1	2
Kholmogorova et al., 2021	110	0.1100	2	3	Europe	Russia	developing country	adult	3	5	1	2
Sljivo et al., 2022	827	0.0860	2	3	Europe	Bosnia and Herzegovina	developing country	adult	4	6	2	2
Bustos Villarroel et al., 2024	125	0.2990	2	4	South America	Chile	developing country	adolescent	3	5	1	2

Rolland et al., 2022	11754	0.1900	2	4	Europe	France	developed country	adult	3	6	2	2
Dale et al., 2023a	1257	0.3330	2	4	Europe	Austria	developed country	adolescent	4	5	1	0
Dale et al., 2023b	1257	0.4570	2	4	Europe	Austria	developed country	adolescent	4	5	1	0
Arsandaux et al., 2021	1919	0.1010	2	3	Europe	France	developed country	adult	4	5	2	2
Rutkowska et al., 2022	753	0.1800	2	4	Europe	Poland	developed country	adult	3	5	1	2
Killgore et al., 2021	1013	0.1760	2	3	North America	United States	developed country	adult	3	6	1	0
Chodkiewicz et al., 2021	618	0.2362	2	3	Europe	Poland	developed country	adult	4	5	2	2
Troya et al., 2022	1983	0.0380	2	3	Europe	Ireland	developed country	adult	3	7	1	0
Chakrabarti, 2021	590	0.0500	2	3	Asia	India	developing country	adult	2	5	2	2
Schad et al., 2022a	309	0.1900	2	3	North America	United States	developed country	adult	5	5	2	2
Schad et al., 2022b	622	0.0600	2	3	North America	United States	developed country	adult	5	5	2	2
Frajerman et al., 2022	1925	0.1900	2	4	Europe	France	developed country	adult	3	5	2	2
Frajerman, 2023	11754	0.1900	2	4	Europe	france	developed country	adult	5	7	2	2
Dooley et al., 2024	4404	0.4199	2	4	Europe	Ireland	developed country	adolescent	2	5	2	2
Oliveira et al., 2022	890	0.0740	2	3	South America	Brazil	developing country	adult	4	5	1	2
Beymer et al., 2023a	21293	0.1620	2	3	North America	United States	developed country	adult	1	5	1	0
Beymer et al., 2023b	10861	0.1710	2	4	North America	United States	developed country	adult	1	5	1	0
Niederkrotenthaler et al., 2022	12029	0.0700	2	3	Europe	Austria	developed country	adult	3	6	1	2
An et al., 2022a	4940	0.7870	1	1	Asia	China	developing country	adult	3	5	2	2
An et al., 2022b	5550	0.7340	2	3	Asia	China	developing country	adult	3	5	2	2
Genis-Mendoza et al., 2021	1011	0.0446	2	3	North America	Mexico	developing country	adult	3	5	2	2
Tsiouris et al., 2023a	4351	0.1600	1	1	Europe	Germany	developed country	adult	3	5	1	0
Tsiouris et al., 2023b	3066	0.1630	2	3	Europe	Germany	developed country	adult	3	5	1	0
Tsiouris et al., 2023	1438	0.2140	2	4	Europe	Germany	developed country	adult	3	5	1	0
Jilowa et al., 2022	207	0.0290	2	3	Asia	India	developing country	adult	1	5	2	2
Korkmaz et al., 2022	573	0.1920	2	4	Europe	Turkey	developing country	adult	3	5	1	0
Stonehouse et al., 2023	295	0.1200	2	3	Africa	South Africa	developing country	adult	1	5	1	2
Wathelet, Horn, et al., 2022a	68106	0.1140	2	3	Europe	France	developed country	adult	5	6	2	2
Wathelet, Horn, et al., 2022b	22205	0.1320	2	3	Europe	France	developed country	adult	5	6	2	2
Wathelet, Horn, et al., 2022c	44898	0.1380	2	4	Europe	France	developed country	adult	5	6	2	2
Kohls et al., 2021	3382	0.1450	2	3	Europe	Germany	developed country	adult	3	5	1	0
Czeisler et al., 2021	1157	0.1690	2	3	Oceania	Australia	developed country	adult	3	7	2	2
Landi et al., 2023	652	0.1530	2	3	Europe	Italy	developed country	adult	4	5	1	0
He et al., 2023a	3006	0.1090	2	3	Asia	China	developing country	adult	3	5	1	0
He et al., 2023b	3465	0.0760	2	4	Asia	China	developing country	adult	3	5	2	2
He et al., 2023c	3917	0.0840	2	5	Asia	China	developing country	adult	3	5	2	2
Dogan-Sander et al., 2021	5642	0.1650	2	4	Europe	Germany	developed country	adult	3	5	1	0
Rhee et al., 2024a	3913	0.4340	2	2	North America	United States	developed country	adult	3	6	2	2
Rhee et al., 2024b	37042	0.0750	2	2	North America	United States	developed country	adult	3	6	2	2
Oh et al., 2024	913191	0.2580	2	3	Asia	South Korea	developed country	adolescent	2	6	2	2
Xin et al., 2020	24378	0.1290	2	2	Asia	China	developing country	adult	3	5	1	0
Yang et al., 2023	5380	0.1280	2	2	Asia	China	developing country	adolescent	2	5	2	2
Y Chen et al., 2023a	6587	0.1540	2	3	Asia	China	developing country	adolescent	3	5	1	2
Y Chen et al., 2023b	8139	0.4080	2	4	Asia	China	developing country	adolescent	2	5	2	2
Que et al., 2022	16220	0.1330	2	3	Asia	China	developing country	adult	4	5	1	2
Ramos-Martín et al., 2023	2212	0.1470	2	4	Europe	Spain	developed country	adult	3	6	1	1
Aymerich et al., 2024a	16969	0.0220	1	1	Europe	Spain	developed country	unknown	5	5	2	2
Aymerich et al., 2024b	16969	0.0423	2	5	Europe	Spain	developed country	unknown	5	5	2	2
Brady et al., 2022	390	0.1380	2	3	Europe	Ireland	developed country	adult	4	6	1	1
Sewall et al., 2021	384	0.2900	2	3	North America	United States	developed country	adult	3	5	1	0
SM Al-Humadi et al., 2021	225	0.0670	2	3	North America	United States	developed country	adult	5	5	1	0
Z Chen et al., 2023	1077	0.2898	2	4	North America	United States	developed country	adult	3	6	2	2
L Liu, Pollock, et al., 2023a	12344	0.0240	2	3	North America	Canada	developed country	adult	3	6	2	2
L Liu, Pollock, et al., 2023b	6592	0.0420	2	4	North America	Canada	developed country	adult	3	6	2	2
Asieieva et al., 2022a	223	0.4439	2	3	Europe	Ukraine	developing country	adult	4	5	2	2
Asieieva et al., 2022b	137	0.4891	2	3	Europe	Ukraine	developing country	adult	1	5	2	2
Asieieva et al., 2022c	223	0.4350	2	4	Europe	Ukraine	developing country	adult	4	5	2	2
Asieieva et al., 2022d	137	0.4745	2	4	Europe	Ukraine	developing country	adult	1	5	2	2
Ibeziako et al., 2022a	2020	0.4970	1	1	North America	United States	developed country	adolescent	3	5	2	2
Ibeziako et al., 2022b	1779	0.6030	2	3	North America	United States	developed country	adolescent	3	5	2	2
Lozano-Verduzco et al., 2024	1525	0.2350	2	3	South America	Mexico	developing country	mixed	2	5	2	2
Kim et al., 2024a	117343	0.1320	1	1	Asia	South Korea	developed country	adolescent	5	6	1	2
Kim et al., 2024b	109796	0.1180	2	3	Asia	South Korea	developed country	adolescent	5	6	2	2
Olié et al., 2022	296	0.2770	2	3	Europe	France	developed country	adult	3	5	1	0
Galletta et al., 2022	184	0.1430	2	3	South America	Brazil	developing country	adult	4	6	1	2
Bayazit et al., 2022	1833	0.0970	2	3	Europe	Turkey	developing country	adult	3	5	2	2
Essadek et al., 2023	823	0.2539	2	3	Europe	France	developed country	adult	2	5	1	0
Byeon, 2022	54948	0.1090	2	3	Asia	South Korea	developed country	adolescent	5	6	2	2
Lázaro-Pérez et al., 2023	1472	0.3240	2	5	Europe	Spain	developed country	adult	4	6	1	2
Supasitthumrong et al., 2024a	4676	0.0986	2	3	Asia	Thailand	developing country	adult	3	6	2	2
Supasitthumrong et al., 2024b	9595	0.0486	2	3	Asia	Thailand	developing country	adult	3	6	2	2
Peng et al., 2023	39751	0.2030	2	3	Asia	China	developed country	adolescent	3	5	1	0
S Liang et al., 2022a	164101	0.0850	2	2	Asia	China	developing country	adult	3	5	1	0
S Liang et al., 2022b	148384	0.1100	2	3	Asia	China	developing country	adult	3	5	2	2
S Liang et al., 2022c	159187	0.1430	2	3	Asia	China	developing country	adult	3	5	2	2
Wong et al., 2023	2540	0.2000	2	3	Asia	China	developing country	mixed	3	6	1	1
Shi et al., 2021a	27149	0.1900	2	3	Asia	China	developing country	adult	1	5	1	0
Shi et al., 2021b	29530	0.1400	2	3	Asia	China	developing country	adult	4	5	2	2
Turner et al., 2022	809	0.4400	2	3	North America	Canada	developed country	adolescent	3	5	2	2
Lo et al., 2024	14709	0.1530	2	5	Asia	China	developing country	adult	3	5	1	0
Kaggwa, Arinaitwe, Nduhuura, et al., 2022	540	0.1389	2	4	Africa	Uganda	developing country	adult	2	6	1	0
Tantirattanakulchai et al., 2023	314	0.3250	2	5	Asia	Thailand	developing country	old	3	5	1	1
Nahrin et al., 2023	1523	0.1440	2	5	Asia	Bangladesh	developing country	adult	1	5	2	2
Adeyinka et al., 2023	666	0.0620	2	5	North America	Canada	developed country	mixed	3	6	2	2
Sun et al., 2024	9858	0.0780	2	6	Asia	China	developing country	adult	4	5	2	2
Pouradeli et al., 2023	1421	0.0920	2	4	Asia	Iran	developing country	adult	2	6	1	2
Xu et al., 2021	11254	0.0194	2	3	Asia	China	developing country	mixed	3	5	1	0
Dumont et al., 2024	492	0.1440	2	4	Europe	Switzerland	developed country	adolescent	3	6	2	2
Hall et al., 2023	3230	0.0380	2	5	Asia	China	developing country	adult	2	6	1	2
Knudsen et al., 2021a	563	0.0320	2	2	Europe	Norway	developed country	adult	3	6	2	2
Knudsen et al., 2021b	691	0.0420	2	3	Europe	Norway	developed country	adult	3	6	2	2
Knudsen et al., 2021c	530	0.0320	2	3	Europe	Norway	developed country	adult	3	6	2	2

Knudsen et al., 2021d	370	0.0410	2	3	Europe	Norway	developed country	adult	3	6	2	2
Villarreal Sotelo et al., 2023	659	0.3990	2	3	South America	Mexico	developing country	adult	3	5	1	1
Statistics Canada, 2022a	6592	0.0270	1	1	North America	Canada	developed country	adult	3	6	1	2
Statistics Canada, 2022b	6592	0.0420	2	4	North America	Canada	developed country	adult	3	6	1	2
Husky, Léon, et al., 2024	13926	0.0680	2	3	Europe	France	developed country	adult	2	6	2	2
L Zhang et al., 2022	1718	0.6640	2	3	Asia	China	developing country	adult	3	6	2	2
Na et al., 2021	661	0.1920	2	3	North America	United States	developed country	adult	1	6	1	0
Villanueva-Silvestre et al., 2022	921	0.0660	2	3	Europe	Spain	developed country	adult	3	5	1	0
Guerrero and Barnes, n.d.	22721	0.0016	2	3	North America	Canada	developed country	adult	2	5	2	2
She et al., 2022	3136	0.2980	2	3	Asia	China	developing country	adolescent	3	6	1	0
Ferrando et al., 2021a	202	0.4670	1	1	North America	United States	developed country	adolescent	3	5	2	2
Ferrando et al., 2021b	153	0.4180	1	1	North America	United States	developed country	adult	2	5	2	2
Ferrando et al., 2021c	65	0.4310	2	3	North America	United States	developed country	adolescent	3	5	2	2
Ferrando et al., 2021d	136	0.4410	2	3	North America	United States	developed country	adult	2	5	2	2
Sun et al., 2021	1912	0.1956	2	2	Asia	China	developing country	adult	3	5	2	2
Malandain et al., 2022	263	0.1330	2	3	Europe	France	developed country	adult	1	4	2	2
Guo et al., 2023a	105	0.1430	1	1	Asia	China	developing country	adolescent	4	5	2	2
Guo et al., 2023b	93	0.0860	2	2	Asia	China	developing country	adolescent	3	5	2	2
Guo et al., 2023c	142	0.0630	2	4	Asia	China	developing country	adolescent	3	5	2	2
Schmits et al., 2021	23307	0.2080	2	4	Europe	Belgium	developed country	adult	3	5	1	2
Tsai et al., 2021	6607	0.2460	2	3	North America	United States	developed country	adult	3	5	1	2
Bi et al., 2023	381	0.2860	2	4	unknown	unknown	unknown	adult	3	5	1	0
Rifai et al., 2023	483	0.0290	2	4	Asia	China	developing country	adult	3	5	1	2
Every-Palmer et al., 2020	2010	0.0610	2	3	Europe	New Zealand	developed country	adult	3	6	2	2
Bell et al., 2022a	624	0.1200	2	3	Europe	New Zealand	developed country	adult	3	6	2	2
Bell et al., 2022b	2765	0.0240	2	3	Europe	New Zealand	developed country	adult	3	6	2	2
Benavente-Fernández et al., 2022	36	0.1670	2	3	Europe	Spain	developed country	adult	2	7	1	1
Rodríguez-De Avila et al., 2021	988	0.5700	2	3	unknown	unknown	developing country	adult	4	5	1	2
Rathod et al., 2020	7917	0.3200	2	3	unknown	unknown	unknown	mixed	4	5	2	2
Elhadi et al., 2020	2430	0.2270	2	3	Africa	Libya	developing country	adult	4	5	1	0
Lu et al., 2021a	326	0.1600	2	3	Asia	China	developing country	adult	5	5	2	2
Lu et al., 2021b	1304	0.1070	2	3	Asia	China	developing country	adult	5	5	2	2
Muneeb and Hassan, 2023	372	0.1638	2	3	Asia	Pakistan	developing country	adult	4	4	1	2
Rizzi et al., 2022	107	0.0100	2	3	Europe	Italy	developed country	adult	3	5	2	2
Généreux and Landaverde, 2022	20327	0.1720	2	3	North America	Canada	developed country	adult	2	6	2	2
Fong et al., 2023	1472	0.1010	2	4	Asia	China	developing country	adult	3	6	2	2
Fadipe et al., 2021	160	0.0380	2	3	Africa	Nigeria	developing country	mixed	2	6	2	2
Kim, 2024	189	0.2110	2	3	Africa	South Africa	developing country	adult	4	5	1	2
Stickley et al., 2023	1452	0.1170	2	3	Asia	Japan	developed country	adult	3	5	2	2
Ren et al., 2020	1172	0.0280	2	3	Asia	China	developing country	mixed	3	5	1	2
McKnight-Eily et al., 2021	1004	0.0840	2	3	North America	United States	developed country	adult	5	5	2	2
Scoresby et al., 2023	2208	0.1730	2	4	North America	United States	developed country	adult	4	5	2	2
X Zhang et al., 2022	12917	0.0910	2	5	Asia	China	developing country	adult	3	5	1	0
Selak et al., 2024	4645	0.2660	2	4	Europe	Slovenia	developed country	adult	3	5	1	0
N. Fountoulakis et al., 2023	12792	0.0517	2	3	unknown	unknown	unknown	adult	3	5	1	2
Tsuno and Tabuchi, 2021	16384	0.1200	2	3	Asia	Japan	developed country	adult	2	6	2	2
Mediavilla et al., 2021	2370	0.0700	2	3	Europe	Spain	developed country	adult	4	5	1	1
Yao et al., 2023	5211	0.0961	2	4	Asia	China	developing country	adult	3	6	1	2
Park and Park, 2024	640	0.0300	2	4	Asia	South Korea	developed country	mixed	2	5	2	2
Agyapong et al., 2022	146	0.1780	2	4	North America	Canada	developed country	adult	4	6	1	0
Leaune et al., 2022	1765	0.0442	2	3	Europe	France	developed country	adult	4	6	1	2
Al-Mamun et al., 2023	9730	0.0500	2	3	Asia	Bangladesh	developing country	adult	2	5	2	2
Eleftheriou et al., 2021	562	0.1670	2	4	Europe	Greece	developed country	adult	3	5	2	2
Gómez-García et al., 2023	79665	0.1710	2	3	South America	Mexico	developing country	adult	3	5	2	2
Yang, Song, et al., 2021	19515	0.0740	2	3	Asia	China	developing country	adult	4	5	1	0
Teksin, 2020	452	0.0180	2	3	Europe	Turkey	developing country	adult	3	5	2	2
Mosolova et al., 2021	2195	0.0240	2	3	Europe	Russia	developing country	adult	3	5	1	0
Jadir and Anderson-Carpenter, 2022	2482	0.3970	2	3	unknown	unknown	unknown	adult	2	5	1	0
AlAbdulla et al., 2022	799	0.1710	2	2	Asia	Qatar	developing country	adult	2	6	2	2
Kirič et al., 2022	1966	0.5450	2	7	Europe	Slovenia	developed country	adolescent	3	6	2	2
Casas Muñoz et al., 2024	6775	0.4412	2	4	South America	Mexico	developed country	adolescent	3	6	1	2
Kaggwa, Arinaitwe, Muwanguzi, et al., 2022	540	0.3185	2	4	Africa	Uganda	developing country	adult	2	6	1	2
Tasnim et al., 2020	3331	0.1280	2	3	Asia	Bangladesh	developing country	adult	2	5	2	2
Geda et al., 2022	4005	0.1200	2	4	North America	Canada	developed country	adult	3	6	2	2
Roy et al., 2023	410	0.2390	2	4	Asia	Bangladesh	developing country	adult	2	6	2	2
Gratz et al., 2021	452	0.2120	2	3	North America	United States	developed country	adult	3	4	2	2
L Liu, Contreras, et al., 2023a	2096	0.0420	2	3	North America	Canada	developed country	adult	3	6	2	2
L Liu, Contreras, et al., 2023b	1159	0.0800	2	4	North America	Canada	developed country	adult	3	6	2	2
Jones et al., 2023	4693	0.1890	2	7	North America	Canada	developed country	adult	3	6	1	2
X Chen et al., 2023	1297	0.0670	2	4	Asia	China	developing country	adult	2	5	2	2
Zikun Ma et al., 2022	5670	0.1330	2	3	Asia	China	developing country	adult	2	5	2	2
Ferro et al., 2024	6950	0.0700	1	1	North America	Canada	developed country	adolescent	5	7	2	2
Shiraly et al., 2022	803	0.0860	2	3	Asia	Iran	developing country	old	3	6	1	2
Y-J Liang et al., 2022	1159	0.0410	2	3	Asia	China	developing country	old	3	5	1	2
Shongwe and Huang, 2021	993	0.0150	2	3	Africa	Eswatini	developing country	adult	3	6	2	2
Xiao et al., 2024	82873	0.1250	2	5	Asia	China	developing country	adolescent	3	5	1	2
Papadopoulou et al., 2021	5116	0.0520	2	3	Europe	Greece	developed country	adult	3	5	1	0
Rudenstine et al., 2022	2364	0.2000	2	3	North America	United States	developed country	adult	3	5	1	0
Lin et al., 2022	10843	0.2080	2	4	Asia	Iran	developing country	adult	3	5	1	0
Seidler et al., 2023	700	0.1280	2	4	Oceania	Australia	developed country	adult	1	4	2	2
S Li et al., 2023	14690	0.0904	2	3	Asia	China	developing country	adult	3	5	2	2
Gómez et al., 2023	1005	0.3580	2	5	Europe	Spain	developed country	adult	4	5	1	2
Gelezelyte et al., 2022	474	0.3190	1	1	Europe	Lithuania	developing country	adult	4	6	1	2
Lyngdoh et al., 2023	345	0.1590	2	5	Asia	India	developing country	adult	3	4	1	2
Badrfam et al., 2023	305	0.2200	2	4	Asia	Iran	developing country	adult	2	5	1	2
Bukuluki et al., 2021	219	0.1330	2	3	Africa	Uganda	developing country	adolescent	3	5	2	2
Solis et al., 2023	301	0.0800	2	3	Europe	Spain	developed country	adult	4	5	1	2
De La Vega Sánchez et al., 2023	3140	0.1732	2	3	Europe	Spain	developed country	adult	2	5	1	2
Menculini et al., n.d.	447	0.0920	2	3	Europe	Italy	developed country	adult	3	6	2	2
Hou et al., 2021	761	0.3640	2	3	Asia	China	developing country	adolescent	2	6	1	2

L Liu, Batomen, et al., 2023a	147793	0.0240	1	1	North America	Canada	developed country	mixed	3	6	2	2
L Liu, Batomen, et al., 2023b	24926	0.0200	2	3	North America	Canada	developed country	mixed	3	6	2	2
Mohammed A Mamun et al., 2021	756	0.0820	2	4	Asia	Bangladesh	developing country	adult	2	6	1	2
Li et al., 2022a	513	0.5830	2	3	Asia	China	developing country	adult	4	5	2	2
Li et al., 2022b	392	0.3320	2	3	Asia	China	developing country	adult	3	5	2	2
DeVylder et al., 2021	16315	0.1340	2	3	North America	United States	developed country	adult	3	6	2	2
Cai et al., 2023	759	0.4850	2	3	Asia	China	developing country	adolescent	3	5	1	0
Yu et al., 2022	1248	0.1434	2	3	Asia	China	developing country	adolescent	3	5	1	2
Gómez Delgado et al., 2024	3583	0.1710	2	4	South America	Mexico	developing country	adolescent	3	5	1	2
Lantos et al., 2022a	22896	0.1110	1	1	North America	United States	developed country	adolescent	3	6	1	2
Lantos et al., 2022b	14635	0.1220	2	2	North America	United States	developed country	adolescent	3	6	2	2
Wijesinghe et al., 2023	188	0.0410	2	5	Asia	Sri Lanka	developing country	adult	3	5	2	2
Doukas et al., 2023	421	0.1320	2	3	North America	United States	developed country	adult	4	5	1	0
Merkle et al., 2023	7971	0.0430	2	5	North America	United States	developed country	adult	4	6	1	0
Koné et al., 2022	22862	0.0810	2	5	North America	United States	developed country	adult	4	5	1	0
Xiao et al., 2023	2186037	0.0014	2	7	North America	United States	developed country	adult	3	5	2	2
Keyworth et al., 2022	1029	0.1030	2	3	Europe	United Kingdom	developed country	adult	3	5	2	2
Z Li et al., 2023	1343	0.0968	2	5	Asia	China	developing country	adult	3	5	1	0
Chen et al., 2021	2700	0.0540	2	3	Asia	China	developing country	adult	4	5	1	2
Hamdan et al., 2025	911	0.1910	2	3	Asia	Israel	developing country	adult	4	5	1	2
Mohammed A. Mamun et al., 2021	10067	0.0500	2	3	Asia	Bangladesh	developing country	adult	2	5	2	2
Tintori et al., 2023	4288	0.4490	2	4	Europe	Italy	developed country	unknown	2	6	2	2
Yang, Yip, et al., 2021	1070	0.0160	2	3	Asia	China	developing country	adult	3	6	1	0
Medvedeva et al., 2020	908	0.1300	2	3	Europe	Russia	developing country	adult	5	5	2	2
Bilgi et al., 2021	178	0.2300	2	3	Europe	Turkey	developing country	adult	3	5	2	2
Beames et al., 2023	5866	0.0510	2	3	Oceania	Australia	developed country	adolescent	2	6	1	2
Keshavarzi et al., n.d. a	383	0.1400	2	4	Asia	Malaysia	developing country	adult	3	5	2	2
Keshavarzi et al., n.d. b	16839	0.0950	2	3	Asia	South Korea	developed country	adolescent	5	5	2	2
De Chiara et al., 2022	72	0.0420	2	3	Europe	Italy	developed country	adult	4	6	1	2
Gournellis and Efstathiou, 2021	5748	0.0520	2	3	Europe	Greece	developed country	adult	5	4	2	2
Kone et al., 2022	26174	0.0750	2	4	North America	United States	developed country	adult	5	5	1	0
Rozanova et al., 2022	123	0.1060	2	3	Europe	Ukraine	developing country	old	2	5	2	2
Lam et al., 2024	1008	0.0840	2	5	Asia	China	developing country	adult	3	5	2	2
De Miquel et al., 2022	2381	0.0380	2	3	Europe	Spain	developed country	adult	2	6	1	1
Cai et al., 2020a	1173	0.0900	2	3	Asia	China	developing country	adult	3	5	2	2
Cai et al., 2020b	1173	0.1200	2	3	Asia	China	developing country	adult	3	5	2	2
M Sun et al., 2023	881	0.0330	1	1	Asia	China	developing country	adolescent	3	5	1	2
Lo et al., 2023	407	0.3140	2	5	Asia	China	developing country	adult	2	6	1	0
Yao et al., 2024a	4633	0.0389	2	4	Asia	China	developing country	adult	3	6	2	2
Yao et al., 2024b	5011	0.0581	2	5	Asia	China	developing country	adult	3	6	2	2
Yao et al., 2024c	4410	0.0433	2	6	Asia	China	developing country	adult	3	6	2	2
Murata et al., 2021a	4326	0.1600	2	3	North America	United States	developed country	adult	5	5	2	2
Murata et al., 2021b	583	0.3800	2	3	North America	United States	developed country	adolescent	5	5	2	2
Rathod et al., 2020a	29133	0.3100	2	3	Europe	United Kingdom	developed country	unknown	5	5	1	0
Rathod et al., 2020b	83851	0.3000	2	4	Europe	United Kingdom	developed country	unknown	5	5	2	2
Rathod et al., 2020c	75204	0.1900	2	4	Europe	United Kingdom	developed country	unknown	5	5	2	2
Regaieg et al., 2022	97	0.1040	2	4	Africa	Tunisia	developing country	adult	3	4	2	2
Alghamdi et al., 2022	491	0.0652	2	3	Asia	Saudi Arabia	developing country	adult	4	6	1	2
Gesi et al., 2024	931	0.1080	2	3	Europe	Italy	developed country	adult	3	5	1	0
Urbańska and Słysz, 2023	499	0.0600	2	4	Europe	Poland	developing country	adult	3	5	1	2
Knowles et al., 2022	12989	0.0930	2	3	Europe	United Kingdom	developed country	mixed	4	5	2	2
Jaramillo et al., 2025	818	0.1050	2	3	South America	United States	developed country	adult	3	5	1	2
Y Chen et al., 2024a	689	0.0770	2	5	Asia	China	developing country	adult	4	7	2	2
Y Chen et al., 2024b	456	0.0830	2	6	Asia	China	developing country	adult	4	6	2	2
Y Chen et al., 2024c	300	0.0630	2	6	Asia	China	developing country	adult	4	6	2	2
Y Chen et al., 2024d	601	0.1480	2	6	Asia	China	developing country	adult	3	6	2	2
Zhai and Du, 2022	354473	0.1320	2	3	North America	United States	developed country	adult	3	5	2	2
Killgore et al., 2020a	3120	0.1760	2	3	North America	United States	developed country	mixed	3	4	1	0
Killgore et al., 2020b	3120	0.3070	2	3	North America	United States	developed country	mixed	5	4	2	2
Kohls et al., 2023a	3382	0.1960	2	3	Europe	Germany	developed country	adult	5	5	1	0
Kohls et al., 2023b	5642	0.1450	2	4	Europe	Germany	developed country	adult	5	5	2	2
Kohls et al., 2023c	5510	0.1650	2	5	Europe	Germany	developed country	adult	3	5	2	2
Kaparounaki et al., 2020	1000	0.0970	2	3	Europe	Greece	developed country	adult	3	5	1	2
Okubo et al., 2021	24819	0.1200	2	3	Asia	Japan	developed country	unknown	3	6	2	2
Kang et al., 2023	1014	0.4210	2	4	Asia	South Korea	developed country	adult	2	5	2	2
Brady, Shackleton, et al., 2023a	390	0.1400	2	3	Europe	Ireland	developed country	adult	5	5	1	1
Brady, Shackleton, et al., 2023b	229	0.2360	2	4	Europe	Ireland	developed country	adult	4	5	2	2
Stickley et al., 2025	3371	0.093	2	6	Asia	Japan	developed country	mixed	3	5	2	2
Yang et al., 2025	131	0.252	2	3	North America	United States	developed country	adult	3	5	1	2
Zisook et al., 2022a	1171	0.105	1	1	North America	United States	developed country	mixed	5	4	1	2
Zisook et al., 2022b	1134	0.094	2	2	North America	United States	developed country	mixed	5	7	2	2
Goodman et al., 2023	655	0.146	2	5	North America	United States	developed country	adult	4	2	1	2
Ritish et al., 2020	1602	0.019	2	3	Asia	India	developing country	mixed	2	5	2	2
Frey et al., 2024	536	0.487	2	4	Asia	India	developing country	adult	3	5	1	0
Haas et al., 2024	448	0.223	2	3	South America	Brazil	developing country	adult	3	5	2	2
Prawira et al., 2023	484	0.355	2	4	Asia	Indonesia	developing country	adult	3	5	1	0
Zwickl et al., 2023	1019	0.49	2	3	Oceania	Australia	developed country	mixed	3	5	1	0
Bismark et al., 2022	7795	0.105	2	3	Oceania	Australia	developed country	mixed	4	5	1	0
Kim et al., 2025a	45056	0.021	2	4	North America	United States	developed country	adolescent	2	6	1	2
Kim et al., 2025b	135869	0.007	2	4	North America	United States	developed country	adolescent	2	6	1	2
Macalli et al., 2025a	4463	0.211	1	2	Europe	France	developed country	adult	3	5	2	2
Macalli et al., 2025b	1768	0.293	2	5	Europe	France	developed country	adult	3	5	2	2
Hassan et al., 2025	9743	0.0365	2	5	North America	Canada	developed country	mixed	3	6	2	2
Song and Kim, 2025	12828	0.054	2	4	Asia	South Korea	developed country	adult	2	6	2	2
Ide et al., 2025a	2635	0.1	2	6	Asia	Japan	developed country	adult	4	6	1	2
Ide et al., 2025b	2813	0.086	2	4	Asia	Japan	developed country	adult	4	6	1	2
Özçelik et al., 2025	490	0.069	2	4	Asia	Turkey	developing country	adult	3	5	1	2
Goodwill and Taylor, n.d.	995	0.065	2	5	North America	United States	developed country	adult	3	6	2	2
Seedat et al., 2025	1211	0.218	2	3	Africa	South Africa	developing country	adult	3	5	1	2
Giannakopoulos et al., 2025	612	0.281	2	6	Europe	Greece	developed country	adolescent	3	5	2	2
Sørensen et al., 2025a	2286	0.42	2	3	Europe	Denmark	developed country	mixed	3	6	2	2

Sørensen et al., 2025b	1041	0.3	2	4	Europe	Denmark	developed country	mixed	3	6	2	2
Sørensen et al., 2025c	1039	0.28	2	5	Europe	Denmark	developed country	mixed	3	6	2	2
Yang, 2025	7628	0.199	2	4	North America	United States	developed country	adolescent	3	6	1	2
Fisch et al., 2025	4136	0.221	2	4	North America	United States	developed country	mixed	3	5	1	2
Hamdan et al., 2025	911	0.191	2	3	Asia	Israel	developed country	adult	4	5	1	2
Jaramillo et al., 2025	818	0.105	2	3	North America	United States	developed country	adult	3	5	1	2
Van Der Feltz-Cornelis et al., 2025	328	0.11	2	5	Europe	United Kingdom	developed country	adult	4	6	1	2

Longitudinal studies

Time	Citation	sample1	sample2	T1	T2	Area	Country	Age	Gender	Economic
Pre- vs. post-pandemic										
	Gouin et al., 2023	1235	1325	10.20%	8.70%	North America	Canada	adult	3	developed
	Zheng et al., 2024	489	333	0.01%	5.71%	Asian	China	adult	3	developing
	Zhu et al., 2021	1491	1393	24.30%	21.10%	Asian	China	adolescent	3	developing
	Bountress et al., 2022	897	897	38.20%	12.70%	North America	USA	adolescent	4	developed
	Zhu & Wong, 2022	1491	1393	24.46%	21.04%	Asian	China	adolescent	2	developing
	Bacchi et al., 2024	4191	2177	1.80%	5.60%	South America	Brazil	adult	3	developing
	Ayuso-Mateos et al., 2023	1881	1103	1.56%	2.78%	Europe	Spanish	adult	3	developing
	Wang et al., 2021	67905	67905	7.60%	10%	Asian	China	adult	3	developing
	Fisch et al., 2025	4136	2539	22.00%	23%	North America	USA	Mixed	3	developed
Early-to-mid-and-late pandemic										
	Efstathiou et al., 2022	5116	720	5.20%	4.72%	Europe	Greece	adult	3	developed
	Danzo et al., 2024	623	623	9.30%	11.10%	North America	USA	adolescent	3	developed
	Domínguez-González et al., 2022	247	247	17.81%	18.63%	North America	Mexico	adult	3	developing
	Efstathiou et al., 2021	5116	811	4.81%	4.32%	Europe	Greece	adult	3	developing
	Wu et al., 2023	1236	1209	23.00%	24.10%	North America	USA	adolescent	2	developed
	Sasaki et al., 2021	875	875	24.90%	28.70%	Asian	Japan	adult	2	developed
	Lewis et al., 2023	184	69	30.60%	31.90%	North America	USA	adult	4	developed
	Antonelli-Salgado et al., 2021	1674	1674	22.60%	20.30%	South America	Brazil	adult	4	developing
	Ori et al., 2023	36106	36106	0.59%	0.95%	Europe	Holland	adult	3	developed
	Nomura et al., 2022	985	985	5.80%	11.80%	Asian	Japan	adult	3	developed
	Palagini et al., 2023	81	81	6.10%	1.20%	Europe	Italy	old	2	developed
	López Steinmetz et al., 2021	1202	1202	37.77%	42.35%	South America	Argentina	adult	4	developing
	Padmanathan et al., 2023	12514	7160	10.00%	9.00%	Europe	UK	none	3	developed
	Husky et al., 2024	829	387	28.60%	32.40%	Europe	France	none	3	developed
	Huang et al., 2022	164101	35516	7.30%	12.60%	Asian	China	adult	3	developing
	Batterham et al., 2022	1296	519	17.10%	16.20%	Oceania	Australia	adult	3	developed
	Ma et al., 2022	68685	68685	7.60%	10.00%	Asian	China	adult	3	developing
	Hano et al., 2025	232	232	1.70%	4.30%	North America	America	adolescent	3	developed

Supplementary Materials 6. Risk of bias assessment

Cross-sectional studies

Citation	Score_1	Score_2
Delgadillo et al., 2023	6	6
Zhang et al., 2024	7	7
Sanchez Merino et al., 2024	4	4
Yamashita et al., 2024	7	7
Goodwill and Ajibewa, 2024	6	6
Monteith et al., 2023	6	6
Gonzalez Mendez et al., 2022	5	5
Peng et al., 2022	5	5
C Park et al., 2023	5	5
Rus Prelog et al., 2023	5	5
M Wang et al., 2021	6	6
Moderato et al., 2021	5	5
Yau and Nager, 2021	5	5
Rabasco et al., 2021	5	5
Höller and Forkmann, 2022	5	5
Gao et al., 2023	5	5
Wu et al., 2022	5	5
Essadek et al., 2022	7	7
Bressington et al., 2020	5	5
Yamazaki et al., 2022	6	5
Valladares-Garrido et al., 2024	5	5
Wei et al., 2023	5	5
El Frenn et al., 2023	5	6
Kabir et al., 2023	5	5
Liu et al., 2021	5	6
Rogers et al., 2023	5	5
Daly et al., 2021	6	6
Narita et al., 2023	5	5
Stickley et al., 2022	6	6
Woolverton et al., 2024	6	5
Carlos et al., 2023	5	5
Kleinhendler-Lustig et al., 2023	5	5
Ernst et al., 2022	6	6
Raviv et al., 2021	5	5
Workneh et al., 2023	6	6
Rogers et al., 2024	5	6
Kim et al., 2022	6	6
Nigatu et al., 2023	6	5

Spiller et al., 2023	6	5
B Park et al., 2023	6	6
Sivertsen et al., 2022	6	6
Vidal et al., 2024	5	5
Rappaport et al., 2024	6	6
Lee et al., 2024	6	6
Kim et al., 2021	5	6
Escobar-Agreda et al., 2023	6	6
Mailloux et al., 2024	5	6
Molina et al., 2023	6	6
Macalli et al., 2022	5	5
Kundu et al., 2022	5	5
Sharif et al., 2020	5	5
Bantjes et al., 2023	5	5
Blay Benzaken et al., 2023	5	5
J Liu et al., 2023	6	6
Osiogo et al., 2021	6	6
Iftene et al., 2022	4	4
Han et al., 2023	6	6
Li et al., 2020	5	5
Burke et al., 2022	6	6
Luethy et al., 2023	5	5
Choi et al., 2021	5	5
Wen et al., 2024	6	6
Sahimi et al., 2021	5	6
MacDonald et al., 2022	6	6
M-Y Chen et al., 2024	5	5
Zhou et al., 2021	5	5
Ali et al., 2022	6	6
**Matić et al., 2022**	6	6
Drucker et al., 2023	4	4
Villanueva-Blasco et al., 2024	5	5
S Al-Humadi et al., 2021	5	5
Macalli et al., 2025	5	5
Brady, Fenton, et al., 2023	5	5
Joshi et al., 2022	4	5
Bonello et al., 2021	5	6
Deng et al., 2023	5	5
Richardson et al., 2022	5	5
Mallhi et al., 2022	5	5

Wathelet, Vincent, et al., 2022	5	5
Gadermann et al., 2021	6	6
Mzumara, 2024	5	5
Hamm et al., 2020	6	5
Pouradeli et al., 2024	6	7
Benavides Morales and López Peláez, 2022	4	5
Wathelet et al., 2020	5	5
Lizhi et al., 2021	5	5
Pelissier et al., 2021	5	5
Saeed et al., 2024	6	6
Crisol-Deza et al., 2023	5	5
Trettel et al., 2022	7	7
Park and Lee, 2022	7	7
Taniguchi et al., 2022	7	7
Meller et al., 2022	7	7
Mohd Fadhli et al., 2022	7	7
Shaygan et al., 2024	7	7
Zhu et al., 2022	6	6
Cheung et al., 2021	5	5
Ali et al., 2023	5	5
Young et al., 2021	5	5
Prado et al., 2023	5	5
Mucci et al., 2022	6	6
Hong et al., 2021	5	5
Molina et al., 2024	5	5
Brasso et al., 2023	6	6
Elhadi et al., 2022	4	4
Erjavac et al., 2023	4	4
Jeong et al., 2023	6	6
McLoughlin et al., 2022	6	6
Sato et al., 2004	5	5
Thomson et al., 2021	6	6
Reyniers et al., 2022	6	6
Reinke et al., 2023	6	5
Goodyear et al., 2021	6	6
Peper-Nascimento et al., 2024	5	5
Gainza Perez et al., 2022	5	5
Mushtaque et al., 2024	5	5
Li et al., 2024	6	6
Shewangzaw Engda et al., 2022	7	7

Wang et al., 2020	5	5
Lee and Nam, 2023	6	6
John et al., 2021	6	5
Ahuvia et al., n.d.	5	5
Howard et al., 2022	5	5
Matsushima et al., 2023	6	6
Jupina et al., 2022	5	5
Žilinskas et al., 2021	6	6
Leroy et al., 2021	5	5
Ramirez et al., 2022	7	7
Fountoulakis et al., 2022	5	5
Kholmogorova et al., 2021	5	5
Sljivo et al., 2022	6	6
Bustos Villarroel et al., 2024	5	5
Rolland et al., 2022	6	5
Dale et al., 2023	5	5
Arsandaux et al., 2021	5	5
Rutkowska et al., 2022	5	5
Killgore et al., 2021	6	6
Chodkiewicz et al., 2021	5	5
Troya et al., 2022	7	7
Chakrabarti, 2021	5	5
Schad et al., 2022	5	5
Frajerman et al., 2022	5	5
Frajerman, 2023	7	7
Dooley et al., 2024	5	5
Oliveira et al., 2022	5	5
Beymer et al., 2023	5	5
Niederkrotenthaler et al., 2022	6	6
An et al., 2022	5	5
Genis-Mendoza et al., 2021	5	5
Tsiouris et al., 2023	5	5
Jilowa et al., 2022	5	5
Korkmaz et al., 2022	5	5
Stonehouse et al., 2023	5	5
Wathelet, Horn, et al., 2022	6	5
Kohls et al., 2021	5	5
Czeisler et al., 2021	7	7
Landi et al., 2023	5	5
He et al., 2023	5	5

Dogan-Sander et al., 2021	5	5
Rhee et al., 2024	6	6
Oh et al., 2024	6	6
Xin et al., 2020	5	5
Yang et al., 2023	5	6
Y Chen et al., 2023	5	6
Que et al., 2022	5	5
Ramos-Martín et al., 2023	6	6
Aymerich et al., 2024	5	5
Brady et al., 2022	6	6
Sewall et al., 2021	5	5
SM Al-Humadi et al., 2021	5	5
Z Chen et al., 2023	6	6
L Liu, Pollock, et al., 2023	6	6
Asieieva et al., 2022	5	5
Ibeziako et al., 2022	5	6
Lozano-Verduzco et al., 2024	5	5
Kim et al., 2024	6	6
Olié et al., 2022	5	5
Galletta et al., 2022	6	6
Bayazit et al., 2022	5	5
Essadek et al., 2023	5	5
Byeon, 2022	6	6
Lázaro-Pérez et al., 2023	6	5
Supasitthumrong et al., 2024	6	5
Peng et al., 2023	5	5
S Liang et al., 2022	5	6
Wong et al., 2023	6	6
Shi et al., 2021	5	5
Turner et al., 2022	5	5
Lo et al., 2024	5	5
Kaggwa, Arinaitwe, Nduhuura, et al., 2022	6	6
Tantirattanakulchai et al., 2023	5	6
Nahrin et al., 2023	5	5
Adeyinka et al., 2023	6	5
Sun et al., 2024	5	5
Pouradeli et al., 2023	6	6
Xu et al., 2021	5	5
Dumont et al., 2024	6	6
Hall et al., 2023	6	6

Knudsen et al., 2021	6	6
Villarreal Sotelo et al., 2023	5	5
Statistics Canada, 2022	6	6
Husky, Léon, et al., 2024	6	6
L Zhang et al., 2022	6	6
Na et al., 2021	6	6
Villanueva-Silvestre et al., 2022	5	5
Guerrero and Barnes, n.d.	5	5
She et al., 2022	6	6
Ferrando et al., 2021	5	5
Sun et al., 2021	5	5
Malandain et al., 2022	4	4
Guo et al., 2023	5	5
Schmits et al., 2021	5	5
Tsai et al., 2021	5	5
Bi et al., 2023	5	5
Rifai et al., 2023	5	5
Every-Palmer et al., 2020	6	6
Bell et al., 2022	6	6
Benavente-Fernández et al., 2022	7	7
Rodríguez-De Avila et al., 2021	5	5
Rathod et al., 2020	5	5
Elhadi et al., 2020	5	5
Lu et al., 2021	5	5
Muneeb and Hassan, 2023	4	5
Rizzi et al., 2022	5	5
Généreux and Landaverde, 2022	6	6
Fong et al., 2023	6	6
Fadipe et al., 2021	6	6
Kim, 2024	5	6
Stickley et al., 2023	5	6
Ren et al., 2020	5	5
McKnight-Eily et al., 2021	5	5
Scoresby et al., 2023	5	5
X Zhang et al., 2022	5	5
Selak et al., 2024	5	5
N. Fountoulakis et al., 2023	5	5
Tsuno and Tabuchi, 2021	6	6
Mediavilla et al., 2021	5	5
Yao et al., 2023	6	5

Park and Park, 2024	5	5
Agyapong et al., 2022	6	6
Leaune et al., 2022	6	6
Al-Mamun et al., 2023	5	5
Eleftheriou et al., 2021	5	5
Gómez-García et al., 2023	5	5
Yang, Song, et al., 2021	5	5
Teksin, 2020	5	5
Mosolova et al., 2021	5	5
Jadir and Anderson-Carpenter, 2022	5	5
AlAbdulla et al., 2022	6	6
Kirič et al., 2022	6	6
Casas Muñoz et al., 2024	6	6
Kaggwa, Arinaitwe, Muwanguzi, et al., 2022	6	6
Tasnim et al., 2020	5	5
Geda et al., 2022	6	6
Roy et al., 2023	6	6
Gratz et al., 2021	4	5
L Liu, Contreras, et al., 2023	6	6
Jones et al., 2023	6	6
X Chen et al., 2023	5	6
Zikun Ma et al., 2022	5	6
Ferro et al., 2024	7	6
Shiraly et al., 2022	6	6
Y-J Liang et al., 2022	5	5
Shongwe and Huang, 2021	6	7
Xiao et al., 2024	5	5
Papadopoulou et al., 2021	5	5
Rudenstine et al., 2022	5	5
Lin et al., 2022	5	5
Seidler et al., 2023	4	5
S Li et al., 2023	5	5
Gómez et al., 2023	5	5
Gelezelyte et al., 2022	6	6
Lyngdoh et al., 2023	4	5
Badrfam et al., 2023	5	5
Bukuluki et al., 2021	5	6
Solis et al., 2023	5	5
De La Vega Sánchez et al., 2023	5	5
Menculini et al., n.d.	6	6

Hou et al., 2021	6	6
L Liu, Batomen, et al., 2023	6	6
Mohammed A Mamun et al., 2021	6	6
Li et al., 2022	5	6
DeVylder et al., 2021	6	6
Cai et al., 2023	5	5
Yu et al., 2022	5	5
Gómez Delgado et al., 2024	5	5
Lantos et al., 2022	6	6
Wijesinghe et al., 2023	5	5
Doukas et al., 2023	5	5
Merkle et al., 2023	6	5
Koné et al., 2022	5	5
Xiao et al., 2023	5	6
Keyworth et al., 2022	5	5
Z Li et al., 2023	5	6
Chen et al., 2021	5	6
Hamdan et al., 2025	5	5
Mohammed A. Mamun et al., 2021	5	5
Tintori et al., 2023	6	6
Yang, Yip, et al., 2021	6	6
Medvedeva et al., 2020	5	5
Bilgi et al., 2021	5	5
Beames et al., 2023	6	6
Keshavarzi et al., n.d.	5	5
De Chiara et al., 2022	6	6
Gournellis and Efstathiou, 2021	4	5
Kone et al., 2022	5	5
Rozanova et al., 2022	5	5
Lam et al., 2024	5	6
De Miquel et al., 2022	6	6
Cai et al., 2020	5	5
M Sun et al., 2023	5	5
Lo et al., 2023	6	6
Yao et al., 2024	6	5
Murata et al., 2021	5	5
Rathod et al., 2020	5	5
Regaieg et al., 2022	4	4
Alghamdi et al., 2022	6	6
Gesi et al., 2024	5	5

Urbańska and Słysz, 2023	5	5
Knowles et al., 2022	5	5
Jaramillo et al., 2025	5	5
Y Chen et al., 2024	7	7
Zhai and Du, 2022	5	6
Killgore et al., 2020	4	5
Kohls et al., 2023	5	5
Kaparounaki et al., 2020	5	5
Okubo et al., 2021	6	6
Kang et al., 2023	5	6
Brady, Shackleton, et al., 2023	5	5
Stickley et al., 2025	5	5
Yang et al., 2025	5	5
Zisook et al., 2022	5	6
Goodman et al., 2023	2	3
Ritish et al., 2020	5	5
Frey et al., 2024	5	5
Haas et al., 2024	5	5
Prawira et al., 2023	5	5
Zwickl et al., 2023	5	5
Bismark et al., 2022	5	5
Kim et al., 2025	6	6
Macalli et al., 2025	5	5
Hassan et al., 2025	6	6
Song and Kim, 2025	6	6
Ide et al., 2025	6	6
Özçelik et al., 2025	5	5
Goodwill and Taylor, n.d.	6	6
Seedat et al., 2025	5	5
Giannakopoulos et al., 2025	5	5
Sørensen et al., 2025	6	6
Yang, 2025	6	6
Fisch et al., 2025	5	5
Hamdan et al., 2025	5	5
Jaramillo et al., 2025	5	5
Van Der Feltz-Cornelis et al., 2025	6	6

Longitudinal studies

Citation	Score_1	Score_2
Zheng et al., 2024	6	6
Wang et al., 2021	8	8
Zhu et al., 2021	8	8
Gouin et al., 2023	7	7
Bountress et al., 2022	7	7
Ayuso-Mateos et al., 2023	7	8
Zhu & Ching, 2021	8	8
Bacchi et al., 2024	7	7
Ori et al., 2023	7	7
Husky et al., 2024	7	7
Lewis et al., 2023	7	7
Palagini et al., 2023	7	7
Nomura et al., 2022	7	6
Sasaki et al., 2021	6	6
Danzo et al., 2024	7	7
Padmanathan et al., 2023	7	7
Efstathiou et al., 2022	7	7
Efstathiou et al., 2021	7	8
Antonelli-Salgado et al., 2021	7	7
Domínguez-González et al., 2022	8	8
Wu et al., 2023	7	7
Batterham et al., 2022	7	7
López Steinmetz et al., 2021	7	7
Ma et al., 2022	7	7
Huang et al., 2022	7	7
Fisch et al., 2025	7	7
Hano et al., 2025	7	7

Supplementary Materials 7. Funnel plot after trim and fill

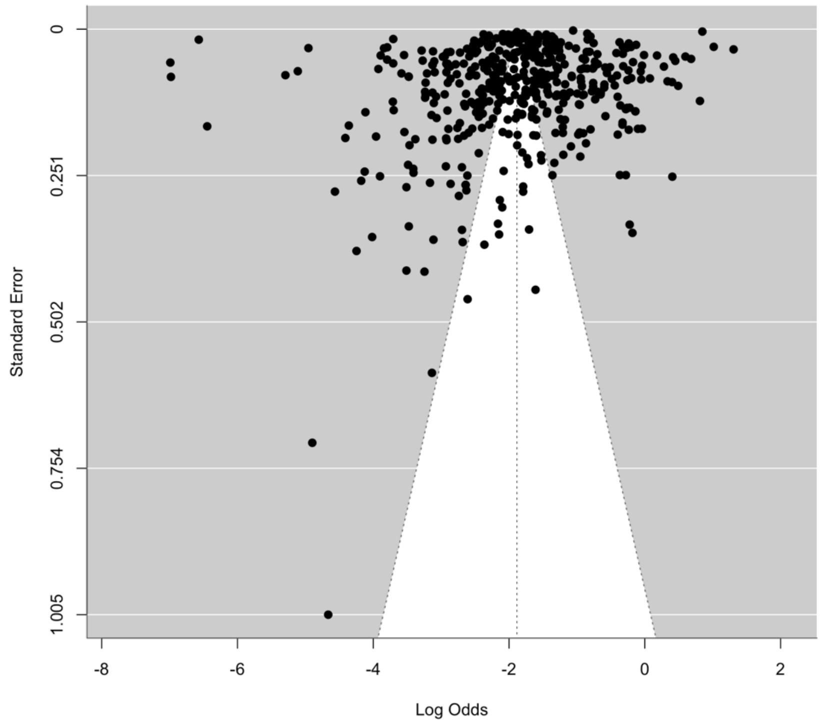

Supplementary Materials 8. Forest plot

Forest Plot of All Cross−Sectional Studies(k=474)

Study, Country	Sample Size (n)	Prevalence (SI)	Proportion [95% CI]
1, Austria	1005	17.3%	0.17 [0.15, 0.20]
2, United Kingdom	1006	31.7%	0.32 [0.29, 0.35]
3, China	208	6.7%	0.07 [0.04, 0.11]
4, Spain	450	39.1%	0.39 [0.35, 0.44]
5, Japan	200	23.0%	0.23 [0.18, 0.29]
6, United States	487	9.7%	0.10 [0.07, 0.13]
7, United States	567	5.1%	0.05 [0.04, 0.07]
8, China	1263	8.3%	0.08 [0.07, 0.10]
9, China	6183	22.0%	0.22 [0.21, 0.23]
10, United States	394	15.9%	0.16 [0.13, 0.20]
11, United States	222	16.0%	0.16 [0.12, 0.22]
12, Italy	1790	9.7%	0.10 [0.08, 0.11]
13, China	460	23.3%	0.23 [0.20, 0.27]
14, Italy	858	3.0%	0.03 [0.02, 0.04]
15, United States	1261	1.0%	0.01 [0.01, 0.02]
16, United States	216	43.0%	0.43 [0.37, 0.50]
17, Germany	1311	21.7%	0.22 [0.20, 0.24]
18, China	949	16.1%	0.16 [0.14, 0.19]
19, China	686	5.2%	0.05 [0.04, 0.07]
20, France	8004	14.2%	0.14 [0.13, 0.15]
21, France	3611	14.9%	0.15 [0.14, 0.16]
22, France	7535	20.9%	0.21 [0.20, 0.22]
23, China	11072	22.5%	0.22 [0.22, 0.23]
24, Japan	98	20.4%	0.20 [0.14, 0.30]
25, Peru	370	34.3%	0.34 [0.30, 0.39]
26, China	1338	9.7%	0.10 [0.08, 0.11]
27, Lebanon	402	18.2%	0.18 [0.15, 0.22]
28, Bangladesh	1264	13.3%	0.13 [0.12, 0.15]
29, China	5175	3.0%	0.03 [0.03, 0.03]
30, none	5528	46.8%	0.47 [0.45, 0.48]
31, Canada	3000	5.9%	0.06 [0.05, 0.07]
32, United States	1077	29.0%	0.29 [0.26, 0.32]
33, Japan	1452	11.7%	0.12 [0.10, 0.13]
34, United States	1956	28.6%	0.29 [0.27, 0.31]
35, United States	134	17.6%	0.18 [0.12, 0.25]
36, Israel	160	15.0%	0.15 [0.10, 0.21]
37, Israel	86	10.5%	0.10 [0.06, 0.19]
38, Germany	144	15.3%	0.15 [0.10, 0.22]
39, Germany	2359	9.0%	0.09 [0.08, 0.10]
40, United States	32217	0.5%	0.00 [0.00, 0.01]
41, United States	32217	0.6%	0.01 [0.01, 0.01]
42, Ethiopia	300	3.0%	0.03 [0.02, 0.06]
43, Ethiopia	277	5.5%	0.05 [0.03, 0.09]
44, Brazil	127	40.2%	0.40 [0.32, 0.49]
45, Canada	65	60.0%	0.60 [0.48, 0.71]
46, Germany	532	43.6%	0.44 [0.39, 0.48]
47, India	302	27.5%	0.27 [0.23, 0.33]
48, Israel	195	35.9%	0.36 [0.29, 0.43]
49, Poland	309	69.3%	0.69 [0.64, 0.74]
50, Russia	561	51.9%	0.52 [0.48, 0.56]
51, South Korea	1043	18.0%	0.18 [0.16, 0.20]
52, Turkey	434	35.4%	0.35 [0.31, 0.40]
53, United States	1970	64.5%	0.65 [0.62, 0.67]
54, South Korea	54948	10.9%	0.11 [0.11, 0.11]
55, South Korea	54848	12.7%	0.13 [0.12, 0.13]
56, Canada	3003	7.2%	0.07 [0.06, 0.08]
57, United States	1232	12.8%	0.13 [0.11, 0.15]
58, South Korea	61018	9.1%	0.09 [0.09, 0.09]
59, South Korea	56325	8.8%	0.09 [0.09, 0.09]
60, South Korea	56874	17.3%	0.17 [0.17, 0.18]
61, South Korea	52922	15.0%	0.15 [0.15, 0.15]
62, Norway	21405	15.7%	0.16 [0.15, 0.16]
63, Norway	40807	14.7%	0.15 [0.14, 0.15]
64, United States	270153	70.0%	0.70 [0.70, 0.70]
65, United States	8652	21.9%	0.22 [0.21, 0.23]
66, South Korea	99055	9.2%	0.09 [0.09, 0.09]
67, South Korea	48443	12.6%	0.13 [0.12, 0.13]
68, South Korea	44216	10.1%	0.10 [0.10, 0.10]
69, Peru	249	6.8%	0.07 [0.04, 0.11]
70, Canada	2398	22.2%	0.22 [0.21, 0.24]
71, Spain	297	16.8%	0.17 [0.13, 0.22]
72, France	1913	12.7%	0.13 [0.11, 0.14]
73, Canada	1414	61.0%	0.61 [0.58, 0.64]
74, none	375	5.0%	0.05 [0.03, 0.08]
75, United States	47684	2.2%	0.02 [0.02, 0.02]
76, South Africa	504	60.0%	0.60 [0.56, 0.64]
77, South Africa	448	62.0%	0.62 [0.57, 0.66]
78, Israel	158	30.9%	0.31 [0.24, 0.39]
79, Israel	66	40.9%	0.41 [0.30, 0.53]
80, United States	4692	21.0%	0.21 [0.20, 0.22]
81, Canada	6041	14.4%	0.14 [0.14, 0.15]
82, Canada	508	19.5%	0.19 [0.16, 0.23]
83, South Korea	309	40.1%	0.40 [0.35, 0.46]
84, South Korea	1055	19.1%	0.19 [0.17, 0.22]
85, China	1970	10.8%	0.11 [0.10, 0.12]
86, United States	121	23.1%	0.23 [0.16, 0.31]
87, United States	112	14.0%	0.14 [0.09, 0.22]
88, South Korea	31720	8.3%	0.08 [0.08, 0.09]
89, China	4768	5.4%	0.05 [0.05, 0.06]
90, Malaysia	171	11.1%	0.11 [0.07, 0.17]
91, United States	3113	1.6%	0.02 [0.01, 0.02]
92, United States	496	1.4%	0.01 [0.01, 0.03]
93, China	10647	6.2%	0.06 [0.06, 0.07]
94, China	11133	7.3%	0.07 [0.07, 0.08]
95, Bangladesh	731	16.3%	0.16 [0.14, 0.19]
96, Slovenia	587	14.0%	0.14 [0.11, 0.17]
97, Slovenia	511	28.4%	0.28 [0.25, 0.32]
98, Israel	104	28.0%	0.28 [0.20, 0.37]
99, Spain	3780	5.1%	0.05 [0.04, 0.06]
100, United States	225	6.6%	0.07 [0.04, 0.11]
101, France	4463	21.1%	0.21 [0.20, 0.22]
102, France	1768	29.3%	0.29 [0.27, 0.31]
103, Ireland	377	13.0%	0.13 [0.10, 0.17]
104, United Kingdom	125	6.0%	0.06 [0.03, 0.12]
105, United States	1415	16.3%	0.16 [0.14, 0.18]
106, Malta	401	11.8%	0.12 [0.09, 0.15]
107, Malta	350	23.2%	0.23 [0.19, 0.28]
108, China	7684	16.3%	0.16 [0.15, 0.17]
109, China	14921	30.1%	0.30 [0.29, 0.31]
110, Canada	469	21.5%	0.22 [0.18, 0.25]
111, Canada	214	6.1%	0.06 [0.04, 0.10]
112, Canada	1519	4.3%	0.04 [0.03, 0.05]
113, Canada	807	2.0%	0.02 [0.01, 0.03]
114, Saudi Arabia	1074	32.0%	0.32 [0.29, 0.35]
115, France	68891	11.4%	0.11 [0.11, 0.12]
116, France	22540	13.2%	0.13 [0.13, 0.14]
117, Canada	618	8.3%	0.08 [0.06, 0.11]
118, Canada	2382	5.2%	0.05 [0.04, 0.06]
119, Malawi	620	16.3%	0.16 [0.14, 0.19]
120, United States	73	7.0%	0.07 [0.03, 0.15]
121, Iran	202	46.5%	0.47 [0.40, 0.53]
122, Colombia	33	45.4%	0.45 [0.30, 0.62]
123, France	69054	11.4%	0.11 [0.11, 0.12]
124, China	18521	2.8%	0.03 [0.03, 0.03]
125, France	740	14.7%	0.15 [0.12, 0.17]
126, Iran	353	34.3%	0.34 [0.30, 0.39]
127, Peru	1238	17.9%	0.18 [0.16, 0.20]
128, Brazil	4203	19.2%	0.19 [0.18, 0.20]
129, South Korea	784	9.2%	0.09 [0.07, 0.11]
130, Japan	990	15.0%	0.15 [0.13, 0.17]
131, Japan	1043	5.2%	0.05 [0.04, 0.07]
132, Japan	21389	3.6%	0.04 [0.03, 0.04]
133, Brazil	2152	3.8%	0.04 [0.03, 0.05]
134, Malaysia	1290	11.9%	0.12 [0.10, 0.14]
135, Iran	703	28.0%	0.28 [0.25, 0.31]
136, China	5175	3.0%	0.03 [0.03, 0.03]
137, United States	717	21.5%	0.21 [0.19, 0.25]
138, Canada	508	14.2%	0.14 [0.11, 0.17]
139, United Kingdom	845	7.6%	0.08 [0.06, 0.10]
140, Brazil	8376	7.6%	0.08 [0.07, 0.08]
141, Philippines	454	24.9%	0.25 [0.21, 0.29]
142, South Korea	658	10.3%	0.10 [0.08, 0.13]
143, China	12509	21.3%	0.21 [0.21, 0.22]
144, Turkey	782	25.5%	0.25 [0.23, 0.29]
145, Saudi Arabia	214	16.7%	0.17 [0.12, 0.22]
146, United States	1685	5.0%	0.05 [0.04, 0.06]
147, Brazil	2437	33.2%	0.33 [0.31, 0.35]
148, Italy	115	20.7%	0.21 [0.14, 0.29]
149, Italy	65	15.4%	0.15 [0.08, 0.26]
150, Italy	241	41.0%	0.41 [0.35, 0.47]
151, China	4692	6.5%	0.07 [0.06, 0.07]
152, Spain	870	7.9%	0.08 [0.06, 0.10]
153, Italy	154	27.3%	0.27 [0.21, 0.35]
154, Italy	160	41.9%	0.42 [0.34, 0.50]
155, Libya	31557	7.6%	0.08 [0.07, 0.08]
156, United States	573	45.0%	0.45 [0.41, 0.49]
157, South Korea	2417	1.2%	0.01 [0.01, 0.02]
158, South Korea	2932	1.3%	0.01 [0.01, 0.02]
159, Ireland	36	43.8%	0.44 [0.29, 0.61]
160, Canada	6592	4.2%	0.04 [0.04, 0.05]
161, Japan	558	10.2%	0.10 [0.08, 0.13]
162, Canada	618	8.4%	0.08 [0.06, 0.11]
163, Canada	804	8.3%	0.08 [0.07, 0.10]
164, Canada	602	7.9%	0.08 [0.06, 0.10]
165, Belgium	965	20.3%	0.20 [0.18, 0.23]
166, Belgium	965	21.0%	0.21 [0.19, 0.24]
167, United States	330080	0.1%	0.00 [0.00, 0.00]
168, United States	160320	0.1%	0.00 [0.00, 0.00]
169, Canada	502	16.9%	0.17 [0.14, 0.20]
170, Brazil	2259	10.9%	0.11 [0.10, 0.12]
171, United States	159	27.7%	0.28 [0.21, 0.35]
172, Pakistan	241	22.4%	0.22 [0.18, 0.28]
173, China	5380	12.8%	0.13 [0.12, 0.14]
174, Ethiopia	700	11.0%	0.11 [0.09, 0.14]
175, United States	2031	18.0%	0.18 [0.16, 0.20]
176, South Korea	2000	20.3%	0.20 [0.19, 0.22]
177, United Kingdom	2221	7.7%	0.08 [0.07, 0.09]
178, United Kingdom	4246	9.4%	0.09 [0.09, 0.10]
179, United Kingdom	4382	10.0%	0.10 [0.09, 0.11]
180, United States	415	27.0%	0.27 [0.23, 0.31]
181, United States	323	19.2%	0.19 [0.15, 0.24]
182, United States	270	0.7%	0.01 [0.00, 0.03]
183, Japan	153	28.8%	0.29 [0.22, 0.36]
184, Japan	615	16.8%	0.17 [0.14, 0.20]
185, United States	960	7.2%	0.07 [0.06, 0.09]
186, Lithuania	1001	37.5%	0.37 [0.35, 0.41]
187, France	4193	9.1%	0.09 [0.08, 0.10]
188, France	5431	11.5%	0.12 [0.11, 0.12]
189, France	59404	11.6%	0.12 [0.11, 0.12]
190, Chile	5037	20.5%	0.21 [0.19, 0.22]
191, Greece	507	7.3%	0.07 [0.05, 0.10]
192, Russia	110	11.0%	0.11 [0.06, 0.18]
193, Bosnia and Herzegovina	827	8.6%	0.09 [0.07, 0.11]
194, Chile	125	29.9%	0.30 [0.22, 0.38]
195, France	11754	19.0%	0.19 [0.18, 0.20]
196, Austria	1257	33.3%	0.33 [0.31, 0.36]
197, Austria	1257	45.7%	0.46 [0.43, 0.48]
198, France	1919	10.1%	0.10 [0.09, 0.12]
199, Poland	753	18.0%	0.18 [0.15, 0.21]
200, United States	1013	17.6%	0.18 [0.15, 0.20]
201, Poland	618	23.6%	0.24 [0.20, 0.27]
202, Ireland	1983	3.8%	0.04 [0.03, 0.05]
203, India	590	5.0%	0.05 [0.04, 0.07]
204, United States	309	19.0%	0.19 [0.15, 0.24]
205, United States	622	6.0%	0.06 [0.04, 0.08]
206, France	1925	19.0%	0.19 [0.17, 0.21]
207, france	11754	19.0%	0.19 [0.18, 0.20]
208, Ireland	4404	42.0%	0.42 [0.41, 0.43]
209, Brazil	890	7.4%	0.07 [0.06, 0.09]
210, United States	21293	16.2%	0.16 [0.16, 0.17]
211, United States	10861	17.1%	0.17 [0.16, 0.18]
212, Austria	12029	7.0%	0.07 [0.07, 0.07]
213, China	4940	78.7%	0.79 [0.78, 0.80]
214, China	5550	73.4%	0.73 [0.72, 0.75]
215, Mexico	1011	4.5%	0.04 [0.03, 0.06]
216, Germany	4351	16.0%	0.16 [0.15, 0.17]
217, Germany	3066	16.3%	0.16 [0.15, 0.18]
218, Germany	1438	21.4%	0.21 [0.19, 0.24]
219, India	207	2.9%	0.03 [0.01, 0.06]
220, Turkey	573	19.2%	0.19 [0.16, 0.23]
221, South Africa	295	12.0%	0.12 [0.09, 0.16]
222, France	68106	11.4%	0.11 [0.11, 0.12]
223, France	22205	13.2%	0.13 [0.13, 0.14]
224, France	44898	13.8%	0.14 [0.13, 0.14]
225, Germany	3382	14.5%	0.14 [0.13, 0.16]
226, Australia	1157	16.9%	0.17 [0.15, 0.19]
227, Italy	652	15.3%	0.15 [0.13, 0.18]
228, China	3006	10.9%	0.11 [0.10, 0.12]
229, China	3465	7.6%	0.08 [0.07, 0.09]
230, China	3917	8.4%	0.08 [0.08, 0.09]
231, Germany	5642	16.5%	0.17 [0.16, 0.17]
232, United States	3913	43.4%	0.43 [0.42, 0.45]
233, United States	37042	7.5%	0.07 [0.07, 0.08]
234, South Korea	913191	25.8%	0.26 [0.26, 0.26]
235, China	24378	12.9%	0.13 [0.12, 0.13]
236, China	5380	12.8%	0.13 [0.12, 0.14]
237, China	6587	15.4%	0.15 [0.15, 0.16]
238, China	8139	40.8%	0.41 [0.40, 0.42]
239, China	16220	13.3%	0.13 [0.13, 0.14]
240, Spain	2212	14.7%	0.15 [0.13, 0.16]
241, Spain	16969	2.2%	0.02 [0.02, 0.02]
242, Spain	16969	4.2%	0.04 [0.04, 0.05]
243, Ireland	390	13.8%	0.14 [0.11, 0.18]
244, United States	384	29.0%	0.29 [0.25, 0.34]
245, United States	225	6.7%	0.07 [0.04, 0.11]
246, United States	1077	29.0%	0.29 [0.26, 0.32]
247, Canada	12344	2.4%	0.02 [0.02, 0.03]
248, Canada	6592	4.2%	0.04 [0.04, 0.05]
249, Ukraine	223	44.4%	0.44 [0.38, 0.51]
250, Ukraine	137	48.9%	0.49 [0.41, 0.57]
251, Ukraine	223	43.5%	0.43 [0.37, 0.50]
252, Ukraine	137	47.4%	0.47 [0.39, 0.56]
253, United States	2020	49.7%	0.50 [0.48, 0.52]
254, United States	1779	60.3%	0.60 [0.58, 0.63]
255, Mexico	1525	23.5%	0.23 [0.21, 0.26]
256, South Korea	117343	13.2%	0.13 [0.13, 0.13]
257, South Korea	109796	11.8%	0.12 [0.12, 0.12]
258, France	296	27.7%	0.28 [0.23, 0.33]
259, Brazil	184	14.3%	0.14 [0.10, 0.20]
260, Turkey	1833	9.7%	0.10 [0.08, 0.11]
261, France	823	25.4%	0.25 [0.23, 0.28]
262, South Korea	54948	10.9%	0.11 [0.11, 0.11]
263, Spain	1472	32.4%	0.32 [0.30, 0.35]
264, Thailand	4676	9.9%	0.10 [0.09, 0.11]
265, Thailand	9595	4.9%	0.05 [0.04, 0.05]
266, China	39751	20.3%	0.20 [0.20, 0.21]
267, China	164101	8.5%	0.09 [0.08, 0.09]
268, China	148384	11.0%	0.11 [0.11, 0.11]
269, China	159187	14.3%	0.14 [0.14, 0.14]
270, China	2540	20.0%	0.20 [0.18, 0.22]
271, China	27149	19.0%	0.19 [0.19, 0.19]
272, China	29530	14.0%	0.14 [0.14, 0.14]
273, Canada	809	44.0%	0.44 [0.41, 0.47]
274, China	14709	15.3%	0.15 [0.15, 0.16]
275, Uganda	540	13.9%	0.14 [0.11, 0.17]
276, Thailand	314	32.5%	0.32 [0.28, 0.38]
277, Bangladesh	1523	14.4%	0.14 [0.13, 0.16]
278, Canada	666	6.2%	0.06 [0.05, 0.08]
279, China	9858	7.8%	0.08 [0.07, 0.08]
280, Iran	1421	9.2%	0.09 [0.08, 0.11]
281, China	11254	1.9%	0.02 [0.02, 0.02]
282, Switzerland	492	14.4%	0.14 [0.12, 0.18]
283, China	3230	3.8%	0.04 [0.03, 0.05]
284, Norway	563	3.2%	0.03 [0.02, 0.05]
285, Norway	691	4.2%	0.04 [0.03, 0.06]
286, Norway	530	3.2%	0.03 [0.02, 0.05]
287, Norway	370	4.1%	0.04 [0.02, 0.07]
288, Mexico	659	39.9%	0.40 [0.36, 0.44]
289, Canada	6592	2.7%	0.03 [0.02, 0.03]
290, Canada	6592	4.2%	0.04 [0.04, 0.05]
291, France	13926	6.8%	0.07 [0.06, 0.07]
292, China	1718	66.4%	0.66 [0.64, 0.69]
293, United States	661	19.2%	0.19 [0.16, 0.22]
294, Spain	921	6.6%	0.07 [0.05, 0.08]
295, Canada	22721	0.2%	0.00 [0.00, 0.00]
296, China	3136	29.8%	0.30 [0.28, 0.31]
297, United States	202	46.7%	0.47 [0.40, 0.53]
298, United States	153	41.8%	0.42 [0.34, 0.50]
299, United States	65	43.1%	0.43 [0.32, 0.55]
300, United States	136	44.1%	0.44 [0.36, 0.53]
301, China	1912	19.6%	0.20 [0.18, 0.21]
302, France	263	13.3%	0.13 [0.10, 0.18]
303, China	105	14.3%	0.14 [0.09, 0.22]
304, China	93	8.6%	0.09 [0.04, 0.16]
305, China	142	6.3%	0.06 [0.03, 0.12]
306, Belgium	23307	20.8%	0.21 [0.20, 0.21]
307, United States	6607	24.6%	0.25 [0.24, 0.26]
308, none	381	28.6%	0.29 [0.24, 0.33]
309, China	483	2.9%	0.03 [0.02, 0.05]
310, New Zealand	2010	6.1%	0.06 [0.05, 0.07]
311, New Zealand	624	12.0%	0.12 [0.10, 0.15]
312, New Zealand	2765	2.4%	0.02 [0.02, 0.03]
313, Spain	36	16.7%	0.17 [0.08, 0.32]
314, none	988	57.0%	0.57 [0.54, 0.60]
315, none	7917	32.0%	0.32 [0.31, 0.33]
316, Libya	2430	22.7%	0.23 [0.21, 0.24]
317, China	326	16.0%	0.16 [0.12, 0.20]
318, China	1304	10.7%	0.11 [0.09, 0.13]
319, Pakistan	372	16.4%	0.16 [0.13, 0.21]
320, Italy	107	1.0%	0.01 [0.00, 0.06]
321, Canada	20327	17.2%	0.17 [0.17, 0.18]
322, China	1472	10.1%	0.10 [0.09, 0.12]
323, Nigeria	160	3.8%	0.04 [0.02, 0.08]
324, South Africa	189	21.1%	0.21 [0.16, 0.28]
325, Japan	1452	11.7%	0.12 [0.10, 0.13]
326, China	1172	2.8%	0.03 [0.02, 0.04]
327, United States	1004	8.4%	0.08 [0.07, 0.10]
328, United States	2208	17.3%	0.17 [0.16, 0.19]
329, China	12917	9.1%	0.09 [0.09, 0.10]
330, Slovenia	4645	26.6%	0.27 [0.25, 0.28]
331, none	12792	5.2%	0.05 [0.05, 0.06]
332, Japan	16384	12.0%	0.12 [0.12, 0.13]
333, Spain	2370	7.0%	0.07 [0.06, 0.08]
334, China	5211	9.6%	0.10 [0.09, 0.10]
335, South Korea	640	3.0%	0.03 [0.02, 0.05]
336, Canada	146	17.8%	0.18 [0.12, 0.25]
337, France	1765	4.4%	0.04 [0.04, 0.05]
338, Bangladesh	9730	5.0%	0.05 [0.05, 0.05]
339, Greece	562	16.7%	0.17 [0.14, 0.20]
340, Mexico	79665	17.1%	0.17 [0.17, 0.17]
341, China	19515	7.4%	0.07 [0.07, 0.08]
342, Turkey	452	1.8%	0.02 [0.01, 0.03]
343, Russia	2195	2.4%	0.02 [0.02, 0.03]
344, none	2482	39.7%	0.40 [0.38, 0.42]
345, Qatar	799	17.1%	0.17 [0.15, 0.20]
346, Slovenia	1966	54.5%	0.54 [0.52, 0.57]
347, Mexico	6775	44.1%	0.44 [0.43, 0.45]
348, Uganda	540	31.9%	0.32 [0.28, 0.36]
349, Bangladesh	3331	12.8%	0.13 [0.12, 0.14]
350, Canada	4005	12.0%	0.12 [0.11, 0.13]
351, Bangladesh	410	23.9%	0.24 [0.20, 0.28]
352, United States	452	21.2%	0.21 [0.18, 0.25]
353, Canada	2096	4.2%	0.04 [0.03, 0.05]
354, Canada	1159	8.0%	0.08 [0.07, 0.10]
355, Canada	4693	18.9%	0.19 [0.18, 0.20]
356, China	1297	6.7%	0.07 [0.05, 0.08]
357, China	5670	13.3%	0.13 [0.12, 0.14]
358, Canada	6950	7.0%	0.07 [0.06, 0.08]
359, Iran	803	8.6%	0.09 [0.07, 0.11]
360, China	1159	4.1%	0.04 [0.03, 0.05]
361, Eswatini	993	1.5%	0.02 [0.01, 0.02]
362, China	82873	12.5%	0.12 [0.12, 0.13]
363, Greece	5116	5.2%	0.05 [0.05, 0.06]
364, United States	2364	20.0%	0.20 [0.18, 0.22]
365, Iran	10843	20.8%	0.21 [0.20, 0.22]
366, Australia	700	12.8%	0.13 [0.11, 0.16]
367, China	14690	9.0%	0.09 [0.09, 0.10]
368, Spain	1005	35.8%	0.36 [0.33, 0.39]
369, Lithuania	474	31.9%	0.32 [0.28, 0.36]
370, India	345	15.9%	0.16 [0.12, 0.20]
371, Iran	305	22.0%	0.22 [0.18, 0.27]
372, Uganda	219	13.3%	0.13 [0.09, 0.18]
373, Spain	301	8.0%	0.08 [0.05, 0.12]
374, Spain	3140	17.3%	0.17 [0.16, 0.19]
375, Italy	447	9.2%	0.09 [0.07, 0.12]
376, China	761	36.4%	0.36 [0.33, 0.40]
377, Canada	147793	2.4%	0.02 [0.02, 0.02]
378, Canada	24926	2.0%	0.02 [0.02, 0.02]
379, Bangladesh	756	8.2%	0.08 [0.06, 0.10]
380, China	513	58.3%	0.58 [0.54, 0.62]
381, China	392	33.2%	0.33 [0.29, 0.38]
382, United States	16315	13.4%	0.13 [0.13, 0.14]
383, China	759	48.5%	0.48 [0.45, 0.52]
384, China	1248	14.3%	0.14 [0.13, 0.16]
385, Mexico	3583	17.1%	0.17 [0.16, 0.18]
386, United States	22896	11.1%	0.11 [0.11, 0.12]
387, United States	14635	12.2%	0.12 [0.12, 0.13]
388, Sri Lanka	188	4.1%	0.04 [0.02, 0.08]
389, United States	421	13.2%	0.13 [0.10, 0.17]
390, United States	7971	4.3%	0.04 [0.04, 0.05]
391, United States	22862	8.1%	0.08 [0.08, 0.08]
392, United States	2186037	0.1%	0.00 [0.00, 0.00]
393, United Kingdom	1029	10.3%	0.10 [0.09, 0.12]
394, China	1343	9.7%	0.10 [0.08, 0.11]
395, China	2700	5.4%	0.05 [0.05, 0.06]
396, Israel	911	19.1%	0.19 [0.17, 0.22]
397, Bangladesh	10067	5.0%	0.05 [0.05, 0.05]
398, Italy	4288	44.9%	0.45 [0.43, 0.46]
399, China	1070	1.6%	0.02 [0.01, 0.03]
400, Russia	908	13.0%	0.13 [0.11, 0.15]
401, Turkey	178	23.0%	0.23 [0.17, 0.30]
402, Australia	5866	5.1%	0.05 [0.05, 0.06]
403, Malaysia	383	14.0%	0.14 [0.11, 0.18]
404, South Korea	16839	9.5%	0.10 [0.09, 0.10]
405, Italy	72	4.2%	0.04 [0.01, 0.12]
406, Greece	5748	5.2%	0.05 [0.05, 0.06]
407, United States	26174	7.5%	0.07 [0.07, 0.08]
408, Ukraine	123	10.6%	0.11 [0.06, 0.17]
409, China	1008	8.4%	0.08 [0.07, 0.10]
410, Spain	2381	3.8%	0.04 [0.03, 0.05]
411, China	1173	9.0%	0.09 [0.08, 0.11]
412, China	1173	12.0%	0.12 [0.10, 0.14]
413, China	881	3.3%	0.03 [0.02, 0.05]
414, China	407	31.4%	0.31 [0.27, 0.36]
415, China	4633	3.9%	0.04 [0.03, 0.04]
416, China	5011	5.8%	0.06 [0.05, 0.06]
417, China	4410	4.3%	0.04 [0.04, 0.05]
418, United States	4326	16.0%	0.16 [0.15, 0.17]
419, United States	583	38.0%	0.38 [0.34, 0.42]
420, United Kingdom	29133	31.0%	0.31 [0.30, 0.32]
421, United Kingdom	83851	30.0%	0.30 [0.30, 0.30]
422, United Kingdom	75204	19.0%	0.19 [0.19, 0.19]
423, Tunisia	97	10.4%	0.10 [0.06, 0.18]
424, Saudi Arabia	491	6.5%	0.07 [0.05, 0.09]
425, Italy	931	10.8%	0.11 [0.09, 0.13]
426, Poland	499	6.0%	0.06 [0.04, 0.08]
427, United Kingdom	12989	9.3%	0.09 [0.09, 0.10]
428, United States	818	10.5%	0.11 [0.09, 0.13]
429, China	689	7.7%	0.08 [0.06, 0.10]
430, China	456	8.3%	0.08 [0.06, 0.11]
431, China	300	6.3%	0.06 [0.04, 0.10]
432, China	601	14.8%	0.15 [0.12, 0.18]
433, United States	354473	13.2%	0.13 [0.13, 0.13]
434, United States	3120	17.6%	0.18 [0.16, 0.19]
435, United States	3120	30.7%	0.31 [0.29, 0.32]
436, Germany	3382	19.6%	0.20 [0.18, 0.21]
437, Germany	5642	14.5%	0.14 [0.14, 0.15]
438, Germany	5510	16.5%	0.16 [0.16, 0.18]
439, Greece	1000	9.7%	0.10 [0.08, 0.12]
440, Japan	24819	12.0%	0.12 [0.12, 0.12]
441, South Korea	1014	42.1%	0.42 [0.39, 0.45]
442, Ireland	390	14.0%	0.14 [0.11, 0.18]
443, Ireland	229	23.6%	0.24 [0.19, 0.30]
444, Japan	3371	9.3%	0.09 [0.08, 0.10]
445, United States	131	25.2%	0.25 [0.18, 0.33]
446, United States	1171	10.5%	0.11 [0.09, 0.12]
447, United States	1134	9.4%	0.09 [0.08, 0.11]
448, United States	655	14.6%	0.15 [0.12, 0.18]
449, India	1602	1.9%	0.02 [0.01, 0.03]
450, India	536	48.7%	0.49 [0.44, 0.53]
451, Brazil	448	22.3%	0.22 [0.19, 0.26]
452, Indonesia	484	35.5%	0.36 [0.31, 0.40]
453, Australia	1019	49.0%	0.49 [0.46, 0.52]
454, Australia	7795	10.5%	0.10 [0.10, 0.11]
455, United States	45056	2.1%	0.02 [0.02, 0.02]
456, United States	135869	0.7%	0.01 [0.01, 0.01]
457, France	4463	21.1%	0.21 [0.20, 0.22]
458, France	1768	29.3%	0.29 [0.27, 0.31]
459, Canada	9743	3.6%	0.04 [0.03, 0.04]
460, South Korea	12828	5.4%	0.05 [0.05, 0.06]
461, Japan	2635	10.0%	0.10 [0.09, 0.11]
462, Japan	2813	8.6%	0.09 [0.08, 0.10]
463, Turkey	490	6.9%	0.07 [0.05, 0.10]
464, United States	995	6.5%	0.07 [0.05, 0.08]
465, South Africa	1211	21.8%	0.22 [0.20, 0.24]
466, Greece	612	28.1%	0.28 [0.25, 0.32]
467, Denmark	2286	42.0%	0.42 [0.40, 0.44]
468, Denmark	1041	30.0%	0.30 [0.27, 0.33]
469, Denmark	1039	28.0%	0.28 [0.25, 0.31]
470, United States	7628	19.9%	0.20 [0.19, 0.21]
471, United States	4136	22.1%	0.22 [0.21, 0.23]
472, Israel	911	19.1%	0.19 [0.17, 0.22]
473, United States	818	10.5%	0.11 [0.09, 0.13]
474, United Kingdom	328	11.0%	0.11 [0.08, 0.15]
Overall REML Model			0.13 [0.12, 0.14]

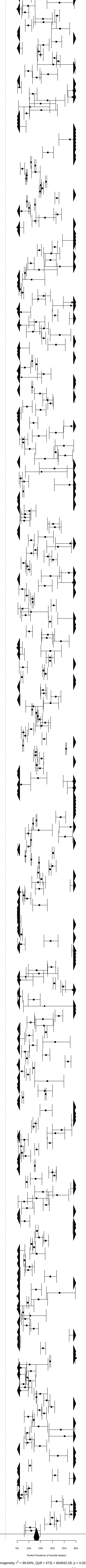

Heterogeneity: $I^2 = 99.94\%$, $Q(df = 473) = 664832.09$, $p < 0.001$

Supplementary Materials 9. All references of the studies included in the meta-analysis

Adeyinka DA, **Novik N**, **Novotna G**, **Bartram M**, **Gabrys R** and **Muhajarine N** (2023) Prevalence and factors associated with suicidal ideation, cannabis, and alcohol use during the COVID-19 pandemic in Saskatchewan: findings from a joint-effect modeling. *BMC Psychiatry* **23**(1), 571.

Agyapong B, **Shalaby R**, **Eboreime E**, **Wei Y** and **Agyapong VIO** (2022) Self-Reported Alcohol Abuse and the Desire to Receive Mental Health Counselling Predict Suicidal Thoughts/Thoughts of Self-Harm among Female Residents of Fort McMurray. *International Journal of Environmental Research and Public Health* **19**(20), 13620.

Ahuvia IL, **Dobias ML**, **Cohen KA**, **Nelson BD**, **Richmond LL**, **London B** and **Schleider JL** (n.d.) Loss of mental health support among college students during the COVID-19 pandemic. *Journal of American college health* **73**(3), 1198–1204.

AlAbdulla M, **Reagu SM**, **Hassan MHMO**, **Elhassan NM**, **Sayed S**, **Makki I**, **Elzain M**, **Wadoo O** and **Kumar R** (2022) Suicidal behavior in a migrant majority population and impact on trends during the early Covid-19 period: a cross sectional study in Qatar. *Discover Psychology* **2**(1), 28.

Alghamdi F, **Ashour A**, **Adeyemi L**, **Bamidele P**, **Nwambo-Logan B**, **Alsharif M**, **Sindi AM** and **Binmadi N** (2022) The Psychological Impacts of COVID-19 Pandemic among Emerging Adults: An Observational Cross-Sectional Study. *International Journal of Environmental Research and Public Health* **19**(3), 1445.

Al-Humadi S, **Bronson B**, **Muhlrad S**, **Paulus M**, **Hong H** and **Cáceda R** (2021) Depression, Suicidal Thoughts, and Burnout Among Physicians During the COVID-19 Pandemic: a Survey-Based Cross-Sectional Study. *Academic Psychiatry* **45**(5), 557–565.

Al-Humadi SM, **Cáceda R**, **Bronson B**, **Paulus M**, **Hong H** and **Muhlrad S** (2021) Orthopaedic Surgeon Mental Health During the COVID-19 Pandemic. *Geriatric Orthopaedic Surgery & Rehabilitation* **12**, 21514593211035230.

Ali AHA, **Alrudian N**, **Alqahtani MS**, **Alanazi SE**, **Alotaibi AZ**, **Almazroua AM**, **Abdalkareem MIA**, **Althubiti TA** and **Alanazi BG** (2023) Graduate Medical Students' Mental Health Concerns During COVID-19 Pandemic. *International Journal of Biomedicine* **13**(4).

Ali M, **Uddin Z**, **Amran Hossain KM** and **Uddin TR** (2022) Depression, anxiety, stress, and suicidal behavior among Bangladeshi undergraduate rehabilitation students: An observational study amidst the COVID-19 pandemic. *Health Science Reports* **5**(2), e549.

Al-Mamun F, **Hussain N**, **Sakib N**, **Hosen I**, **Rayhan I**, **Abdullah AH**, **Bhuiyan AKMI**, **Sarker MdA**, **Hossain S**, **Zou L**, **Manzar MdD**, **Lin C-Y**, **Sikder MdT**, **Muhit M**, **Pakpour AH**, **Gozal D**, **Griffiths MD** and **Mamun MA** (2023) Sleep duration during the COVID-19 pandemic in Bangladesh: A GIS-based large sample survey study. *Scientific Reports* **13**(1),

3368.

An J, **Yin Y**, **Zhao L**, **Tong Y** and **Liu NH** (2022) Mental health problems among hotline callers during the early stage of COVID-19 pandemic. *PeerJ* **10**, e13419.

Antonelli-Salgado T, **Monteiro GMC**, **Marcon G**, **Roza TH**, **Zimerman A**, **Hoffmann MS**, **Cao B**, **Hauck S**, **Brunoni AR** and **Passos IC** (2021) Loneliness, but not social distancing, is associated with the incidence of suicidal ideation during the COVID-19 outbreak: a longitudinal study. *Journal of Affective Disorders* **290**, 52–60.

Arsandaux J, **Montagni I**, **Macalli M**, **Texier N**, **Pouriel M**, **Germain R**, **Mebarki A**, **Kinouani S**, **Tournier M**, **Schuck S** and **Tzourio C** (2021) Mental health condition of college students compared to non-students during COVID-19 lockdown: the CONFINS study. *BMJ Open* **11**(8), e053231.

Asieieva Y, **Sytnik S**, **Babchuk O**, **Heina O** and **Dementieva K** (2022) Peculiarities of the mental state of student youth of Ukraine during quarantine restrictions. *Revista Amazonia Investiga* **11**(50), 9–15.

Aymerich C, **Pedruzo B**, **Salazar De Pablo G**, **Olazabal N**, **Catalan A** and **González-Torres MÁ** (2024) Number and nature of psychiatric emergency department visits in a tertiary hospital before, during, and after coronavirus pandemic. *Frontiers in Psychiatry* **15**, 1380401.

Ayuso-Mateos JL, **Morillo D**, **Haro JM**, **Olaya B**, **Lara E** and **Miret M** (2023) Changes on depression and suicidal ideation under severe lockdown restrictions during the first wave of the COVID-19 pandemic in Spain: a longitudinal study in the general population. *Epidemiology and Psychiatric Sciences* **32**, e55.

Bacchi P, **Suen P**, **Fatori D**, **Razza LB**, **Afonso L**, **Klein I**, **Cavendish B**, **Moreno ML**, **Santos IS**, **Benseñor I**, **Lotufo P** and **Brunoni AR** (2024) Incidence of suicidal ideation in a cohort of civil servants during the COVID-19 pandemic in Brazil: insights from the ELSA-Brasil Study. *Trends in Psychiatry and Psychotherapy*. Epub ahead of print 2024. DOI: 10.47626/2237-6089-2023-0701.

Badrfam R, **Zandifar A**, **Mohammadian Khonsari N** and **Qorbani M** (2023) Suicidal ideation, burnout, and their correlation among health care workers at the end of the fourth wave of the COVID-19 pandemic in Alborz Province, Iran. *Frontiers in Psychiatry* **14**, 1261105.

Bantjes J, **Swanevelder S**, **Jordaan E**, **Sampson NA**, **Petukhova MV**, **Lochner C**, **Stein DJ** and **Kessler RC** (2023) COVID-19 and common mental disorders among university students in South Africa. *South African Journal of Science* **119**(1/2).

Batterham PJ, **Calear AL**, **Shou Y**, **Farrer LM**, **Gulliver A**, **McCallum SM** and **Dawel A** (2022) Effects of the COVID-19 pandemic on suicidal ideation in a representative Australian population sample–Longitudinal cohort study. *Journal of Affective Disorders* **300**, 385–391.

Bayazit H, **Ozel M**, **Arac S**, **Dulgeroglu-Bayazit D** and **Joshi A** (2022) Posttraumatic Stress Disorder Among Health Care Workers During the COVID-19 Pandemic. *Journal of Psychiatric Practice* **28**(5), 354–361.

Beames JR, **Huckvale K**, **Fujimoto H**, **Maston K**, **Batterham PJ**, **Calear AL**, **Mackinnon A**, **Werner-Seidler A** and **Christensen H** (2023) The impact of COVID-19 and bushfires on the mental health of Australian adolescents: a cross-sectional study. *Child and Adolescent Psychiatry and Mental Health* **17**(1), 34.

Becker RA and Wilks AR (2023) *maps: Draw geographical maps* (Version 3.4.1) [R pa ckage]. Comprehensive R Archive Network. Available at: https://CRAN.R-project.org/p ackage=maps

Bell C, **Williman J**, **Beaglehole B**, **Stanley J**, **Jenkins M**, **Gendall P**, **Rapsey C** and **Every-Palmer S** (2022) Psychological distress, loneliness, alcohol use and suicidality in New Zealanders with mental illness during a strict COVID-19 lockdown. *Australian & New Zealand Journal of Psychiatry* **56**(7), 800–810.

Benavente-Fernández A, **Gutiérrez-Rojas L**, **Torres-Parejo Ú**, **Parejo Morón AI**, **Fernández Ontiveros S**, **Vinuesa García D**, **González-Domenech P** and **Laínez Ramos-Bossini AJ** (2022) Psychological Impact and Risk of Suicide in Hospitalized COVID-19 Patients, During the Initial Stage of the Pandemic: A Cross-Sectional Study. *Journal of Patient Safety* **18**(5), 499–506.

Benavides Morales JA and **López Peláez J** (2022) Factors associated with depression during lockdown in college students who sought psychological consultation. *The Journal of Mental Health Training, Education and Practice* **17**(4), 366–379.

Beymer MR, **Apostolou A**, **Smith CM**, **Paschane DM**, **Gomez SAQ**, **James TD**, **Bell AM**, **Santo T** and **Quartana PJ** (2023) Mental Health Outcomes Among American Indian and Alaska Native U.S. Army Soldiers: A Serial Cross-Sectional Analysis. *Military Medicine* **188**(7–8), e2292–e2299.

Bi K, **Yeoh D**, **Jiang Q**, **Wienk MNA** and **Chen S** (2023) Psychological distress and everyday discrimination among Chinese international students one year into COVID-19: a preregistered comparative study. *Anxiety, Stress, & Coping* **36**(6), 727–742.

Bilgi K, **Aytaş G**, **Karatoprak U**, **Kazancıoğlu R** and **Özçelik S** (2021) The Effects of Coronavirus Disease 2019 Outbreak on Medical Students. *Frontiers in Psychiatry* **12**, 637946.

Bismark M, **Scurrah K**, **Pascoe A**, **Willis K**, **Jain R** and **Smallwood N** (2022) Thoughts of suicide or self-harm among Australian healthcare workers during the COVID-19 pandemic. *Australian & New Zealand Journal of Psychiatry* **56**(12), 1555–1565.

Blay Benzaken Y, **Zohar S**, **Yuval K**, **Aizik-Reebs A**, **Gebremariam SG** and **Bernstein A** (2023) COVID-19 and Mental Health Among People Who Are Forcibly Displaced: The Role of Socioeconomic Insecurity. *Psychiatric Services* **74**(2), 158–165.

Bonello F, **Zammit D**, **Grech A**, **Camilleri V** and **Cremona R** (2021) Effect of COVID-19 pandemic on mental health hospital admissions: comparative population-based study. *BJPsych Open* **7**(5), e141.

Bountress KE, **Cusack SE**, **Conley AH**, **Aggen SH**, **The Spit For Science Working Group**, **Vassileva J**, **Dick DM** and **Amstadter AB** (2022) The COVID-19 pandemic impacts

psychiatric outcomes and alcohol use among college students. *European Journal of Psychotraumatology* **13**(1), 2022279.

Brady C, **Fenton C**, **Loughran O**, **Hayes B**, **Hennessy M**, **Higgins A**, **Leroi I**, **Shanagher D** and **McLoughlin DM** (2022) Nursing home staff mental health during the Covid-19 pandemic in the Republic of Ireland. *International Journal of Geriatric Psychiatry* **37**(1), gps.5648.

Brady C, **Fenton C**, **Loughran O**, **Hayes B**, **Hennessy M**, **Higgins A** and **McLoughlin DM** (2023) Dublin hospital workers' mental health during the peak of Ireland's COVID-19 pandemic. *Irish Journal of Medical Science (1971 -)* **192**(3), 1293–1302.

Brady C, **Shackleton E**, **Fenton C**, **Loughran O**, **Hayes B**, **Hennessy M**, **Higgins A**, **Leroi I**, **Shanagher D** and **McLoughlin DM** (2023) Worsening of mental health outcomes in nursing home staff during the COVID-19 pandemic in Ireland. *PLOS ONE* García-Iglesias JJ (ed.) **18**(9), e0291988.

Brasso C, **Cisotto M**, **Del Favero E**, **Giordano B**, **Villari V** and **Rocca P** (2023) Impact of COVID-19 pandemic on major depressive disorder in acute psychiatric inpatients. *Frontiers in Psychology* **14**, 1181832.

Bressington DT, **Cheung TCC**, **Lam SC**, **Suen LKP**, **Fong TKH**, **Ho HSW** and **Xiang Y-T** (2020) Association Between Depression, Health Beliefs, and Face Mask Use During the COVID-19 Pandemic. *Frontiers in Psychiatry* **11**, 571179.

Bukuluki P, **Wandiembe S**, **Kisaakye P**, **Besigwa S** and **Kasirye R** (2021) Suicidal Ideations and Attempts Among Adolescents in Kampala Urban Settlements in Uganda: A Case Study of Adolescents Receiving Care From the Uganda Youth Development Link. *Frontiers in Sociology* **6**, 646854.

Burke TA, **Bettis AH**, **Kudinova A**, **Thomas SA**, **Nesi J**, **Erguder L**, **MacPherson HA**, **Thompson E**, **Ammerman BA** and **Wolff JC** (2022) COVID-19-Specific Suicidal Thoughts and Behaviors in Psychiatrically Hospitalized Adolescents. *Child Psychiatry & Human Development* **53**(6), 1383–1390.

Bustos Villarroel A, **Pereda N** and **Suárez-Soto E** (2024) Mental Health and Suicidal Behaviour in Chilean Youth during the Covid-19 Pandemic. *Actas Españolas de Psiquiatría* **52**(4), 453–463.

Byeon H (2022) Prediction of adolescent suicidal ideation after the COVID-19 pandemic: A nationwide survey of a representative sample of Korea. *Frontiers in Pediatrics* **10**, 951439.

Cai H, **Li Z-L**, **He F**, **Li S-Y**, **Zhao Y-J**, **Zhang W-Y**, **Zhang Y**, **Su Z**, **Jackson T** and **Xiang Y-T** (2023) Suicide ideation and anhedonia among clinically stable adolescents with the recurrent depressive disorder during the COVID-19 pandemic: A network perspective. *Journal of Affective Disorders* **324**, 317–324.

Cai Q, **Feng H**, **Huang J**, **Wang M**, **Wang Q**, **Lu X**, **Xie Y**, **Wang X**, **Liu Z**, **Hou B**, **Ouyang K**, **Pan J**, **Li Q**, **Fu B**, **Deng Y** and **Liu Y** (2020) The mental health of frontline and non-frontline medical workers during the coronavirus disease 2019 (COVID-19) outbreak in China: A case-control study. *Journal of Affective Disorders* **275**, 210–215.

Carlos KM, **Ahmadi H**, **Uban KA** and **Riis JL** (2023) Behavioral and psychosocial factors related to mental distress among medical students. *Frontiers in Public Health* **11**, 1225254.

Casas Muñoz A, **Velasco Rojano ÁEE**, **Rodríguez Caballero A**, **Prado Solé E** and **Alvarez MG** (2024) Suicidal behavior in Mexican adolescents: Comparative analysis among geographic regions of the country. *Acta Pediátrica de México* **45**(1S), S47–S53.

Chakrabarti S (2021) Mental Health in Hospitalised COVID 19 Patients in Quarantine During Second Wave in a South Indian Private Teaching Hospital. *Journal of Multidisciplinary Healthcare* **Volume 14**, 2777–2789.

Chen M-Y, **Chen P**, **An F-R**, **Sha S**, **Feng Y**, **Su Z**, **Cheung T**, **Ungvari GS**, **Ng CH**, **Zhang L** and **Xiang Y-T** (2024) Depression, anxiety and suicidality among Chinese mental health professionals immediately after China's dynamic zero-COVID policy: A network perspective. *Journal of Affective Disorders* **352**, 153–162.

Chen X, **Dai Z**, **Fu J**, **Si M**, **Jing S**, **Wu Y**, **Wang H**, **Huang Y**, **Cui D**, **Qu Y** and **Su X** (2023) Suicidal ideation and associated risk factors among COVID-19 patients who recovered from the first wave of the pandemic in Wuhan, China. *QJM: An International Journal of Medicine* **116**(7), 509–517.

Chen Y, **Zhu L**, **Fang Z**, **Wu N**, **Du M**, **Jiang M**, **Wang J**, **Yao Y** and **Zhou C** (2021) The Association of Suicidal Ideation With Family Characteristics and Social Support of the First Batch of Students Returning to a College During the COVID-19 Epidemic Period: A Cross Sectional Study in China. *Frontiers in Psychiatry* **12**, 653245.

Chen Y, **Wang J**, **Lin H**, **Richards M**, **Yang X**, **Wang T**, **Chen X** and **Fu C** (2023) Network structure of emotional and behavioral problems, loneliness, and suicidal thoughts in adolescents at the school closure and reopening stage in China. *Translational Pediatrics* **12**(7), 1373–1385.

Chen Y, **Ke X**, **Liu J**, **Du J**, **Zhang J**, **Jiang X**, **Zhou T** and **Xiao X** (2024) Trends and factors influencing the mental health of college students in the post-pandemic: four consecutive cross-sectional surveys. *Frontiers in Psychology* **15**, 1387983.

Chen Z, **Fedina L**, **DeVylder J**, **Lemieux CM**, **King C** and **Abbott K** (2023) Pandemic financial hardships and suicide risk in a nationally representative sample of young adults. *Social Work in Mental Health* **21**(4), 422–436.

Cheung T, **Lam SC**, **Lee PH**, **Xiang YT**, **Yip PSF**, and **The International Research Collaboration on COVID-19** (2021) Global Imperative of Suicidal Ideation in 10 Countries Amid the COVID-19 Pandemic. *Frontiers in Psychiatry* **11**, 588781.

Chodkiewicz J, **Miniszewska J**, **Krajewska E** and **Biliński P** (2021) Mental Health during the Second Wave of the COVID-19 Pandemic—Polish Studies. *International Journal of Environmental Research and Public Health* **18**(7), 3423.

Choi KS, **Kim S**, **Kim B-H**, **Jeon HJ**, **Kim J-H**, **Jang JH** and **Jeong B** (2021) Deep graph neural network-based prediction of acute suicidal ideation in young adults. *Scientific Reports* **11**(1), 15828.

Crisol-Deza D, **Poma-Ramírez D**, **Pacherres-López A**, **Noriega-Baella C**, **Villanueva-Zúñiga L**, **Salvador-Carrillo J** and **Huarcaya-Victoria J** (2023) Factors associated with suicidal ideation among medical students during the initial phase of the COVID-19 pandemic in Peru: A multicenter study. *Death Studies* **47**(2), 183–191.

Czeisler MÉ, **Wiley JF**, **Facer-Childs ER**, **Robbins R**, **Weaver MD**, **Barger LK**, **Czeisler CA**, **Howard ME** and **Rajaratnam SMW** (2021) Mental health, substance use, and suicidal ideation during a prolonged COVID-19-related lockdown in a region with low SARS-CoV-2 prevalence. *Journal of Psychiatric Research* **140**, 533–544.

Dale R, **Jesser A**, **Pieh C**, **O'Rourke T**, **Probst T** and **Humer E** (2023) Mental health burden of high school students, and suggestions for psychosocial support, 1.5 years into the COVID-19 pandemic in Austria. *European Child & Adolescent Psychiatry* **32**(6), 1015–1024.

Daly Z, **Slemon A**, **Richardson CG**, **Salway T**, **McAuliffe C**, **Gadermann AM**, **Thomson KC**, **Hirani S** and **Jenkins EK** (2021) Associations between periods of COVID-19 quarantine and mental health in Canada. *Psychiatry Research* **295**, 113631.

Danzo S, **Kuklinski MR**, **Sterling SA**, **Beck A**, **Braciszewski JM**, **Boggs J**, **Briney JS**, **Charvat-Aguilar N**, **Eisenberg N**, **Kaffl A**, **Kline-Simon A**, **Loree AM**, **Lyons VH**, **Morse EF**, **Morrison KM**, **Negusse R** and **Scheuer H** (2024) Anxiety, depression, and suicidal ideation among early adolescents during the COVID-19 pandemic. *Journal of Adolescence* **96**(6), 1379–1387.

De Chiara L, **Angeletti G**, **Anibaldi G**, **Chetoni C**, **Gualtieri F**, **Forcina F**, **Bargagna P**, **Kotzalidis GD**, **Callovini T**, **Bonito M**, **Koukopoulos AE** and **Simonetti A** (2022) The Impact of the COVID-19 Pandemic on Postpartum Maternal Mental Health. *Journal of Personalized Medicine* **13**(1), 56.

De La Vega Sánchez D, **Irigoyen-Otiñano M**, **Carballo JJ**, **Guija JA** and **Giner L** (2023) Suicidal thoughts and burnout among physicians during the first wave of the COVID-19 pandemic in Spain. *Psychiatry Research* **321**, 115057.

De Miquel C, **Domènech-Abella J**, **Felez-Nobrega M**, **Cristóbal-Narváez P**, **Mortier P**, **Vilagut G**, **Alonso J**, **Olaya B** and **Haro JM** (2022) The Mental Health of Employees with Job Loss and Income Loss during the COVID-19 Pandemic: The Mediating Role of Perceived Financial Stress. *International Journal of Environmental Research and Public Health* **19**(6), 3158.

Delgadillo J, **Budimir S**, **Barkham M**, **Humer E**, **Pieh C** and **Probst T** (2023) A Bayesian network analysis of psychosocial risk and protective factors for suicidal ideation. *Frontiers in Public Health* **11**, 1010264.

Deng A-P, **Wang C**, **Cai J**, **Deng Z-Y**, **Mu Y-F**, **Song H-J**, **Meng Y-J**, **Meng X-D**, **Huang X-H**, **Zhang L**, **Huang Y**, **Zhang W**, **Chen J** and **Ran M-S** (2023) Effects of internet addiction and academic satisfaction on mental health among college students after the lifting of COVID-19 restrictions in China. *Frontiers in Psychiatry* **14**, 1243619.

DeVylder J, **Zhou S** and **Oh H** (2021) Suicide attempts among college students hospitalized for COVID-19. *Journal of Affective Disorders* **294**, 241–244.

Dogan-Sander E, **Kohls E**, **Baldofski S** and **Rummel-Kluge C** (2021) More Depressive Symptoms, Alcohol and Drug Consumption: Increase in Mental Health Symptoms Among University Students After One Year of the COVID-19 Pandemic. *Frontiers in Psychiatry* **12**, 790974.

Domínguez-González AD, **Guzmán-Valdivia G**, **Ángeles-Téllez FS**, **Manjarrez-Ángeles MA** and **Secín-Diep R** (2022) Depression and suicidal ideation in Mexican medical students during COVID-19 outbreak. A longitudinal study. *Heliyon* **8**(2), e08851.

Dooley N, **Power E**, **Healy H**, **Cotter D** and **Cannon M** (2024) Mental health of Irish adolescents following the COVID-19 pandemic: results from a population-based cross-sectional survey. *Irish Journal of Psychological Medicine* **41**(4), 430–438.

Doukas A, **DePierro J**, **Starkweather S**, **Sharma V**, **Marin DB** and **Charney DS** (2023) Symptom characteristics of health care workers seeking outpatient psychiatric care during the COVID-19 pandemic. *American Journal of Industrial Medicine* **66**(6), 500–509.

Drucker A, **Levi-Belz Y** and **Hamdan S** (2023) Depression, Complicated Grief, and Suicide Ideation Following Bereavement During the COVID-19 Pandemic. *OMEGA - Journal of Death and Dying*, 00302228231186361.

Dumont R, **Lorthe E**, **Richard V**, **Loizeau A**, **Fernandez G**, **De Ridder D**, **Pennacchio F**, **Lamour J**, **Zaballa M-E**, **Baysson H**, **Posfay-Barbe KM**, **Barbe RP**, **Stringhini S** and **Guessous I** (2024) Prevalence of and risk factors for suicidal ideation in adolescents during the COVID-19 pandemic: a cross-sectional study. *Swiss Medical Weekly* **154**(4), 3461.

Efstathiou V, **Michopoulos I**, **Yotsidi V**, **Smyrnis N**, **Zompola C**, **Papadopoulou A**, **Pomini V**, **Papadopoulou M**, **Tsigkaropoulou E**, **Tsivgoulis G**, **Douzenis A** and **Gournellis R** (2021) Does suicidal ideation increase during the second COVID-19 lockdown? *Psychiatry Research* **301**, 113990.

Efstathiou V, **Papadopoulou A**, **Pomini V**, **Yotsidi V**, **Kalemi G**, **Chatzimichail K**, **Michopoulos I**, **Kaparoudaki A**, **Papadopoulou M**, **Smyrnis N**, **Douzenis A** and **Gournellis R** (2022) A one-year longitudinal study on suicidal ideation, depression and anxiety during the COVID-19 pandemic. *Asian Journal of Psychiatry* **73**, 103175.

El Frenn Y, **Hallit S**, **Obeid S** and **Soufia M** (2023) Association of the time spent on social media news with depression and suicidal ideation among a sample of Lebanese adults during the COVID-19 pandemic and the Lebanese economic crisis. *Current Psychology* **42**(24), 20638–20650.

Eleftheriou A, **Rokou A**, **Arvaniti A**, **Nena E** and **Steiropoulos P** (2021) Sleep Quality and Mental Health of Medical Students in Greece During the COVID-19 Pandemic. *Frontiers in Public Health* **9**, 775374.

Elhadi M, **Buzreg A**, **Bouhuwaish A**, **Khaled Ala**, **Alhadi A**, **Msherghi A**, **Alsoufi A**, **Alameen H**, **Biala M**, **Elgherwi A**, **Elkhafeefi F**, **Elmabrouk A**, **Abdulmalik A**, **Alhaddad S**, **Elgzairi M** and **Khaled Ahmed** (2020) Psychological Impact of the Civil War and COVID-19 on Libyan Medical Students: A Cross-Sectional Study. *Frontiers in Psychology* **11**, 570435.

Elhadi M, **Msherghi A**, **Khaled A**, **Alsoufi A**, **Alhadi A**, **Kareem A**, **Ashini A**, **Alsharif T**, **Alhodiri A**, **Altaeb E**, **Hamed M**, **Itrunbah A**, **Mohmmed S**, **Alameen H**, **Idheiraj H**, **Shuwayyah A**, **Alhudhairy S**, **Alansari A**, **Abraheem W**, **Akl H**, **Nagib T**, **Almugaddami A**, **Aljameel B**, **Muamr S**, **Alsuwiyah S**, **Alsghair A**, **Soula E**, **Buzreg A**, **Alagelli F**, **Aldireewi A**, **Bareem A**, **Alshareea E**, **Gemberlo A** and **Zaid A** (2022) Impact of lockdown due to the COVID-19 pandemic on mental health among the Libyan population. *PLOS ONE* Hossain S (ed.) **17**(4), e0267426.

Erjavac BW, **Rolin AI**, **Gondy K**, **Cranford JA**, **Shobassy A**, **Biermann BJ**, **Rogers AJ** and **Hong V** (2023) Impact of the COVID-19 Pandemic on Children and Adolescents Presenting With a Psychiatric Emergency. *Clinical Pediatrics* **62**(3), 241–250.

Ernst M, **Beutel ME** and **Brähler E** (2022) Cancer as a risk factor for distress and its interactions with sociodemographic variables in the context of the first wave of the COVID-19 pandemic in Germany. *Scientific Reports* **12**(1), 2021.

Escobar-Agreda S, **Romero Albino Z**, **Contreras PJ** and **Cuba-Fuentes MS** (2023) Complicated grief and its relationship with anxiety, depression, and suicidal ideation in older adults in the context of the COVID-19 pandemic in Peru: a cross-sectional analysis. *BMC Psychiatry* **23**(1), 908.

Essadek A, **Gressier F**, **Krebs T**, **Corruble E**, **Falissard B** and **Rabeyron T** (2022) Assessment of mental health of university students faced with different lockdowns during the coronavirus pandemic, a repeated cross-sectional study. *European Journal of Psychotraumatology* **13**(2), 2141510.

Essadek A, **Shadili G**, **Bergami Goulart Barbosa P**, **Assous A**, **Widart F**, **Payan S**, **Rabeyron T**, **Corruble E**, **Falissard B** and **Gressier F** (2023) Precarious Young Adults' Mental Health during the Pandemic: The Major Impact of Food Insecurity Independently of COVID-19 Diagnosis. *Nutrients* **15**(14), 3260.

Every-Palmer S, **Jenkins M**, **Gendall P**, **Hoek J**, **Beaglehole B**, **Bell C**, **Williman J**, **Rapsey C** and **Stanley J** (2020) Psychological distress, anxiety, family violence, suicidality, and wellbeing in New Zealand during the COVID-19 lockdown: A cross-sectional study. *PLOS ONE* Francis JM (ed.) **15**(11), e0241658.

Fadipe B, **Oshodi YO**, **Umeh C**, **Aikomo K**, **Ajirotutu OF**, **Kajero J**, **Fashanu C**, **Adeoye AA**, **Coker AO**, **Sokunbi M**, **Nyamali VO**, **Ajomale T**, **Gbadebo A**, **Oni A**, **Keshinro AO**, **Ngozi Ejiegbu TA**, **Bowale A**, **Akase IE**, **Mutiu B**, **Adebayo B**, **Abdus-Salam I**, **Bode C** and **Osibogun A** (2021) Psychosocial health effects of Covid-19 infection on persons in treatment centers in Lagos, Nigeria. *Brain, Behavior, & Immunity - Health* **16**, 100284.

Ferrando SJ, **Klepacz L**, **Lynch S**, **Shahar S**, **Dornbush R**, **Smiley A**, **Miller I**, **Tavakkoli M**, **Regan J** and **Bartell A** (2021) Psychiatric emergencies during the height of the COVID-19 pandemic in the suburban New York City area. *Journal of Psychiatric Research* **136**, 552–559.

Ferro MA, **Chan CKY**, **Browne DT**, **Colman I**, **Dubin JA** and **Duncan L** (2024) Suicidal Ideation and Attempts Among Youth With Physical-Mental Comorbidity in Canada: Proposal for an Epidemiological Study. *JMIR Research Protocols* **13**, e57103.

Fisch CT, **Sheffer Y**, **Lazarov A**, **Liu J**, **Lewis-Fernández R**, **Dixon LB**, **Neria Y** and **Amsalem D** (2025) Suicidal ideation among young US essential workers during the COVID-19 pandemic: A longitudinal study. *Journal of Affective Disorders* **382**, 366–372.

Fong TCT, **Ho RTH** and **Yip PSF** (2023) Psychometric properties of the Patient Health Questionnaire-4 among Hong Kong young adults in 2021: Associations with meaning in life and suicidal ideation. *Frontiers in Psychiatry* **14**, 1138755.

Fountoulakis KN, **Apostolidou MK**, **Atsiova MB**, **Filippidou AK**, **Florou AK**, **Gousiou DS**, **Katsara AR**, **Mantzari SN**, **Padouva-Markoulaki M**, **Papatriantafyllou EI**, **Sacharidi PI**, **Tonia AI**, **Tsagalidou EG**, **Zymara VP**, **Prezerakos PE**, **Koupidis SA**, **Fountoulakis NK**, **Konsta A**, **Tsapakis EM**, **Theodorakis PN** and **Mossialos E** (2022) Mental health and conspirasism in health care professionals during the spring 2020 COVID-19 lockdown in Greece. *Acta Neuropsychiatrica* **34**(3), 132–147.

Frajerman A (2023) Mental Health of Health and Medical Students during the COVID-19 Pandemic: National Studies. *European Psychiatry* **66**(S1). 2023/07/19 edn. Cambridge University Press, S16–S16.

Frajerman A, **Chaumette B**, **Krebs M-O** and **Morvan Y** (2022) Mental health in medical, dental and pharmacy students: A cross-sectional study. *Journal of Affective Disorders Reports* **10**, 100404.

Frey LM, **Venugopal D** and **Dev VS** (2024) Prevalence and predictors of suicide ideation among university and high-school students during India's 2nd wave of the COVID-19 pandemic. *PLOS ONE* Alemayehu Y (ed.) **19**(12), e0311403.

Gadermann AC, **Thomson KC**, **Richardson CG**, **Gagné M**, **McAuliffe C**, **Hirani S** and **Jenkins E** (2021) Examining the impacts of the COVID-19 pandemic on family mental health in Canada: findings from a national cross-sectional study. *BMJ Open* **11**(1), e042871.

Gainza Perez MA, **Woloshchuk CJ**, **Rodríguez-Crespo A**, **Louden JE** and **Cooper TV** (2022) Influence of suicidality on adult perceptions of COVID-19 risk and guideline adherence. *Journal of Affective Disorders* **308**, 27–30.

Galletta MAK, **Oliveira AMDSS**, **Albertini JGL**, **Benute GG**, **Peres SV**, **Brizot MDL** and **Francisco RPV** (2022) Postpartum depressive symptoms of Brazilian women during the COVID-19 pandemic measured by the Edinburgh Postnatal Depression Scale. *Journal of Affective Disorders* **296**, 577–586.

Gao L, **Wang L-L**, **Yang R**, **Yang X-J** and **Zhou S-J** (2023) Anxiety, Depression, Perceived Stress, and Burnout Among Chinese Researchers: A Cross-Sectional Nationwide Study. *Psychology Research and Behavior Management* **Volume 16**, 4209–4220.

Geda N, **Feng C** and **Peters B** (2022) Suicidal ideation among Canadian adults during the COVID-19 pandemic: the role of psychosocial factors and substance use behaviours. *BMC Psychiatry* **22**(1), 711.

Gelezelyte O, **Kazlauskas E**, **Brailovskaia J**, **Margraf J** and **Truskauskaite-Kuneviciene I** (2022) Suicidal ideation in university students in Lithuania amid the COVID-19 pandemic: A

prospective study with pre-pandemic measures. *Death Studies* **46**(10), 2395–2403.

Généreux M and **Landaverde E** (2022) Psychological symptoms associated with self-reported events of COVID-19 contact, symptoms, or diagnosis: a large community-based survey among adults in Quebec, Canada. *Canadian Journal of Public Health* **113**(3), 394–404.

Genis-Mendoza AD, **Martínez-Magaña JJ**, **López-Narváez ML**, **González-Castro TB**, **Juárez-Rojop IE**, **Nicolini H**, **Tovilla-Zárate CA** and **Castillo-Avila RG** (2021) Mental Health Problems Due to Social Isolation During the COVID-19 Pandemic in a Mexican Population. *Frontiers in Public Health* **9**, 703450.

Gesi C, **Cafaro R**, **Achilli F**, **Boscacci M**, **Cerioli M**, **Cirnigliaro G**, **Loupakis F**, **Di Maio M** and **Dell'Osso B** (2024) The relationship among posttraumatic stress disorder, posttraumatic growth, and suicidal ideation among Italian healthcare workers during the first wave of COVID-19 pandemic. *CNS Spectrums* **29**(1), 60–64.

Giannakopoulos G, **Zaravinos-Tsakos F**, **Pilafa E**, **Sourander A** and **Kolaitis G** (2025) Self-injurious behavior in Greek adolescents: the role of mental health problems and COVID-19 trauma. *BMC Psychiatry* **25**(1), 579.

Gómez Delgado G, **Ponce Rojo A**, **Ramírez Mireles JE**, **Carmona-Moreno FDJ**, **Flores Salcedo CC** and **Hernández Romero AM** (2024) Suicide Risk Factors in High School Students. *International Journal of Environmental Research and Public Health* **21**(8), 1055.

Gómez PM, **Lázaro-Pérez C**, **Martínez-López JÁ** and **Gómez-Galán J** (2023) Suicidal Ideation in Undergraduate Students of Social Work: A Quantitative Study. *Journal of New Approaches in Educational Research* **12**(2), 360–377.

Gómez-García JA, **Rivera-Rivera L**, **Astudillo-García CI**, **Castillo-Castillo LE**, **Morales-Chainé S** and **Tejadilla-Orozco DI** (2023) Determinantes sociales asociados con ideación suicida durante la pandemia por Covid-19 en México. *Salud Pública de México* **65**(1, ene-feb), 1–9.

Gonzalez Mendez MJ, **Ma L**, **Alvarado R**, **Ramirez J**, **Xu K-P**, **Xu H-F**, **Zhang S-K**, **Bangura MS**, **Yang Y**, **Yu Y-Q**, **Zhang X**, **Wang W**, **Gu X**, **Li L**, **Salah DS** and **Qiao Y** (2022) A Multi-Center Study on the Negative Psychological Impact and Associated Factors in Chinese Healthcare Workers 1 Year After the COVID-19 Initial Outbreak. *International Journal of Public Health* **67**, 1604979.

Goodman ML, **Molldrem S**, **Elliott A**, **Robertson D** and **Keiser P** (2023) Long COVID and mental health correlates: a new chronic condition fits existing patterns. *Health Psychology and Behavioral Medicine* **11**(1), 2164498.

Goodwill JR and **Ajibewa TA** (2024) A Mixed Methods Analysis of Long COVID Symptoms in Black Americans: Examining Physical and Mental Health Outcomes. *Journal of Racial and Ethnic Health Disparities*. Epub ahead of print 24 September 2024. DOI: 10.1007/s40615-024-02170-2.

Goodwill JR and **Taylor HO** (n.d.) Measuring Whether Gratitude and Loneliness Mediate the Link Between Non-organizational Religiosity and Suicidal Ideation: Evidence From Black Adults

During COVID-19.

Goodyear T, **Slemon A**, **Richardson C**, **Gadermann A**, **Salway T**, **Dhari S**, **Knight R** and **Jenkins E** (2021) Increases in Alcohol and Cannabis Use Associated with Deteriorating Mental Health among LGBTQ2+ Adults in the Context of COVID-19: A Repeated Cross-Sectional Study in Canada, 2020–2021. *International Journal of Environmental Research and Public Health* **18**(22), 12155.

Gouin J-P, **MacNeil S**, **De La Torre-Luque A**, **Chartrand E**, **Chadi N**, **Rouquette A**, **Boivin M**, **Côté S** and **Geoffroy M-C** (2023) Depression, anxiety, and suicidal ideation in a population-based cohort of young adults before and during the first 12 months of the COVID-19 pandemic in Canada. *Canadian Journal of Public Health* **114**(3), 368–377.

Gournellis R and **Efstathiou V** (2021) The impact of the COVID-19 Pandemic on the Greek population: Suicidal ideation during the first and second lockdown. *Psychiatriki* **32**(4), 267–270.

Gratz KL, **Mann AJD** and **Tull MT** (2021) Suicidal ideation among university students during the COVID-19 pandemic: Identifying at-risk subgroups. *Psychiatry Research* **302**, 114034.

Guerrero MD and **Barnes JD** (n.d.) Profiles of mental health and their association with negative impacts and suicidal ideation during the COVID-19 pandemic: A Canadian perspective.

Guo B-C, **Chen Y-J**, **Huang W-Y**, **Lin M-J** and **Wu H-P** (2023) Psychological disorders and suicide attempts in youths during the pre-COVID and post-COVID era in a Taiwan pediatric emergency department. *Frontiers in Psychology* **14**, 1281806.

Haas LM, **Silveira JTFD**, **Rodrigues GDF**, **Duarte MDQ** and **Trentini CM** (2024) Psychosocial correlates of risk for suicidal ideation: the COVID-19 pandemic as a magnifying glass. *Estudos de Psicologia (Campinas)* **41**, e230005.

Hall BJ, **Li G**, **Chen W**, **Shelley D** and **Tang W** (2023) Prevalence of depression, anxiety, and suicidal ideation during the Shanghai 2022 Lockdown: A cross-sectional study. *Journal of Affective Disorders* **330**, 283–290.

Hamdan S, **Guz T** and **Zalsman G** (2025) The Clinical Sequelae of the COVID-19 Pandemic: Loneliness, Depression, Excessive Alcohol Use, Social Media Addiction, and Risk for Suicide Ideation. *Archives of Suicide Research* **29**(1), 238–251.

Hamm ME, **Brown PJ**, **Karp JF**, **Lenard E**, **Cameron F**, **Dawdani A**, **Lavretsky H**, **Miller JP**, **Mulsant BH**, **Pham VT**, **Reynolds CF**, **Roose SP** and **Lenze EJ** (2020) Experiences of American Older Adults with Pre-existing Depression During the Beginnings of the COVID-19 Pandemic: A Multicity, Mixed-Methods Study. *The American Journal of Geriatric Psychiatry* **28**(9), 924–932.

Han K-M, **Lee SM**, **Hong M**, **Kim S-J**, **Sohn S**, **Choi Y-K**, **Hyun J**, **Kim H**, **Lee J-S**, **Lee SH**, **Lee Y-R** and **Paik J-W** (2023) COVID-19 Pandemic-Related Job Loss Impacts on Mental Health in South Korea. *Psychiatry Investigation* **20**(8), 730–739.

Hano D, **An A**, **Nghiem J**, **Koh E**, **Tripathi V**, **Wirtshafter R** and **Green C** (2025) An

Investigation of Adolescent Mental Health In a New York City Cohort Before and During the COVID-19 Pandemic in the Primary Care Setting. *Maternal and Child Health Journal* **29**(2), 156–163.

Hassan F, **Liu L** and **Feng C** (2025) Double Burden of Distress: Exploring the Joint Associations of Loneliness and Financial Strain with Suicidal Ideation During the COVID-19 Pandemic in Canada. *International Journal of Environmental Research and Public Health* **22**(5), 682.

He Z, **Lei Q**, **Hu X**, **Xiong M**, **Liu J**, **Wen J**, **Shi X** and **Wang Z** (2023) Moral injury and suicidal ideation among health professionals: The role of mental health. *Journal of Affective Disorders* **333**, 58–64.

Höller I and **Forkmann T** (2022) Ambivalent heroism? – Psychological burden and suicidal ideation among nurses during the Covid-19 pandemic. *Nursing Open* **9**(1), 785–800.

Hong S, **Ai M**, **Xu X**, **Wang W**, **Chen J**, **Zhang Q**, **Wang L** and **Kuang L** (2021) Immediate psychological impact on nurses working at 42 government-designated hospitals during COVID-19 outbreak in China: A cross-sectional study. *Nursing Outlook* **69**(1), 6–12.

Hou T, **Mao X**, **Shao X**, **Liu F**, **Dong W** and **Cai W** (2021) Suicidality and Its Associated Factors Among Students in Rural China During COVID-19 Pandemic: A Comparative Study of Left-Behind and Non-Left-Behind Children. *Frontiers in Psychiatry* **12**, 708305.

Howard KJ, **Leong C**, **Chambless S**, **Grigsby TJ**, **Cordaro M**, **Perrotte JK** and **Howard JT** (2022) Major Depression in Postpartum Women during the COVID-19 Pandemic: Can Social Support Buffer Psychosocial Risks and Substance Use? *International Journal of Environmental Research and Public Health* **19**(23), 15748.

Huang S, **Wang D**, **Zhao J**, **Chen H**, **Ma Z**, **Pan Y**, **Liu X** and **Fan F** (2022) Changes in suicidal ideation and related influential factors in college students during the COVID-19 lockdown in China. *Psychiatry Research* **314**, 114653.

Husky MM, **Léon C**, **Du Roscoät E** and **Vasiliadis H-M** (2024) Prevalence of suicidal thoughts and behaviors among young adults between 2000 and 2021: Results from six national representative surveys in France. *Psychiatry Research* **333**, 115763.

Husky MM, **Pic O**, **Callahan S** and **Navarro-Mateu F** (2024) Twelve-month suicidal ideation, incidence and persistence among college students pre-pandemic and during the pandemic: A longitudinal study. *Psychiatry Research* **331**, 115669.

Ibeziako P, **Kaufman K**, **Scheer KN** and **Sideridis G** (2022) Pediatric Mental Health Presentations and Boarding: First Year of the COVID-19 Pandemic. *Hospital Pediatrics* **12**(9), 751–760.

Ide K, **Suda A**, **Yoshimi A**, **Fujita J**, **Nomoto M**, **Miyauchi M**, **Roppongi T**, **Hishimoto A**, **Odawara T** and **Asami T** (2025) General and event-related psychological stress, and suicidal ideation among hospital workers during the coronavirus disease 2019 pandemic: Findings from the third wave of repeated cross-sectional studies. *Psychiatry and Clinical Neurosciences Reports* **4**(3), e70157.

Iftene F, **Milev R**, **Farcas A**, **Squires S**, **Smirnova D** and **Fountoulakis KN** (2022) COVID-19

Pandemic: The Impact of COVID-19 on Mental Health and Life Habits in the Canadian Population. *Frontiers in Psychiatry* **13**, 871119.

Jadir DS and **Anderson-Carpenter KD** (2022) Substance use, racial/ethnic identity, and suicidal ideation during COVID-19 lockdown in an international adult sample. *Journal of Psychiatric Research* **155**, 443–450.

Jaramillo N, **Bonilla GS**, **Piña-Watson B**, **Manzo G**, **Garcia A**, **Sanchez M** and **Meza JI** (2025) The role of racial bias and pathways to self-harm outcomes for Mexican-descent college students during the COVID-19 pandemic. *American Journal of Orthopsychiatry* **95**(2), 166–175.

Jeong H, **Yim HW**, **Lee S-Y** and **Jung DY** (2023) Impact of the COVID-19 Pandemic on Gender Differences in Depression Based on National Representative Data. *Journal of Korean Medical Science* **38**(6), e36.

Jilowa CS, **Meena PS**, **Jain M**, **Prakash P** and **Tak P** (2022) Mental Health Status among the Quarantined Population during COVID-19 Pandemic: A Cross-sectional Study from Western Rajasthan. *JOURNAL OF CLINICAL AND DIAGNOSTIC RESEARCH*. Epub ahead of print 2022. DOI: 10.7860/JCDR/2022/51765.16308.

John A, **Lee SC**, **Solomon S**, **Crepaz-Keay D**, **McDaid S**, **Morton A**, **Davidson G**, **Van Bortel T** and **Kousoulis AA** (2021) Loneliness, coping, suicidal thoughts and self-harm during the COVID-19 pandemic: a repeat cross-sectional UK population survey. *BMJ Open* **11**(12), e048123.

Jones LB, **Vereschagin M**, **Wang AY**, **Munthali RJ**, **Pei J**, **Richardson CG**, **Halli P**, **Xie H**, **Rush B**, **Yatham L**, **Gadermann AM**, **Pendakur K**, **Prescivalli AP**, **Munro L**, **Bruffaerts R**, **Auerbach RP**, **Mortier P** and **Vigo DV** (2023) Suicidal Ideation Amongst University Students During the COVID-19 Pandemic: Time Trends and Risk Factors. *The Canadian Journal of Psychiatry* **68**(7), 531–546.

Joshi SS, **Stankovic I**, **Demirkiran A**, **Haugaa K**, **Maurovich-Horvat P**, **Popescu BA**, **Cosyns B**, **Edvardsen T**, **Petersen SE**, **Carvalho RF**, **Cameli M** and **Dweck MR** (2022) EACVI survey on burnout amongst cardiac imaging specialists during the 2019 coronavirus disease pandemic. *European Heart Journal - Cardiovascular Imaging* **23**(4), 441–446.

Jupina M, **Sidle MW** and **Rehmeyer Caudill CJ** (2022) Medical student mental health during the COVID-19 pandemic. *The Clinical Teacher* **19**(5), e13518.

Kabir H, **Chowdhury SR**, **Roy AK**, **Chowdhury SA**, **Islam MdN**, **Chomon RJ**, **Akter M** and **Hossain A** (2023) Association of workplace bullying and burnout with nurses' suicidal ideation in Bangladesh. *Scientific Reports* **13**(1), 14641.

Kaggwa MM, **Arinaitwe I**, **Nduhuura E**, **Muwanguzi M**, **Kajjimu J**, **Kule M**, **Ajuna N**, **Machacha I**, **Nkola R**, **Najjuka SM**, **Wamala NK**, **Bongomin F**, **Griffiths MD**, **Rukundo GZ** and **Mamun MA** (2022) Prevalence and Factors Associated With Depression and Suicidal Ideation During the COVID-19 Pandemic Among University Students in Uganda: A Cross-Sectional Study. *Frontiers in Psychiatry* **13**, 842466.

Kaggwa MM, **Arinaitwe I**, **Muwanguzi M**, **Nduhuura E**, **Kajjimu J**, **Kule M**, **Najjuka SM**, **Nkola R**, **Ajuna N**, **Wamala NK**, **Machacha I**, **Mamun MA**, **Ho CS-H**, **Griffiths MD** and **Rukundo GZ** (2022) Suicidal behaviours among Ugandan university students: a cross-sectional study. *BMC Psychiatry* **22**(1), 234.

Kang J, **Jang S** and **Kim H** (2023) What differentiates suicidal ideation from depressive symptoms amongst Korean Young Adults aged 20-39 years? In Review. Available at: https://www.researchsquare.com/article/rs-3524995/v1 (accessed 15 August 2025).

Kaparounaki CK, **Patsali ME**, **Mousa D-PV**, **Papadopoulou EVK**, **Papadopoulou KKK** and **Fountoulakis KN** (2020) University students' mental health amidst the COVID-19 quarantine in Greece. *Psychiatry Research* **290**, 113111.

Keshavarzi F, **Megan C**, **Sin CK**, **Wei CC**, **Chun C** and **Yin CWY** (n.d.) THE IMPACT OF COVID-19 PANDEMIC ON THE MENTAL HEALTH OF COLLEGE AND UNIVERSITY STUDENTS. **22**.

Keyworth C, **Quinlivan L**, **Leather JZ**, **O'Connor RC** and **Armitage CJ** (2022) The association between COVID-19-related fear and reported self-harm in a national survey of people with a lifetime history of self-harm. *BMC Psychiatry* **22**(1), 68.

Kholmogorova AB, **Rakhmanina AA**, **Suroegina AY**, **Mikita OY**, **Petrikov SS** and **Roy AP** (2021) Mental Health and Professional Burnout among Residents During the Covid-19 Pandemic: Situational and Psychological Factors. *Counseling Psychology and Psychotherapy* **29**(2), 9–47.

Killgore WDS, **Cloonan SA**, **Taylor EC**, **Allbright MC** and **Dailey NS** (2020) Trends in suicidal ideation over the first three months of COVID-19 lockdowns. *Psychiatry Research* **293**, 113390.

Killgore WDS, **Cloonan SA**, **Taylor EC** and **Dailey NS** (2021) Mental Health During the First Weeks of the COVID-19 Pandemic in the United States. *Frontiers in Psychiatry* **12**, 561898.

Kim AW (2024) Psychosocial stress, adult suicidal ideation, and the mediating effect of poor sleep quality during the COVID -19 pandemic in South Africa. *American Journal of Human Biology* **36**(3), e24038.

Kim E-Y, **Oh C**, **Sung H-J** and **Kim J** (2022) Changes in Daily Life, Physical Activity, GAD, Depression, and Personal Hygiene of Adolescents in South Korea Due to the COVID-19. *Healthcare* **10**(10), 1881.

Kim J, **Hoskinson C**, **Nichols E**, **Illapperuma-Wood C**, **Fischer A**, **Ben-Umeh K** and **Wilson F** (2025) COVID-19 and risk of self-harm, suicidal ideation, and poisoning in children and adolescents. *Annals of Medicine* **57**(1), 2516698.

Kim K, **Zhou Z**, **Ren X**, **Bu X**, **Jia X** and **Shao Q** (2024) Physical activity and mental health trends in Korean adolescents: Analyzing the impact of the COVID-19 pandemic from 2018 to 2022. *Open Medicine* **19**(1), 20240978.

Kim SY, **Kim H-R**, **Park B** and **Choi HG** (2021) Comparison of Stress and Suicide-Related

Behaviors Among Korean Youths Before and During the COVID-19 Pandemic. *JAMA Network Open* **4**(12), e2136137.

Kirič B, **Leben Novak L**, **Lušicky P** and **Drobnič Radobuljac M** (2022) Suicidal Behavior in Emergency Child and Adolescent Psychiatric Service Users Before and During the 16 Months of the COVID-19 Pandemic. *Frontiers in Psychiatry* **13**, 893040.

Kleinhendler-Lustig D, **Hamdan S**, **Mendlovic J** and **Gvion Y** (2023) Burnout, depression, and suicidal ideation among physicians before and during COVID-19 and the contribution of perfectionism to physicians' suicidal risk. *Frontiers in Psychiatry* **14**, 1211180.

Knowles JRP, **Gray NS**, **O'Connor C**, **Pink J**, **Simkiss NJ** and **Snowden RJ** (2022) The Role of Hope and Resilience in Protecting Against Suicidal thoughts and Behaviors During the COVID-19 Pandemic. *Archives of Suicide Research* **26**(3), 1487–1504.

Knudsen AKS, **Stene-Larsen K**, **Gustavson K**, **Hotopf M**, **Kessler RC**, **Krokstad S**, **Skogen JC**, **Øverland S** and **Reneflot A** (2021) Prevalence of mental disorders, suicidal ideation and suicides in the general population before and during the COVID-19 pandemic in Norway: A population-based repeated cross-sectional analysis. *The Lancet Regional Health - Europe* **4**, 100071.

Kohls E, **Baldofski S**, **Moeller R**, **Klemm S-L** and **Rummel-Kluge C** (2021) Mental Health, Social and Emotional Well-Being, and Perceived Burdens of University Students During COVID-19 Pandemic Lockdown in Germany. *Frontiers in Psychiatry* **12**, 643957.

Kohls E, **Guenthner L**, **Baldofski S**, **Brock T**, **Schuhr J** and **Rummel-Kluge C** (2023) Two years COVID-19 pandemic: Development of university students' mental health 2020–2022. *Frontiers in Psychiatry* **14**, 1122256.

Koné A, **Horter L**, **Thomas I**, **Byrkit R**, **Lopes-Cardozo B**, **Rao CY** and **Rose C** (2022) Symptoms of Mental Health Conditions and Suicidal Ideation Among State, Tribal, Local, and Territorial Public Health Workers — United States, March 14–25, 2022. *MMWR. Morbidity and Mortality Weekly Report* **71**(29), 925–930.

Kone A, **Horter L**, **Rose C**, **Rao CY**, **Orquiola D**, **Thomas I**, **Byrkit R**, **Bryant-Genevier J** and **Lopes-Cardozo B** (2022) The impact of traumatic experiences, coping mechanisms, and workplace benefits on the mental health of U.S. public health workers during the COVID-19 pandemic. *Annals of Epidemiology* **74**, 66–74.

Korkmaz S, **Yildiz A**, **Erdas M**, **Karakaya O** and **Goka E** (2022) Mental Health Status of Healthcare Workers during the COVID-19 Pandemic in Turkey: Results from the Vaccination Phase. *Psychiatry and Behavioral Sciences* **12**(4), 164.

Kundu A, **Fu R**, **Grace D**, **Logie C**, **Abramovich A**, **Baskerville B**, **Yager C**, **Schwartz R**, **Mitsakakis N**, **Planinac L** and **Chaiton M** (2022) Correlates of past year suicidal thoughts among sexual and gender minority young adults: A machine learning analysis. *Journal of Psychiatric Research* **152**, 269–277.

Lam MI, **Cai H**, **Chen P**, **Lok K-I**, **Chow IHI**, **Si TL**, **Su Z**, **Ng CH**, **An F-R** and **Xiang Y-T** (2024) The Inter-Relationships Between Depressive Symptoms and Suicidality Among Macau

Residents After the “Relatively Static Management” COVID-19 Strategy: A Perspective of Network Analysis. *Neuropsychiatric Disease and Treatment* **Volume 20**, 195–209.

Landi G, **Grossman-Giron A**, **Bitan DT**, **Mikulincer M**, **Grandi S** and **Tossani E** (2023) Mental Pain, Psychological Distress, and Suicidal Ideation During the COVID-19 Emergency: the Moderating Role of Tolerance for Mental Pain. *International Journal of Mental Health and Addiction* **21**(2), 1120–1131.

Lantos JD, **Yeh H-W**, **Raza F**, **Connelly M**, **Goggin K** and **Sullivant SA** (2022) Suicide Risk in Adolescents During the COVID-19 Pandemic. *Pediatrics* **149**(2), e2021053486.

Lázaro-Pérez C, **Gómez PM**, **Martínez-López JÁ** and **Gómez-Galán J** (2023) Predictive Factors of Suicidal Ideation in Spanish University Students: A Health, Preventive, Social, and Cultural Approach. *Journal of Clinical Medicine* **12**(3), 1207.

Leaune E, **Vieux M**, **Marchal M**, **Combes C**, **Crandall S**, **Haesebaert J** and **Poulet E** (2022) Self-reported mental health symptoms, quality of life and coping strategies in French health sciences students during the early stage of the COVID-19 pandemic: An online survey. *L’Encéphale* **48**(6), 607–614.

Lee D, **Hyun SY**, **Kim H**, **Kwak E**, **Lee S**, **Baik M**, **Paik J-W**, **Sim M** and **Jung SJ** (2024) Comparative analysis of mental health impairment among COVID-19 confirmed cases across the pandemic period in South Korea. *Asian Journal of Psychiatry* **101**, 104233.

Lee E and **Nam JY** (2023) Is a change in economic status associated with anxiety, depression and suicide ideation during the COVID-19 pandemic in South Korean adults? *Journal of Public Health* **45**(4), 870–877.

Leroy A, **Wathelet M**, **Fovet T**, **Habran E**, **Granon B**, **Martignène N**, **Amad A**, **Notredame C-E**, **Vaiva G** and **D’Hondt F** (2021) Mental health among medical, healthcare, and other university students during the first COVID-19 lockdown in France. *Journal of Affective Disorders Reports* **6**, 100260.

Lewis KC, **Roche MJ**, **Brown F** and **Tillman JG** (2023) Intolerance of aloneness as a prospective predictor of suicidal ideation during COVID-19. *Journal of Affective Disorders Reports* **11**, 100469.

Li D-J, **Ko N-Y**, **Chen Y-L**, **Wang P-W**, **Chang Y-P**, **Yen C-F** and **Lu W-H** (2020) COVID-19-Related Factors Associated with Sleep Disturbance and Suicidal Thoughts among the Taiwanese Public: A Facebook Survey. *International Journal of Environmental Research and Public Health* **17**(12), 4479.

Li S, **Liu S**, **Zhang P**, **Lin Y**, **Cui Y**, **Gu Y**, **Wang J**, **Liu Z** and **Zhang B** (2023) Suicidal ideation in the general population in China after the COVID-19 pandemic was initially controlled. *Journal of Affective Disorders* **323**, 834–840.

Li W, **Xiang M**, **Zhang EL**, **Liu Y**, **Ge X**, **Su Z**, **Cheung T**, **Jackson T** and **Xiang Y-T** (2024) Inter-relationships between suicidality and depressive symptoms among children and adolescents experiencing crisis: A network perspective. *Journal of Affective Disorders* **354**, 44–50.

Li Y-C, **Bai W**, **Cai H**, **Wu Y**, **Zhang Ling**, **Ding Y-H**, **Yang J-J**, **Du X**, **Zeng Z-T**, **Lu C-M**, **Feng K-X**, **Mi W-F**, **Zhang Lan**, **Liu H-Z**, **Balbuena L**, **Cheung T**, **Su Z**, **An F-R** and **Xiang Y-T** (2022) Suicidality in clinically stable bipolar disorder and schizophrenia patients during the COVID-19 pandemic. *Translational Psychiatry* **12**(1), 303.

Li Z, **Liu D**, **Liu X**, **Su H** and **Bai S** (2023) The Association of Experienced Long Working Hours and Depression, Anxiety, and Suicidal Ideation Among Chinese Medical Residents During the COVID-19 Pandemic: A Multi-Center Cross-Sectional Study. *Psychology Research and Behavior Management* **Volume 16**, 1459–1470.

Liang S, **Liu L**, **Peng X**, **Chen J**, **Huang A**, **Wang X**, **Zhao J**, **Fan F** and **Liu X** (2022) Prevalence and associated factors of suicidal ideation among college students during the COVID-19 pandemic in China: a 3-wave repeated survey. *BMC Psychiatry* **22**(1), 336.

Liang Y-J, **Deng F**, **Liang P** and **Zhong B-L** (2022) Suicidal Ideation and Mental Health Help-Seeking Behaviors Among Older Chinese Adults During the COVID-19 Pandemic. *Journal of Geriatric Psychiatry and Neurology* **35**(2), 245–251.

Lin C-Y, **Alimoradi Z**, **Ehsani N**, **Ohayon MM**, **Chen S-H**, **Griffiths MD** and **Pakpour AH** (2022) Suicidal Ideation during the COVID-19 Pandemic among A Large-Scale Iranian Sample: The Roles of Generalized Trust, Insomnia, and Fear of COVID-19. *Healthcare* **10**(1), 93.

Liu J, **Chai L**, **Zhu H** and **Han Z** (2023) COVID-19 impacts and adolescent suicide: The mediating roles of child abuse and mental health conditions. *Child Abuse & Neglect* **138**, 106076.

Liu L, **Pollock NJ**, **Contreras G**, **Tonmyr L** and **Thompson W** (2023) Pandemic-related impacts and suicidal ideation among adults in Canada: a population-based cross-sectional study. *Health Promotion and Chronic Disease Prevention in Canada* **43**(3), 105–118.

Liu L, **Contreras G**, **Pollock NJ**, **Tonmyr L** and **Thompson W** (2023) Suicidal ideation among young adults in Canada during the COVID-19 pandemic: evidence from a population-based cross-sectional study. *Health Promotion and Chronic Disease Prevention in Canada* **43**(5), 260–266.

Liu L, **Batomen B**, **Pollock NJ**, **Contreras G**, **Jackson B**, **Pan S** and **Thompson W** (2023) Suicidality and protective factors among sexual and gender minority youth and adults in Canada: a cross-sectional, population-based study. *BMC Public Health* **23**(1), 1469.

Liu Y, **Yue S**, **Hu X**, **Zhu J**, **Wu Z**, **Wang J** and **Wu Y** (2021) Associations between feelings/behaviors during COVID-19 pandemic lockdown and depression/anxiety after lockdown in a sample of Chinese children and adolescents. *Journal of Affective Disorders* **284**, 98–103.

Lizhi X, **Peng C**, **Wanhong Z**, **Shengmei X**, **Lingjiang L**, **Li Z**, **Xiaoping W** and **Weihui L** (2021) Factors Associated With Preference of Psychological Intervention and Mental Status Among Chinese Teachers During Coronavirus Disease 2019: A Large Cross-Sectional Survey. *Frontiers in Psychiatry* **12**, 704010.

Lo HKY, **Chan JKN**, **Yip EWC**, **Chui EMC**, **Fung VSC**, **Wong CSM**, **Chu RST**, **So YK**, **Chan JMT**, **Chung AKK**, **Lee KCK**, **Cheng CPW**, **Law CW**, **Chan WC** and **Chang WC** (2023)

The prevalence and correlates of suicidal ideation in Chinese psychiatric patients during the fifth wave of COVID-19 in Hong Kong. *European Neuropsychopharmacology* **77**, 4–11.

Lo HKY, **Chan JKN**, **Wong CSM**, **Wong GHS**, **Lei JHC**, **So YK**, **Fung VSC**, **Chu RST**, **Ling R**, **Chung AKK**, **Lee KCK**, **Cheng CPW**, **Chan WC** and **Chang WC** (2024) Prevalence and correlates of suicidal ideation in the general public during the fifth wave of COVID-19 pandemic in Hong Kong. *Frontiers in Psychiatry* **14**, 1252600.

López Steinmetz LC, **Fong SB** and **Godoy JC** (2021) Suicidal risk and impulsivity-related traits among young Argentinean college students during a quarantine of up to 103-day duration: Longitudinal evidence from the COVID-19 pandemic. *Suicide and Life-Threatening Behavior* **51**(6), 1175–1188.

Lozano-Verduzco I, **Vega-Cauich J**, **Mendoza-Pérez JC** and **Craig SL** (2024) Perceived Social Support and Mental Health Indicators of a Mexican LGBT Sample During the COVID-19 Pandemic. *International Journal of Mental Health and Addiction* **22**(6), 3520–3537.

Lu X, **Xie Y**, **Feng H**, **Liu Z**, **Ouyang K**, **Hou B**, **Wang M**, **Kong Z**, **Zhu Z**, **Dao W**, **Zhou Y**, **Cao J**, **Long J**, **Xu Y**, **Liu Y** and **Yin X** (2021) Psychological impact on COVID-19 patients during the outbreak in China: A case-control study. *Psychiatry Research* **305**, 114180.

Luethy D, **Krueger TM**, **Cuneo E**, **Varnes JR** and **Hernandez JA** (2023) Cross-sectional study of physical activity, dietary habits, and mental health of veterinary students after lifting of COVID-19 pandemic measures. *PLOS ONE* Mubarak N (ed.) **18**(9), e0291590.

Lyngdoh M, **Sundaram SP**, **Ningombam JD** and **Medhi GK** (2023) Suicidal ideation, attempts and its determinants among young adults in Meghalaya: a cross sectional study. *International Journal of Adolescent Medicine and Health* **35**(4), 375–380.

Ma Zijuan, **Wang D**, **Zhao J**, **Zhu Y**, **Zhang Y**, **Chen Z**, **Jiang J**, **Pan Y**, **Yang Z**, **Zhu Z**, **Liu X** and **Fan F** (2022) Longitudinal associations between multiple mental health problems and suicidal ideation among university students during the COVID-19 pandemic. *Journal of Affective Disorders* **311**, 425–431.

Ma Zikun, **Mao Y**, **Wang Y**, **Duan Z**, **Qu D**, **Li C**, **Chen R** and **Liu Z** (2022) Suicidal ideation and attempted suicide among cancer patients during the COVID-19 pandemic. *Journal of Medical Virology* **94**(12), 5827–5835.

Macalli M, **Kinouani S**, **Texier N**, **Schück S** and **Tzourio C** (2022) Contribution of perceived loneliness to suicidal thoughts among French university students during the COVID-19 pandemic. *Scientific Reports* **12**(1), 16833.

Macalli M, **Castel L**, **Jacqmin-Gadda H**, **Galesne C**, **Tournier M**, **Galéra C**, **Pereira E** and **Tzourio C** (2025) Depressive symptoms and suicidal ideation among university students before and after the COVID-19 pandemic. *Journal of Affective Disorders* **369**, 149–154.

MacDonald BV, **Wong SJ**, **Maxwell B**, **Carter C**, **Sanderson K** and **Carvalho D** (2022) Depression in the Pediatric Otolaryngology Clinic Setting. *The Laryngoscope* **132**(5), 1104–1111.

Mailloux B, **Brassard A**, **Audet A**, **Péloquin K**, **Savard C**, **Daspe M-È**, **Lafontaine M-F** and **Godbout N** (2024) Comportements suicidaires chez les hommes en demande d'aide : Rôles des traumatismes en enfance, de l'attachement et des impacts perçus de la pandémie. *Canadian Journal of Behavioural Science / Revue canadienne des sciences du comportement* **56**(4), 387–398.

Malandain L, **Fountoulakis KN**, **Syunyakov T**, **Malashonkova E**, **Smirnova D** and **Thibaut F** (2022) Psychoactive substance use, internet use and mental health changes during the COVID-19 lockdown in a French population: A study of gender effect. *Frontiers in Psychiatry* **13**, 958988.

Mallhi TH, **Ahmad N**, **Salman M**, **Tanveer N**, **Shah S**, **Butt MH**, **Alatawi AD**, **Alotaibi NH**, **Rahman HU**, **Alzarea AI**, **Alanazi AS**, **Alzahrani MS**, **Alshehri S**, **Aljabri A** and **Khan YH** (2022) Estimation of Psychological Impairment and Coping Strategies during COVID-19 Pandemic among University Students in Saudi Arabia: A Large Regional Analysis. *International Journal of Environmental Research and Public Health* **19**(21), 14282.

Mamun Mohammed A, **Al Mamun F**, **Hosen I**, **Hasan M**, **Rahman A**, **Jubayar AM**, **Maliha Z**, **Abdullah AH**, **Sarker MA**, **Kabir H**, **Jyoti AS**, **Kaggwa MM** and **Sikder MT** (2021) Suicidality in Bangladeshi Young Adults During the COVID-19 Pandemic: The Role of Behavioral Factors, COVID-19 Risk and Fear, and Mental Health Problems. *Risk Management and Healthcare Policy* **Volume 14**, 4051–4061.

Mamun Mohammed A., **Sakib N**, **Gozal D**, **Bhuiyan AI**, **Hossain S**, **Bodrud-Doza Md**, **Al Mamun F**, **Hosen I**, **Safiq MB**, **Abdullah AH**, **Sarker MdA**, **Rayhan I**, **Sikder MdT**, **Muhit M**, **Lin C-Y**, **Griffiths MD** and **Pakpour AH** (2021) The COVID-19 pandemic and serious psychological consequences in Bangladesh: A population-based nationwide study. *Journal of Affective Disorders* **279**, 462–472.

Matić T, **Pregelj P**, **Sadikov A** and **Rus Prelog P** (2022) Depression, Anxiety, Stress, and Suicidality Levels in Young Adults Increased Two Years into the COVID-19 Pandemic. *International Journal of Environmental Research and Public Health* **20**(1), 339.

Matsushima M, **Yamada H**, **Kondo N**, **Arakawa Y** and **Tabuchi T** (2023) Married women's decision to delay childbearing, and loneliness, severe psychological distress, and suicidal ideation under crisis: online survey data analysis from 2020 to 2021. *BMC Public Health* **23**(1), 1642.

McKnight-Eily LR, **Okoro CA**, **Strine TW**, **Verlenden J**, **Hollis ND**, **Njai R**, **Mitchell EW**, **Board A**, **Puddy R** and **Thomas C** (2021) Racial and Ethnic Disparities in the Prevalence of Stress and Worry, Mental Health Conditions, and Increased Substance Use Among Adults During the COVID-19 Pandemic — United States, April and May 2020. *MMWR. Morbidity and Mortality Weekly Report* **70**(5), 162–166.

McLoughlin J, **O'Grady MM** and **Hallahan B** (2022) Impact of the COVID-19 pandemic on patients with pre-existing mood disorders. *Irish Journal of Psychological Medicine* **39**(4), 363–372.

Mediavilla R, **Fernández-Jiménez E**, **Martínez-Alés G**, **Moreno-Küstner B**, **Martínez-Morata**

I, **Jaramillo F**, **Morán-Sánchez I**, **Minué S**, **Torres-Cantero A**, **Alvarado R**, **Ayuso-Mateos JL**, **Mascayano F**, **Susser E** and **Bravo-Ortiz M-F** (2021) Role of access to personal protective equipment, treatment prioritization decisions, and changes in job functions on health workers' mental health outcomes during the initial outbreak of the COVID-19 pandemic. *Journal of Affective Disorders* **295**, 405–409.

Medvedeva T, **Enikolopov S**, **Boyko O** and **Vorontsova O** (2020) THE DYNAMICS OF DEPRESSIVE SYMPTOMS AND SUICIDAL IDEATION DURING THE COVID-19 PANDEMIC IN RUSSIA. *SUICIDOLOGY* **11**(3), 3–16.

Meller FO, **Schäfer AA**, **Quadra MR**, **Demenech LM**, **Paludo SDS**, **Da Silva PA**, **Neiva-Silva L** and **Dumith SC** (2022) Fear of Covid-19 and health-related outcomes: results from two Brazilian population-based studies. *Psychiatry Research* **313**, 114596.

Menculini G, **Moretti P**, **Pandolfi LM**, **Bianchi S**, **Valentini E**, **Gatto M**, **Amantini K** and **Tortorella A** (n.d.) SUICIDALITY AND COVID-19: DATA FROM AN EMERGENCY SETTING IN ITALY. *Psychiatria Danubina* **33**.

Merkle SL, **Welton M**, **Van Zyl A**, **Chong M**, **Tanner A**, **Rose CE**, **Hertz M**, **Hill L**, **Leroy ZC**, **Sifre K** and **Thomas ES** (2023) Symptoms of Depression, Anxiety, and Post-Traumatic Stress Disorder, and Suicidal Ideation Among School Nurses in Prekindergarten through Grade 12 Schools — United States, March 2022. *The Journal of School Nursing* **39**(2), 114–124.

Moderato L, **Lazzeroni D**, **Oppo A**, **Dell'Orco F**, **Moderato P** and **Presti G** (2021) Acute Stress Response Profiles in Health Workers Facing SARS-CoV-2. *Frontiers in Psychology* **12**, 660156.

Mohd Fadhli SA, **Liew Suet Yan J**, **Ab Halim AS**, **Ab Razak A** and **Ab Rahman A** (2022) Finding the Link between Cyberbullying and Suicidal Behaviour among Adolescents in Peninsular Malaysia. *Healthcare* **10**(5), 856.

Molina AMS, **Gómez-Sierra FJ**, **Ruiz JF**, **Jiménez-Fernández S**, **Domenech PG**, **García-Jiménez J** and **Gutiérrez-Rojas L** (2023) Consequences of the COVID-19 pandemic on the mental health of medical students. *Actas Españolas de Psiquiatría* **51**(5), 202.

Molina JD, **Amigo F**, **Vilagut G**, **Mortier P**, **Muñoz-Ruiperez C**, **Rodrigo Holgado I**, **Juanes González A**, **Combarro Ripoll CE**, **Alonso J** and **Rubio G** (2024) Impact of COVID-19 first wave on the mental health of healthcare workers in a Front-Line Spanish Tertiary Hospital: lessons learned. *Scientific Reports* **14**(1), 8149.

Monteith LL, **Schneider AL**, **Holliday R**, **Barnes SM**, **Mohatt NV**, **Brenner LA** and **Hoffmire CA** (2023) A mixed methods analysis of U.S. military veterans' suicidal ideation experiences during the COVID-19 pandemic: Prevalence, correlates, and perceived impact. *Mental Health & Prevention* **31**, 200294.

Moola S, Munn Z, Tufanaru C, Aromataris E, Sears K, Sfetcu R, Currie M, Qureshi R, Mattis P, Lisy K, Mu P-F (2020) Chapter 7: Systematic reviews of etiology and risk. In: Aromataris E, Munn Z (Editors). *JBI Manual for Evidence Synthesis*. Available from https://synthesismanual.jbi.global.

Mosolova E, **Sosin D** and **Mosolov S** (2021) Stress, anxiety, depression and burnout in frontline healthcare workers during two peaks of COVID-19 pandemic in Russia. *Psychiatry Research* **306**, 114226.

Mucci M, **Lenzi F**, **D'Acunto GM**, **Gazzillo M**, **Accorinti I**, **Boldrini S**, **Distefano G**, **Falcone F**, **Fossati B**, **Giurdanella Annina R**, **Paese S**, **Salluce C**, **Troiano I**, **Fratoni C**, **Fabiani D**, **Liboni F** and **Masi G** (2022) How COVID-19 Phases Have Impacted Psychiatric Risk: A Retrospective Study in an Emergency Care Unit for Adolescents. *Children* **9**(12), 1921.

Muneeb NUA and **Hassan SMU** (2023) Psychological strain and suicidal ideation in young university students during Covid-19 outbreak: the mediating roles of rumination and depression. *Current Psychology* **42**(27), 23731–23742.

Murata S, **Rezeppa T**, **Thoma B**, **Marengo L**, **Krancevich K**, **Chiyka E**, **Hayes B**, **Goodfriend E**, **Deal M**, **Zhong Y**, **Brummit B**, **Coury T**, **Riston S**, **Brent DA** and **Melhem NM** (2021) The psychiatric sequelae of the COVID-19 pandemic in adolescents, adults, and health care workers. *Depression and Anxiety* **38**(2), 233–246.

Mushtaque I, **Rizwan M**, **Abbas M**, **Khan AA**, **Fatima SM**, **Jaffri QA**, **Mushtaq R**, **Hussain S**, **Shabbir SW**, **Naz R** and **Muneer K** (2024) Inter-Parental Conflict's Persistent Effects on Adolescent Psychological Distress, Adjustment Issues, and Suicidal Ideation During the COVID-19 Lockdown. *OMEGA - Journal of Death and Dying* **88**(3), 919–935.

Mzumara T (2024) Excessive internet use as a risk factor for suicide ideation among university students in Malawi: A cross-sectional study. *Health Science Reports* **7**(6), e2194.

N. Fountoulakis K, **N. Karakatsoulis G**, **Abraham S**, **Adorjan K**, **Ahmed HU**, **Alarcón RD**, **Arai K**, **Auwal SS**, **Bobes J**, **Bobes-Bascaran T**, **Bourgin-Duchesnay J**, **Bredicean CA**, **Bukelskis L**, **Burkadze A**, **Cabrera Abud II**, **Castilla-Puentes R**, **Cetkovich M**, **Colon-Rivera H**, **Corral R**, **Cortez-Vergara C**, **Crepin P**, **De Berardis D**, **Zamora Delgado S**, **De Lucena D**, **De Sousa A**, **Di Stefano R**, **Dodd S**, **Elek LP**, **Elissa A**, **Erdelyi-Hamza B**, **Erzin G**, **Etchevers MJ**, **Falkai P**, **Farcas A**, **Fedotov I**, **Filatova V**, **Fountoulakis NK**, **Frankova I**, **Franza F**, **Frias P**, **Galako T**, **Garay CJ**, **Garcia-Álvarez L**, **García-Portilla P**, **Gonda X**, **Gondek TM**, **Morera González D**, **Gould H**, **Grandinetti P**, **Grau A**, **Groudeva V**, **Hagin M**, **Harada T**, **Hasan TM**, **Azreen Hashim N**, **Hilbig J**, **Hossain S**, **Iakimova R**, **Ibrahim M**, **Iftene F**, **Ignatenko Y**, **Irarrazaval M**, **Ismail Z**, **Ismayilova J**, **Jacobs A**, **Jakovljević M**, **Jakšić N**, **Javed A**, **Yilmaz Kafali H**, **Karia S**, **Kazakova O**, **Khalifa D**, **Khaustova O**, **Koh S**, **Kopishinskaia S**, **Kosenko K**, **Koupidis SA**, **Kovacs I**, **Kulig B**, **Lalljee A**, **Liewig J**, **Majid A**, **Malashonkova E**, **Malik K**, **Iqbal Malik N**, **Mammadzada G**, **Mandalia B**, **Marazziti D**, **Marčinko D**, **Martinez S**, **Matiekus E**, **Mejia G**, **Memon RS**, **Meza Martínez XE**, **Mickevičiūtė D**, **Milev R**, **Mohammed M**, **Molina-López A**, **Morozov P**, **Muhammad NS**, **Mustač F**, **Naor MS**, **Nassieb A**, **Navickas A**, **Okasha T**, **Pandova M**, **Panfil A-L**, **Panteleeva L**, **Papava I**, **Patsali ME**, **Pavlichenko A**, **Pejuskovic B**, **Pinto Da Costa M**, **Popkov M**, **Popovic D**, **Raduan NJN**, **Vargas Ramírez F**, **Rancans E**, **Razali S**, **Rebok F**, **Rewekant A**, **Reyes Flores EN**, **Rivera-Encinas MT**, **Saiz PA**, **Sánchez De Carmona M**, **Saucedo Martínez D**, **Saw JA**, **Saygili G**, **Schneidereit P**, **Shah B**, **Shirasaka T**, **Silagadze K**, **Sitanggang S**, **Skugarevsky O**, **Spikina A**, **Mahalingappa SS**, **Stoyanova M**,

Szczegielniak A, **Tamasan SC**, **Tavormina G**, **Tavormina MGM**, **Theodorakis PN**, **Tohen M**, **Tsapakis E-M**, **Tukhvatullina D**, **Ullah I**, **Vaidya R**, **Vega-Dienstmaier JM**, **Vrublevska J**, **Vukovic O**, **Vysotska O**, **Widiasih N**, **Yashikhina A**, **Prezerakos PE**, **Berk M**, **Levaj S** and **Smirnova D** (2023) Results of the COVID-19 mental health international for the health professionals (COMET-HP) study: depression, suicidal tendencies and conspiracism. *Social Psychiatry and Psychiatric Epidemiology* **58**(9), 1387–1410.

Na PJ, **Tsai J**, **Hill ML**, **Nichter B**, **Norman SB**, **Southwick SM** and **Pietrzak RH** (2021) Prevalence, risk and protective factors associated with suicidal ideation during the COVID-19 pandemic in U.S. military veterans with pre-existing psychiatric conditions. *Journal of Psychiatric Research* **137**, 351–359.

Nahrin R, **Al-Mamun F**, **Kaggwa MM**, **Al Mamun Md** and **Mamun MA** (2023) Prevalence and factors associated with suicidal ideation among students taking university entrance tests: revisited and a study based on Geographic Information System data. *BJPsych Open* **9**(4), e129.

Narita Z, **Devylder J**, **Bessaha M** and **Fedina L** (2023) Associations of self-isolation, social support and coping strategies with depression and suicidal ideation in U.S. young adults during the COVID -19 pandemic. *International Journal of Mental Health Nursing* **32**(3), 929–937.

Niederkrotenthaler T, **Laido Z**, **Kirchner S**, **Braun M**, **Metzler H**, **Waldhör T**, **Strauss MJ**, **Garcia D** and **Till B** (2022) Mental health over nine months during the SARS-CoV2 pandemic: Representative cross-sectional survey in twelve waves between April and December 2020 in Austria. *Journal of Affective Disorders* **296**, 49–58.

Nigatu YT, **Elton-Marshall T** and **Hamilton HA** (2023) Changes in household debt due to COVID-19 and mental health concerns among adults in Ontario, Canada. *International Journal of Social Psychiatry* **69**(3), 774–783.

Oh J, **Kim M**, **Rhee SY**, **Rahmati M**, **Koyanagi A**, **Smith L**, **Kim MS**, **Fond G**, **Boyer L**, **Kim S**, **Shin JI** and **Yon DK** (2024) National Trends in the Prevalence of Screen Time and Its Association With Biopsychosocial Risk Factors Among Korean Adolescents, 2008–2021. *Journal of Adolescent Health* **74**(3), 504–513.

Okubo R, **Yoshioka T**, **Nakaya T**, **Hanibuchi T**, **Okano H**, **Ikezawa S**, **Tsuno K**, **Murayama H** and **Tabuchi T** (2021) Urbanization level and neighborhood deprivation, not COVID-19 case numbers by residence area, are associated with severe psychological distress and new-onset suicidal ideation during the COVID-19 pandemic. *Journal of Affective Disorders* **287**, 89–95.

Olié E, **Dubois J**, **Benramdane M**, **Guillaume S** and **Courtet P** (2022) Poor mental health is associated with loneliness and boredom during Covid-19-related restriction periods in patients with pre-existing depression. *Journal of Affective Disorders* **319**, 446–461.

Oliveira MMD, **Treichel CADS**, **Bakolis I**, **Alves PF**, **Coimbra VCC**, **Cavada GP**, **Sperb LCSDO**, **Guedes ADC**, **Antonacci MH** and **Willrich JQ** (2022) Mental health of nursing professionals during the COVID-19 pandemic: a cross-sectional study. *Revista de Saúde Pública* **56**, 1–8.

Ori APS, **Wieling M** and **Van Loo HM** (2023) Longitudinal analyses of depression, anxiety, and

suicidal ideation highlight greater prevalence in the northern Dutch population during the COVID-19 lockdowns. *Journal of Affective Disorders* **323**, 62–70.

Osiogo F, **Shalaby R**, **Adegboyega S**, **Hrabok M**, **Gusnowski A**, **Vuong W**, **Surood S**, **Greenshaw AJ** and **Agyapong VIO** (2021) COVID-19 pandemic: demographic and clinical correlates of disturbed sleep among 6,041 Canadians. *International Journal of Psychiatry in Clinical Practice* **25**(2), 164–171.

Özçelik N, **Özyurt S**, **Topaloğlu EŞ**, **Gümüş A**, **Hocaoğlu Ç** and **Şahin Ü** (2025) Long COVID: A risk factor for anxiety, depression, and suicidality? *Journal of Investigative Medicine* **73**(1), 67–74.

Padmanathan P, **Lamb D**, **Scott H**, **Stevelink S**, **Greenberg N**, **Hotopf M**, **Morriss R**, **Raine R**, **Rafferty AM**, **Madan I**, **Dorrington S**, **Wessely S** and **Moran P** (2023) Suicidal thoughts and behaviour among healthcare workers in England during the COVID-19 pandemic: A longitudinal study. *PLOS ONE* Lennox C (ed.) **18**(6), e0286207.

Palagini L, **Alfi G**, **Dazzi D**, **Gemignani A**, **Caruso V**, **Geoffroy PA**, **Miniati M** and **Straudi S** (2023) Poor sleep quality may independently predict suicidal risk in COVID-19 survivors: a 2-year longitudinal study. **20**(4), 271–278.

Papadopoulou A, **Efstathiou V**, **Yotsidi V**, **Pomini V**, **Michopoulos I**, **Markopoulou E**, **Papadopoulou M**, **Tsigkaropoulou E**, **Kalemi G**, **Tournikioti K**, **Douzenis A** and **Gournellis R** (2021) Suicidal ideation during COVID-19 lockdown in Greece: Prevalence in the community, risk and protective factors. *Psychiatry Research* **297**, 113713.

Park B, **Kim J**, **Yang J**, **Choi S** and **Oh K** (2023) Changes in mental health of Korean adolescents before and during the COVID-19 pandemic: a special report using the Korea Youth Risk Behavior Survey. *Epidemiology and Health* **45**, e2023019.

Park C, **McClure Fuller M**, **Echevarria TM**, **Nguyen K**, **Perez D**, **Masood H**, **Alsharif T** and **Worthen M** (2023) A participatory study of college students' mental health during the first year of the COVID-19 pandemic. *Frontiers in Public Health* **11**, 1116865.

Park D and **Park S** (2024) Role of Stigma in Moderating the Effects of Loneliness on Mental Health Problems Among Patients With COVID-19 in South Korea. *Psychiatry Investigation* **21**(6), 590–600.

Park J-Y and **Lee I** (2022) Factors influencing suicidal tendencies during COVID-19 pandemic in Korean multicultural adolescents: a cross-sectional study. *BMC Psychology* **10**(1), 158.

Pelissier C, **Viale M**, **Berthelot P**, **Poizat B**, **Massoubre C**, **Tiffet T** and **Fontana L** (2021) Factors Associated with Psychological Distress in French Medical Students during the COVID-19 Health Crisis: A Cross-Sectional Study. *International Journal of Environmental Research and Public Health* **18**(24), 12951.

Peng P, **Chen Q**, **Liang M**, **Liu Y**, **Chen S**, **Wang Yunfei**, **Yang Q**, **Wang X**, **Li M**, **Wang Yingying**, **Hao Y**, **He L**, **Wang Q**, **Zhang J**, **Ma Y**, **He H**, **Zhou Y**, **Li Z**, **Xu H**, **Long J**, **Qi C**, **Tang Y-Y**, **Liao Y**, **Tang J**, **Wu Q** and **Liu T** (2022) A network analysis of anxiety and depression symptoms among Chinese nurses in the late stage of the COVID-19 pandemic.

Frontiers in Public Health **10**, 996386.

Peng X, **Liang S**, **Liu L**, **Cai C**, **Chen J**, **Huang A**, **Wang X** and **Zhao J** (2023) Prevalence and associated factors of depression, anxiety and suicidality among Chinese high school E-learning students during the COVID-19 lockdown. *Current Psychology* **42**(34), 30653–30664.

Peper-Nascimento J, **Rogers ML**, **Madeira K**, **Keller GS**, **Richards JA**, **Ceretta LB**, **Quevedo J**, **Galynker I** and **Valvassori SS** (2024) Influence of changes in occupational status during the COVID-19 pandemic on suicidal narrative, suicide crisis syndrome, and suicidal ideation in Brazil. *Ciência & Saúde Coletiva* **29**(10), e01862023.

Pouradeli S, **Ahmadinia H** and **Rezaeian M** (2023) Prevalence and Risk Factors for Suicidal Ideation Following the COVID-19 Pandemic in Kerman Province: A Cross-Sectional Study. *Archives of Iranian Medicine* **26**(12), 701–708.

Pouradeli S, **Khadir E**, **Rezaeian M** and **Meimand HAE** (2024) Exploring suicidal ideation prevalence in multiple sclerosis patients during the COVID-19 pandemic: A study on the relationship between drug use and suicidal ideation. *Multiple Sclerosis and Related Disorders* **87**, 105676.

Prado ADS, **Kohls E**, **Baldofski S**, **Bianchi AS**, **Trindade LIP**, **Freitas JDL** and **Rummel-Kluge C** (2023) How are Brazilian university students coping with the COVID-19 pandemic? Results of an online survey on psychosocial well-being, perceived burdens, and attitudes toward social distancing and vaccination. *PLOS ONE* Osório FL (ed.) **18**(4), e0284190.

Prawira B, **Liem A**, **Jony Eko Y** and **Jin H** (2023) The associated factors of self-harm and suicide ideation among Chinese Indonesians during the COVID-19 pandemic. *International perspectives in psychology* **12**(2), 85–95.

Pyszczynski T, **Lockett M**, **Greenberg J** and **Solomon S** (2021) Terror Management Theory and the COVID-19 Pandemic. *Journal of Humanistic Psychology* **61**(2), 173–189.

Que J-Y, **Shi L**, **Yan W**, **Chen S-J**, **Wu P**, **Sun S-W**, **Yuan K**, **Liu Z-C**, **Zhu Z**, **Fan J-Y**, **Lu Y**, **Hu B**, **Xiao H**, **Liu Z-S**, **Li Y**, **Wang G-H**, **Wang W**, **Ran M-S**, **Shi J**, **Wing YK**, **Bao Y-P** and **Lu L** (2022) Nightmares mediate the association between traumatic event exposure and suicidal ideation in frontline medical workers exposed to COVID-19. *Journal of Affective Disorders* **304**, 12–19.

Rabasco A, **Corcoran V** and **Andover M** (2021) Alone but not lonely: The relationship between COVID-19 social factors, loneliness, depression, and suicidal ideation. *PLOS ONE* Patel SKS (ed.) **16**(12), e0261867.

Ramirez S, **Valdés J**, **Díaz F**, **Solorza F**, **Christiansen P**, **Lorca G** and **Gaete J** (2022) Mental health and associated factors among undergraduate students during Covid-19 pandemic in Chile. *European Psychiatry* **65**(S1). 2022/09/01 edn. Cambridge University Press, S338–S339.

Ramos-Martín J, **Pérez-Berlanga JM**, **Oliver J** and **Moreno-Küstner B** (2023) Non-lethal suicidal behavior in university students of Spain during COVID-19. *Frontiers in Psychiatry* **14**, 1155171.

Rappaport DI, **O'Connor M**, **Reedy C** and **Vo M** (2024) Clinical Characteristics of US Adolescents Hospitalized for Eating Disorders 2010–2022. *Hospital Pediatrics* **14**(1), 52–58.

Rathod S, **Pallikadavath S**, **Young AH**, **Graves L**, **Rahman MM**, **Brooks A**, **Soomro M**, **Rathod P** and **Phiri P** (2020) Psychological impact of COVID-19 pandemic: Protocol and results of first three weeks from an international cross-section survey - focus on health professionals. *Journal of Affective Disorders Reports* **1**, 100005.

Raviv T, **Warren CM**, **Washburn JJ**, **Kanaley MK**, **Eihentale L**, **Goldenthal HJ**, **Russo J**, **Martin CP**, **Lombard LS**, **Tully J**, **Fox K** and **Gupta R** (2021) Caregiver Perceptions of Children's Psychological Well-being During the COVID-19 Pandemic. *JAMA Network Open* **4**(4), e2111103.

Regaieg N, **Zouari L**, **Mejdoub Y**, **Omri S**, **Gassara I**, **Feki R**, **Smaoui N**, **Maalej M**, **Thabet JB**, **Charfi N** and **Maalej M** (2022) The psychological impact of the COVID-19 pandemic on secondary school teachers in Sfax, Tunisia : anxiety disorders. *European Psychiatry* **65**(S1), S336–S337.

Reinke M, **Falke C**, **Cohen K**, **Anderson D**, **Cullen KR** and **Nielson JL** (2023) Increased suicidal ideation and suicide attempts in COVID-19 patients in the United States: Statistics from a large national insurance billing database. *Psychiatry Research* **323**, 115164.

Ren Y, **Qian W**, **Li Z**, **Liu Z**, **Zhou Y**, **Wang R**, **Qi L**, **Yang J**, **Song X**, **Zeng L** and **Zhang X** (2020) Public mental health under the long-term influence of COVID-19 in China: Geographical and temporal distribution. *Journal of Affective Disorders* **277**, 893–900.

Reyniers T, **Buffel V**, **Thunnissen E**, **Vuylsteke B**, **Siegel M**, **Nöstlinger C** and **Wouters E** (2022) Increased Anxiety and Depression Among Belgian Sexual Minority Groups During the First COVID-19 Lockdown—Results From an Online Survey. *Frontiers in Public Health* **10**, 797093.

Rhee TG, **Bommersbach TJ**, **Rosenheck RA**, **Nierenberg AA** and **McIntyre RS** (2024) National trends and correlates of treatment resistance in major depressive episode and associated suicidal ideation and behaviors among adults in the United States. *Journal of Affective Disorders* **358**, 342–349.

Richardson C, **Goodyear T**, **Slemon A**, **Gadermann A**, **Thomson KC**, **Daly Z**, **McAuliffe C**, **Pumarino J** and **Jenkins EK** (2022) Emotional response patterns, mental health, and structural vulnerability during the COVID-19 pandemic in Canada: a latent class analysis. *BMC Public Health* **22**(1), 2344.

Rifai A, **Wu W-C**, **Tang Y-W**, **Lu M-Y**, **Chiu P-J**, **Strong C**, **Lin C-Y**, **Chen P-L**, **Ko W-C** and **Ko N-Y** (2023) Psychological Distress and Physical Adverse Events of COVID-19 Vaccination among Healthcare Workers in Taiwan. *Vaccines* **11**(1), 129.

Ritish D, **Dinakaran D**, **Chander R**, **Murugesan M**, **Ibrahim FA**, **Parthasarathy R**, **Pandey PK**, **Sharma MK**, **Pandian D**, **Manjunatha N**, **Reddi SK**, **Moirangthem S**, **Kumar CN**, **Suresh BM** and **Gangadhar BN** (2020) Mental health concerns in quarantined international air passengers during COVID-19 pandemic – An experiential account. *Asian Journal of*

Psychiatry **53**, 102364.

Rizzi D, **Asperges E**, **Rovati A**, **Bigoni F**, **Pistillo E**, **Corsico A**, **Mojoli F**, **Perlini S** and **Bruno R** (2022) Psychological Support in a COVID-19 Hospital: A Community Case Study. *Frontiers in Psychology* **12**, 820074.

Rodríguez-De Avila UE, **Rodrigues-De França F** and **Jesus Simões MDF** (2021) Psychological Impact and Sleep Quality in the COVID-19 Pandemic in Brazil, Colombia and Portugal. *Duazary* **18**(2), 120–130.

Rogers ML, **Richards JA**, **Cao E**, **Krumerman M**, **Barzilay S**, **Blum Y**, **Chistopolskaya K**, **Çinka E**, **Dudeck M**, **Husain MI**, **Yilmaz FK**, **Kravtsova NA**, **Kuśmirek O**, **Menon V**, **Peper-Nascimento J**, **Pilecka B**, **Titze L**, **Valvassori SS**, **You S** and **Galynker I** (2023) Associations between long-term and near-term stressful life events, suicide crisis syndrome, and suicidal ideation. *International Journal of Stress Management* **30**(2), 209–221.

Rogers ML, **Cao E**, **Richards JA**, **Mitelman A**, **Barzilay S**, **Blum Y**, **Chistopolskaya K**, **Çinka E**, **Dudeck M**, **Husain MI**, **Kantas Yilmaz F**, **Kuśmirek O**, **Luiz JM**, **Menon V**, **Nikolaev EL**, **Pilecka B**, **Titze L**, **Valvassori SS**, **You S** and **Galynker I** (2024) Changes in Daily Behaviors and Cognitions During the COVID-19 Pandemic: Associations With Suicide Crisis Syndrome and Suicidal Ideation. *Clinical Psychological Science* **12**(1), 98–114.

Rolland F, **Hadouiri N**, **Haas-Jordache A**, **Gouy E**, **Mathieu L**, **Goulard A**, **Morvan Y** and **Frajerman A** (2022) Mental health and working conditions among French medical students: A nationwide study. *Journal of Affective Disorders* **306**, 124–130.

Roy N, **Amin MdB**, **Mamun MA**, **Hossain E**, **Aktarujjaman Md** and **Sarker B** (2023) Suicidal ideation among people with disabilities during the COVID-19 pandemic in Bangladesh: prevalence and associated factors. *BJPsych Open* **9**(1), e3.

Rozanova J, **Rich KM**, **Altice FL**, **Shenoi SV**, **Zaviryukha I**, **Kiriazova T**, **Mamedova E**, **Shipunov O**, **Yariy V**, **Deac A** and **Zeziulin O** (2022) The Initial Response to COVID-19 Disruptions for Older People with HIV in Ukraine. *Geriatrics* **7**(6), 138.

RStudio Team (2024) RStudio: integrated development environment for R (Version 2024.12.0) [Computer software]. *RStudio, PBC*. Available at: https://www.rstudio.com/ (Accessed: 8 August 2025).

Rudenstine S, **Schulder T**, **Bhatt KJ**, **McNeal K**, **Ettman CK** and **Galea S** (2022) Suicidal ideation during COVID-19: The contribution of unique and cumulative stressors. *Psychiatry Research* **310**, 114475.

Rus Prelog P, **Matić T**, **Pregelj P** and **Sadikov A** (2023) A pilot predictive model based on COVID-19 data to assess suicidal ideation indirectly. *Journal of Psychiatric Research* **163**, 318–324.

Rutkowska A, **Cieślik B**, **Tomaszczyk A** and **Szczepańska-Gieracha J** (2022) Mental Health Conditions Among E-Learning Students During the COVID-19 Pandemic. *Frontiers in Public Health* **10**, 871934.

Saeed F, **Ghalehnovi E**, **Saeidi M**, **Ali Beigi N**, **Vahedi M**, **Shalbafan M**, **Kamalzadeh L**, **Nazeri**

Astaneh A, **Jalali Nadoushan AH** and **Shoib S** (2024) Factors associated with suicidal ideation among medical residents in Tehran during the COVID-19 pandemic: A multicentric cross-sectional survey. *PLOS ONE* García-Iglesias JJ (ed.) **19**(3), e0300394.

Sahimi HMS, **Mohd Daud TI**, **Chan LF**, **Shah SA**, **Rahman FHA** and **Nik Jaafar NR** (2021) Depression and Suicidal Ideation in a Sample of Malaysian Healthcare Workers: A Preliminary Study During the COVID-19 Pandemic. *Frontiers in Psychiatry* **12**, 658174.

Sanchez Merino E, **Martínez Vispo C** and **González Sanguino C** (2024) A cross-sectional study of suicidal ideation and behaviour, depression, anxiety and stress in a Spanish sample. Mental health in young people after the Covid-19 pandemic. *Mortality* **29**(4), 645–657.

Sasaki N, **Kuroda R**, **Tsuno K**, **Imamura K** and **Kawakami N** (2021) Increased suicidal ideation in the COVID-19 pandemic: an employee cohort in Japan. *BJPsych Open* **7**(6), e199.

Sato H, **Maeda M**, **Takebayashi Y**, **Setou N**, **Shimada J** and **Kanari Y** (2004) Impact of Unexpected In-House Major COVID-19 Outbreaks on Depressive Symptoms among Healthcare Workers: A Retrospective Multi-Institutional Study. *International Journal of Environmental Research and Public Health* **20**, 4718.

Schad A, **Layton RL**, **Ragland D** and **Cook JG** (2022) Mental health in medical and biomedical doctoral students during the 2020 COVID-19 pandemic and racial protests. *eLife* **11**, e69960.

Schmits E, **Dekeyser S**, **Klein O**, **Luminet O**, **Yzerbyt V** and **Glowacz F** (2021) Psychological Distress among Students in Higher Education: One Year after the Beginning of the COVID-19 Pandemic. *International Journal of Environmental Research and Public Health* **18**(14), 7445.

Scoresby K, **Jurney C**, **Fackler A**, **Tran CV**, **Nugent W** and **Strand E** (2023) Relationships between diversity demographics, psychological distress, and suicidal thinking in the veterinary profession: a nationwide cross-sectional study during COVID-19. *Frontiers in Veterinary Science* **10**, 1130826.

Seedat S, **Sengwayo M**, **Gani S**, **Mashego L**, **Ochayon J**, **Shepard A**, **Vergie C**, **Masango M**, **Makuapane LP**, **Wagner F** and **Wagner RG** (2025) Prevalence and determinants of suicidal ideation among South African Health Sciences students at the onset of the COVID-19 pandemic. *Frontiers in Psychiatry* **16**, 1492620.

Seidler ZE, **Wilson MJ**, **Oliffe JL**, **Fisher K**, **O'Connor R**, **Pirkis J** and **Rice SM** (2023) Suicidal ideation in men during COVID-19: an examination of protective factors. *BMC Psychiatry* **23**(1), 46.

Selak Š, **Crnkovič N**, **Šorgo A**, **Gabrovec B**, **Cesar K** and **Žmavc M** (2024) Resilience and social support as protective factors against suicidal ideation among tertiary students during COVID-19: a cross-sectional study. *BMC Public Health* **24**(1), 1942.

Sewall CJR, **Goldstein TR** and **Rosen D** (2021) Objectively measured digital technology use during the COVID-19 pandemic: Impact on depression, anxiety, and suicidal ideation among young adults. *Journal of Affective Disorders* **288**, 145–147.

Sharif S, **Amin F**, **Hafiz M**, **Benzel E**, **Peev N**, **Dahlan RH**, **Enchev Y**, **Pereira P** and **Vaishya S**

(2020) COVID 19–Depression and Neurosurgeons. *World Neurosurgery* **140**, e401–e410.

Shaygan M, **Salari S**, **Jaberi A** and **Shaygan S** (2024) Gender Differences in Suicidal Ideation during the COVID-19 Pandemic among Iranian Adolescents and its Contributing Factors. *Child & Family Behavior Therapy* **46**(3), 255–271.

She R, **Wong K**, **Lin J**, **Zhang Y**, **Leung K** and **Yang X** (2022) Profiles of Stress and Coping Associated With Mental, Behavioral, and Internet Use Problems Among Adolescents During the COVID-19 Pandemic: A Stratified Random Sampling and Cluster Analysis. *Frontiers in Public Health* **10**, 826911.

Shewangzaw Engda A, **Dargie Wubetu A**, **Kasahun Amogne F** and **Moltot Kitaw T** (2022) Intimate partner violence and COVID-19 among reproductive age women: A community-based cross-sectional survey, Ethiopia. *Women's Health* **18**, 17455065211068980.

Shi L, **Que J-Y**, **Lu Z-A**, **Gong Y-M**, **Liu L**, **Wang Y-H**, **Ran M-S**, **Ravindran N**, **Ravindran AV**, **Fazel S**, **Bao Y-P**, **Shi J** and **Lu L** (2021) Prevalence and correlates of suicidal ideation among the general population in China during the COVID-19 pandemic. *European Psychiatry* **64**(1), e18.

Shiraly R, **Mahdaviazad H**, **Zohrabi R** and **Griffiths MD** (2022) Suicidal ideation and its related factors among older adults: a population-based study in Southwestern Iran. *BMC Geriatrics* **22**(1), 371.

Shongwe MC and **Huang S-L** (2021) Suicidal Ideation and Predictors of Psychological Distress during the COVID-19 Pandemic in Eswatini: A Population-Based Household Telephone Survey. *International Journal of Environmental Research and Public Health* **18**(13), 6700.

Sivertsen B, **Knapstad M**, **Petrie K**, **O'Connor R**, **Lønning KJ** and **Hysing M** (2022) Changes in mental health problems and suicidal behaviour in students and their associations with COVID-19-related restrictions in Norway: a national repeated cross-sectional analysis. *BMJ Open* **12**(2), e057492.

Sljivo A, **Smailbegovic F**, **Mulac A**, **Dadic I**, **Kubat A** and **Sirucic I** (2022) Mental Health and Substance Abuse Among the Bosnia and Herzegovina Student Population During the COVID-19 Outbreak. *Materia Socio Medica* **34**(1), 8.

Solis M, **Valverde-Barea M**, **Gutiérrez-Rojas L**, **Romera I**, **Cruz-Bailén S** and **Jiménez-Fernández S** (2023) Suicidal Risk and Depression in Pregnant Women in Times of Pandemic. *Maternal and Child Health Journal* **27**(9), 1540–1547.

Song J and **Kim MG** (2025) Factors influencing suicidal ideations among late adolescents in the era of the COVID-19 pandemic: a cross-sectional study from the Korea community health survey. *BMC Public Health* **25**(1), 1028.

Sørensen TT, **Okholm GT**, **Vendsborg P**, **Nordentoft M**, **Correll CU**, **Solmi M**, **Thompson T**, **Estradé A** and **Thygesen LC** (2025) Suicidal ideation across three waves of the COVID-19 pandemic in Denmark – identifying vulnerable subgroups using COH-FIT data. *Journal of Affective Disorders* **389**, 119656.

Spiller TR, **Na PJ**, **Merians AN**, **Duek O**, **Ben-Zion Z**, **Tsai J**, **Von Känel R**, **Harpaz-Rotem I** and **Pietrzak RH** (2023) Changes in mental health among U.S. military veterans during the COVID-19 pandemic: A network analysis. *Journal of Psychiatric Research* **165**, 352–359.

Statistics Canada (2022) Prevalence of suicidal ideation among adults in Canada: Results of the second Survey on COVID-19 and mental health. Government of Canada. Epub ahead of print 2022. DOI: 10.25318/82-003-X202200500002-ENG.

Stickley A, **Shirama A**, **Inagawa T**, **Ruchkin V**, **Koposov R**, **Isaksson J**, **Inoue Y** and **Sumiyoshi T** (2022) Attention–deficit/hyperactivity disorder symptoms, perceived stress, and suicidal ideation during the COVID-19 pandemic. *Frontiers in Psychiatry* **13**, 1008290.

Stickley A, **Shirama A** and **Sumiyoshi T** (2023) Psychotic experiences, perceived stress, and suicidal ideation among the general population during the COVID-19 pandemic: Findings from Japan. *Schizophrenia Research* **260**, 49–55.

Stickley A, **Shirama A**, **Kondo N**, **Kino S** and **Sumiyoshi T** (2025) Ikigai and suicidal ideation in Japan during the COVID-19 pandemic. *Journal of Psychiatric Research* **186**, 289–296.

Stonehouse J, **Grobler G**, **Bhoora U** and **Janse Van Rensburg MNS** (2023) Mental health symptoms among homeless shelter residents during COVID-19 lockdown in Tshwane, South Africa. *African Journal of Primary Health Care & Family Medicine* **15**(1).

Sun H-L, **Chen P**, **Bai W**, **Zhang L**, **Feng Y**, **Su Z**, **Cheung T**, **Ungvari GS**, **Cui X-L**, **Ng CH**, **An F-R** and **Xiang Y-T** (2024) Prevalence and network structure of depression, insomnia and suicidality among mental health professionals who recovered from COVID-19: a national survey in China. *Translational Psychiatry* **14**(1), 227.

Sun M, **Wang D**, **Jing L** and **Zhou L** (2023) The predictive role of psychotic-like experiences in suicidal ideation among technical secondary school and college students during the COVID-19 pandemic. *BMC Psychiatry* **23**(1), 521.

Sun S, **Goldberg SB**, **Lin D**, **Qiao S** and **Operario D** (2021) Psychiatric symptoms, risk, and protective factors among university students in quarantine during the COVID-19 pandemic in China. *Globalization and Health* **17**(1), 15.

Supasitthumrong T, **Maes M**, **Tunvirachaisakul C**, **Rungnirundorn T**, **Zhou B**, **Li J**, **Wainipitapong S**, **Ratanajaruraks A**, **Nimnuan C**, **Kanchanatawan B**, **Thompson T**, **Solmi M** and **Correll C** (2024) Predictors of increased affective symptoms and suicidal ideation during the COVID-19 pandemic: results from a large-scale study of 14 271 Thai adults. *BMJ Mental Health* **27**(1), e300982.

Taniguchi Y, **Miyawaki A**, **Tsugawa Y**, **Murayama H**, **Tamiya N** and **Tabuchi T** (2022) Family caregiving and changes in mental health status in Japan during the COVID-19 pandemic. *Archives of Gerontology and Geriatrics* **98**, 104531.

Tantirattanakulchai P, **Hounnaklang N**, **Pongsachareonnont PF**, **Khambhiphant B**, **Win N** and **Tepjan S** (2023) Prevalence and Factors Associated with Suicidal Ideation Among Older People with Visual Impairments Attending an Eye Center During the COVID-19 Pandemic: A Hospital-Based Cross-Sectional Study. *Clinical Ophthalmology* **Volume 17**, 917–930.

Tasnim R, **Islam MdS**, **Sujan MdSH**, **Sikder MdT** and **Potenza MN** (2020) Suicidal ideation among Bangladeshi university students early during the COVID-19 pandemic: Prevalence estimates and correlates. *Children and Youth Services Review* **119**, 105703.

Teksin G (2020) Stigma-related factors and their effects on health-care workers during COVID-19 pandemics in Turkey: a multicenter study. *SiSli Etfal Hastanesi Tip Bulteni / The Medical Bulletin of Sisli Hospital*. Epub ahead of print 2020. DOI: 10.14744/SEMB.2020.02800.

Thomson KC, **Jenkins E**, **Gill R**, **Richardson CG**, **Gagné Petteni M**, **McAuliffe C** and **Gadermann AM** (2021) Impacts of the COVID-19 Pandemic on Family Mental Health in Canada: Findings from a Multi-Round Cross-Sectional Study. *International Journal of Environmental Research and Public Health* **18**(22), 12080.

Tintori A, **Pompili M**, **Ciancimino G**, **Corsetti G** and **Cerbara L** (2023) The developmental process of suicidal ideation among adolescents: social and psychological impact from a nation-wide survey. *Scientific Reports* **13**(1), 20984.

Trettel ACPT, **Muraro AP**, **Oliveira ECD**, **Nascimento VFD**, **Andrade ACDS**, **Santos ESD**, **Espinosa MM** and **Pillon SC** (2022) Factors associated with suicidal ideation during the COVID-19 pandemic in a population in the Brazilian Legal Amazon. *Ciência & Saúde Coletiva* **27**(8), 3157–3170.

Troya MI, **Joyce M**, **Khashan A**, **Buckley C**, **Chakraborti K**, **Hoevel P**, **Humphries R**, **Kearney PM**, **Kiely E**, **Murphy M**, **Perry I** and **Ella Arensman EA** (2022) Mental health following an initial period of COVID-19 restrictions findings from a cross-sectional survey in the Republic of Ireland. *HRB open research* **4**, 130.

Tsai J, **Elbogen EB**, **Huang M**, **North CS** and **Pietrzak RH** (2021) Psychological distress and alcohol use disorder during the COVID-19 era among middle- and low-income U.S. adults. *Journal of Affective Disorders* **288**, 41–49.

Tsiouris A, **Werner AM**, **Tibubos AN**, **Mülder LM**, **Reichel JL**, **Heller S**, **Schäfer M**, **Schwab L**, **Rigotti T**, **Stark B**, **Dietz P** and **Beutel ME** (2023) Mental health state and its determinants in German university students across the COVID-19 pandemic: findings from three repeated cross-sectional surveys between 2019 and 2021. *Frontiers in Public Health* **11**, 1163541.

Tsuno K and **Tabuchi T** (2021) Risk factors for workplace bullying, severe psychological distress, and suicidal ideation during the COVID-19 pandemic: a nationwide internet survey for the general working population in Japan. Occupational and Environmental Health. Available at: http://medrxiv.org/lookup/doi/10.1101/2021.11.18.21266501 (accessed 15 August 2025).

Turner BJ, **Robillard CL**, **Ames ME** and **Craig SG** (2022) Prevalence and Correlates of Suicidal Ideation and Deliberate Self-harm in Canadian Adolescents During the Coronavirus Disease 2019 Pandemic. *The Canadian Journal of Psychiatry* **67**(5), 403–406.

Urbańska J and **Słysz A** (2023) The Role Of Early Maladaptive Schemas In The Change In General Mental Health And Well-being During The Covid-19 Coronavirus Pandemic. *Journal of Evidence-Based Psychotherapies* **22**(1), 153–165.

Valladares-Garrido D, **Zila-Velasque JP**, **Santander-Hernández FM**, **Guevara-Morales MA**,

Morocho-Alburqueque N, **Failoc-Rojas VE**, **Pereira-Victorio CJ**, **Vera-Ponce VJ**, **León-Figueroa DA** and **Valladares-Garrido MJ** (2024) Association between love breakup and suicidal ideation in Peruvian medical students: a cross-sectional study during the COVID-19 pandemic. *Frontiers in Psychiatry* **14**, 1287036.

Van Der Feltz-Cornelis C, **Sweetman J**, **Merecz-Kot D**, **De Miquel C**, **Turk F** and **Olaya B** (2025) Work stress and its association with suicidal ideation, health and presenteeism during the COVID-19 pandemic: cross-sectional study in the UK health and university workforce. *BJPsych Open* **11**(4), e155.

Vidal C, **Reinert M**, **Nguyen T** and **Jun H-J** (2024) Chronic stress and lack of social support: Role in adolescent depression and suicide-related behaviors in the context of the COVID-19 pandemic. *Journal of Affective Disorders* **365**, 437–442.

Villanueva-Blasco VJ, **Villanueva-Silvestre V**, **Vázquez-Martínez A**, **De Vicente LP** and **Pérez-Gálvez B** (2024) Depression, Suicidal Ideation, and Consumption of Psychotropic Drugs During Lockdown by COVID-19 According to Gender and Age. *International Journal of Mental Health and Addiction* **22**(4), 1859–1875.

Villanueva-Silvestre V, **Vázquez-Martínez A**, **Isorna-Folgar M** and **Villanueva-Blasco V** (2022) Problematic Internet Use, Depressive Symptomatology and Suicidal Ideation in University Students During COVID-19 Confinement. *Psicothema* **4**(34), 518–527.

Villarreal Sotelo K, **Peña Cárdenas F**, **Zamorano González B**, **Vargas Orozco CM**, **Hernández Rodríguez I** and **Landero Pérez C** (2023) Prevalence of suicidal behavior in a northeastern Mexican border population during the COVID-19 pandemic. *Frontiers in Psychology* **13**, 984374.

Wang D, **Ross B**, **Zhou X**, **Meng D**, **Zhu Z**, **Zhao J**, **Fan F** and **Liu X** (2021) Sleep disturbance predicts suicidal ideation during COVID-19 pandemic: A two-wave longitudinal survey. *Journal of Psychiatric Research* **143**, 350–356.

Wang X, **Hegde S**, **Son C**, **Keller B**, **Smith A** and **Sasangohar F** (2020) Investigating Mental Health of US College Students During the COVID-19 Pandemic: Cross-Sectional Survey Study. *Journal of Medical Internet Research* **22**(9), e22817.

Wathelet M, **Duhem S**, **Vaiva G**, **Baubet T**, **Habran E**, **Veerapa E**, **Debien C**, **Molenda S**, **Horn M**, **Grandgenèvre P**, **Notredame C-E** and **D'Hondt F** (2020) Factors Associated With Mental Health Disorders Among University Students in France Confined During the COVID-19 Pandemic. *JAMA Network Open* **3**(10), e2025591.

Wathelet M, **Vincent C**, **Fovet T**, **Notredame C-E**, **Habran E**, **Martignène N**, **Baubet T**, **Vaiva G** and **D'Hondt F** (2022) Evolution in French University Students' Mental Health One Month After the First COVID-19 Related Quarantine: Results From the COSAMe Survey. *Frontiers in Psychiatry* **13**, 868369.

Wathelet M, **Horn M**, **Creupelandt C**, **Fovet T**, **Baubet T**, **Habran E**, **Martignène N**, **Vaiva G** and **D'Hondt F** (2022) Mental Health Symptoms of University Students 15 Months After the Onset of the COVID-19 Pandemic in France. *JAMA Network Open* **5**(12), e2249342.

Wei Y, **Tang J**, **Zhao J**, **Liang J**, **Li Z** and **Bai S** (2023) Association of loneliness and social isolation with mental disorders among medical residents during the COVID-19 pandemic: A multi-center cross-sectional study. *Psychiatry Research* **327**, 115233.

Wells GA, Shea B, O'Connell D, Peterson J, Welch V, Losos M, and **Tugwell P** (2014). Newcastle-Ottawa quality assessment scale cohort studies. *University of Ottawa*, **B-10**.

Wen L, **Zhang L**, **Zhu L**, **Song J**, **Wang A**, **Tao Y**, **Li H**, **Feng Y**, **Jin Y**, **Su H** and **Chang W** (2024) Depression and suicidal ideation among Chinese college students during the COVID-19 pandemic: the mediating roles of chronotype and sleep quality. *BMC Psychiatry* **24**(1), 583.

Wickham H (2016) ggplot2: elegant graphics for data analysis (2nd ed.). *Springer*. doi:10. 1007/978-3-319-24277-4

Wijesinghe C, **Chandradasa M**, **Ranwella P**, **Samaranayake A**, **Wickrama P**, **Gamage N**, **Siriwardane G**, **Goonathilake N**, **Perera S**, **Dahanayake D**, **Mendis J** and **Ranasinghe K** (2023) Survey on the psychosocial impact of COVID-19 on the Sri Lankan mental healthcare system and the needs of frontline healthcare workers in the post-covid era. *Ceylon Medical Journal* **68**(SI1), 21–26.

Wong SMY, **Ip CH**, **Hui CLM**, **Suen YN**, **Wong CSM**, **Chang WC**, **Chan SKW**, **Lee EHM**, **Lui SSY**, **Chan KT**, **Wong MTH** and **Chen EYH** (2023) Prevalence and correlates of suicidal behaviours in a representative epidemiological youth sample in Hong Kong: the significance of suicide-related rumination, family functioning, and ongoing population-level stressors. *Psychological Medicine* **53**(10), 4603–4613.

Woolverton GA, **Rastogi R**, **Brieger KK**, **Wong SHM**, **Keum BT**, **Hahm HC** and **Liu CH** (2024) Barriers and risk factors associated with non-treatment-seeking for suicidality onset during the COVID-19 pandemic among young adults. *Psychiatry Research* **340**, 116095.

Workneh F, **Worku A**, **Assefa N** and **Berhane Y** (2023) Change in depression during the COVID-19 pandemic among healthcare providers in Addis Ababa, Ethiopia. *PeerJ* **11**, e15053.

Wu O, **Lu X**, **Yeo KJ**, **Xiao Y** and **Yip P** (2022) Assessing Prevalence and Unique Risk Factors of Suicidal Ideation among First-Year University Students in China Using a Unique Multidimensional University Personality Inventor. *International Journal of Environmental Research and Public Health* **19**(17), 10786.

Wu P, **Wang S**, **Zhao X**, **Fang J**, **Tao F**, **Su P**, **Wan Y** and **Sun Y** (2023) Immediate and longer-term changes in mental health of children with parent–child separation experiences during the COVID-19 pandemic. *Child and Adolescent Psychiatry and Mental Health* **17**(1), 113.

Xiao Q-Q, **Huang X-H**, **Yang J**, **Mu Y-F**, **Wang C**, **Deng Z-Y**, **Cai J**, **Deng A-P**, **Tang W-J**, **Chen X-C**, **Shi W**, **Jiang Y**, **Xu J-J**, **Yin L**, **Huang Y**, **Zhang W** and **Ran M-S** (2024) Suicidal ideation and suicide attempts among students aged 12 to 24 after the lifting of COVID-19 restrictions in China: prevalence and associated factors. *Frontiers in Psychiatry* **15**, 1383992.

Xiao Y, **Junus A**, **Li T** and **Yip P** (2023) Temporal and spatial trends in suicide-related visits before and during the COVID-19 pandemic in the US, 2018–2021. *Journal of Affective Disorders* **324**, 24–35.

Xin M, **Luo S**, **She R**, **Yu Y**, **Li Lijuan**, **Wang S**, **Ma L**, **Tao F**, **Zhang J**, **Zhao J**, **Li Liping**, **Hu D**, **Zhang G**, **Gu J**, **Lin D**, **Wang H**, **Cai Y**, **Wang Z**, **You H**, **Hu G** and **Lau JT** (2020) Negative cognitive and psychological correlates of mandatory quarantine during the initial COVID-19 outbreak in China. *American Psychologist* **75**(5), 607–617.

Xu Y, **Su S**, **Jiang Z**, **Guo S**, **Lu Q**, **Liu L**, **Zhao Y**, **Wu P**, **Que J**, **Shi L**, **Deng J**, **Meng S**, **Yan W**, **Sun Y**, **Yuan K**, **Lin X**, **Sun S**, **Ravindran AV**, **Chen S**, **Wing YK**, **Tang X**, **Ran M**, **Lu Y**, **Shi J**, **Huang G**, **Bao Y** and **Lu L** (2021) Prevalence and Risk Factors of Mental Health Symptoms and Suicidal Behavior Among University Students in Wuhan, China During the COVID-19 Pandemic. *Frontiers in Psychiatry* **12**, 695017.

Yamashita T, **Quy PN**, **Yamada C**, **Nogami E**, **Seto-Suh E**, **Iwamoto S** and **Kato K** (2024) A cross-sectional survey of material deprivation and suicide-related ideation among Vietnamese technical interns in Japan. *Frontiers in Psychology* **14**, 1241837.

Yamazaki J, **Kizuki M** and **Fujiwara T** (2022) Association between Frequency of Conversations and Suicidal Ideation among Medical Students during COVID-19 Pandemic in Japan. *International Journal of Environmental Research and Public Health* **19**(11), 6385.

Yang X, **Song B**, **Wu A**, **Mo PKH**, **Di J**, **Wang Q**, **Lau JTF** and **Wang L** (2021) Social, Cognitive, and eHealth Mechanisms of COVID-19–Related Lockdown and Mandatory Quarantine That Potentially Affect the Mental Health of Pregnant Women in China: Cross-Sectional Survey Study. *Journal of Medical Internet Research* **23**(1), e24495.

Yang X, **Yip BHK**, **Mak ADP**, **Zhang D**, **Lee EKP** and **Wong SYS** (2021) The Differential Effects of Social Media on Depressive Symptoms and Suicidal Ideation Among the Younger and Older Adult Population in Hong Kong During the COVID-19 Pandemic: Population-Based Cross-sectional Survey Study. *JMIR Public Health and Surveillance* **7**(5), e24623.

Yang Y (2025) Suicidal ideation among US adolescents during the COVID-19 pandemic: Exploring individual, interpersonal, and community-level resilience factors. *Public Health* **242**, 199–205.

Yang Y, **Zhang EL**, **Liu Y**, **Ge X**, **Su Z**, **Cheung T**, **Ng CH**, **Xiang M** and **Xiang Y-T** (2023) Network analysis of suicidality and internet addiction symptoms among Chinese primary and secondary school students. *Journal of Affective Disorders* **339**, 145–152.

Yang Z, **Rando A** and **Mason K** (2025) A Study on Pastors and Suicide and COVID-19: How to Care for Suicidal Pastors. *Pastoral Psychology* **74**(3), 419–434.

Yao Y, **Qiao Z**, **Dong F** and **Ni J** (2023) Role of rumination and hope on negative life events and suicidal ideation under the background of normalization of pandemic prevention and control: A moderated mediation model. *Frontiers in Public Health* **10**, 898580.

Yao Z-Y, **Xu X-M**, **Li S-J**, **Zhou X-Y**, **Li S-Y**, **Li R**, **Kou C-G**, **Yao B**, **Shen Z-Z**, **Ma Y-B**, **Wang X-T**, **Liu B-P**, **Cheng S-L**, **Gao J-G**, **Wong JP-H**, **Fung KP-L** and **Jia C-X** (2024) The prevalence of 12-month suicidal ideation and associated factors among university students in China: Findings from a three-wave cross-sectional study from 2021 to 2023. *Journal of Affective Disorders* **367**, 668–677.

Yau J-TJ and **Nager AL** (2021) Adolescent and young adult stress and coping during COVID-19:

the utility of a pediatric emergency department screener. *International Journal of Emergency Medicine* **14**(1), 41.

Young KP, **Kolcz DL**, **O'Sullivan DM**, **Ferrand J**, **Fried J** and **Robinson K** (2021) Health Care Workers' Mental Health and Quality of Life During COVID-19: Results From a Mid-Pandemic, National Survey. *Psychiatric Services* **72**(2), 122–128.

Yu Y, **Wu T**, **Wang S**, **Liu W** and **Zhao X** (2022) Suicide Risk and Association With the Different Trauma During the COVID-19 Pandemic Period: A Cross-Sectional Study on Adolescent With Different Learning Stage in Chongqing, China. *Frontiers in Public Health* **10**, 858157.

Zhai Y and **Du X** (2022) Trends and prevalence of suicide 2017–2021 and its association with COVID-19: Interrupted time series analysis of a national sample of college students in the United States. *Psychiatry Research* **316**, 114796.

Zhang L, **Cai H**, **Bai W**, **Zou S-Y**, **Feng K-X**, **Li Y-C**, **Liu H-Z**, **Du X**, **Zeng Z-T**, **Lu C-M**, **Zhang Lan**, **Mi W-F**, **Ding Y-H**, **Yang J-J**, **Jackson T**, **Cheung T**, **Su Z**, **An F-R** and **Xiang Y-T** (2022) Prevalence of suicidality in clinically stable patients with major depressive disorder during the COVID-19 pandemic. *Journal of Affective Disorders* **307**, 142–148.

Zhang X, **Liu X**, **Mi Y**, **Wang W** and **Xu H** (2022) Resilience and Depressive Symptoms Mediated Pathways from Social Support to Suicidal Ideation Among Undergraduates During the COVID-19 Campus Lockdown in China. *Psychology Research and Behavior Management* **Volume 15**, 2291–2301.

Zhang Yuwei, **Jia Y**, **MuLaTiHaJi M**, **Mi Y**, **Mei Y**, **Sun T**, **Shi H**, **Zhang Yifei**, **Zhang Yikun**, **Zou R**, **Niu L** and **Dong S** (2024) A cross-sectional mental-health survey of Chinese postgraduate students majoring in stomatology post COVID-19 restrictions. *Frontiers in Public Health* **12**, 1376540.

Zheng D, **Qin Q**, **Peng Y**, **Zhong H**, **Huang Y**, **Wang H**, **Tan Q** and **Li Y** (2024) Pre-COVID-19 short sleep duration and eveningness chronotype are associated with incident suicidal ideation during COVID-19 pandemic in medical students: a retrospective cohort study. *Frontiers in Public Health* **12**, 1406396.

Zhou S-J, **Wang L-L**, **Qi M**, **Yang X-J**, **Gao L**, **Zhang S-Y**, **Zhang L-G**, **Yang R** and **Chen J-X** (2021) Depression, Anxiety, and Suicidal Ideation in Chinese University Students During the COVID-19 Pandemic. *Frontiers in Psychology* **12**, 669833.

Zhu J, **Li B**, **Hao F**, **Luo L**, **Yue S**, **Zhai J**, **Chen M**, **Liu Y**, **Liu D** and **Wang J** (2022) Gender-Specific Related Factors for Suicidal Ideation During COVID-19 Pandemic Lockdown Among 5,175 Chinese Adolescents. *Frontiers in Public Health* **10**, 810101.

Zhu S and **Ching WW** (2021) What matters for adolescent suicidality: depressive symptoms or fixed mindsets? Examination of cross-sectional and longitudinal associations between fixed mindset and suicidal ideation. PsyArXiv. Available at: https://osf.io/n5x9z (accessed 15 August 2025).

Zhu S, **Zhuang Y**, **Lee P** and **Wong PWC** (2021) The changes of suicidal ideation status among young people in Hong Kong during COVID-19: A longitudinal survey. *Journal of Affective*

Disorders **294**, 151–158.

Žilinskas E, **Žulpaitė G**, **Puteikis K** and **Viliūnienė R** (2021) Mental Health among Higher Education Students during the COVID-19 Pandemic: A Cross-Sectional Survey from Lithuania. *International Journal of Environmental Research and Public Health* **18**(23), 12737.

Zisook S, **Doran N**, **Downs N**, **Lee D**, **Nestsiarovich A** and **Davidson JE** (2022) Healthcare provider distress before and since Covid-19. *General Hospital Psychiatry* **79**, 180–182.

Zwickl S, **Angus LM**, **Qi AWF**, **Ginger A**, **Eshin K**, **Cook T**, **Leemaqz SY**, **Dowers E**, **Zajac JD** and **Cheung AS** (2023) The impact of the first three months of the COVID-19 pandemic on the Australian trans community. *International Journal of Transgender Health* **24**(3), 281–291.
